# A pan-vertebrate signaling motif controls the molecular function of intracellular AQP12

**DOI:** 10.1083/jcb.202512040

**Published:** 2026-07-02

**Authors:** François Chauvigné, Marc Catalán-García, Xavier Daura, Roderick Nigel Finn, Joan Cerdà

**Affiliations:** 1 Institute of Marine Sciences, Spanish National Research Council (CSIC), Barcelona, Spain; 2 Institute of Biotechnology and Biomedicine (IBB), Universitat Autònoma de Barcelona, Bellaterra (Cerdanyola del Vallès), Spain; 3 Catalan Institution for Research and Advanced Studies (ICREA), Barcelona, Spain; 4 Centro de Investigación Biomédica en Red de Bioingeniería, Biomateriales y Nanomedicina, Instituto de Salud Carlos III, Bellaterra (Cerdanyola del Vallès), Spain; 5 Department of Biological Sciences, University of Bergen, Bergen, Norway

## Abstract

Unorthodox AQP12-type channels are poorly understood intracellular aquaporins localized in the endoplasmic reticulum and zymogen granules (ZGs) of pancreatic acinar cells. Despite connections to major diseases, their biophysical properties and intracellular trafficking regulation remain largely unknown. Here, we show that heterologously expressed plant and metazoan AQP12-related channels specifically localize to the intracellular yolk platelet (YP) membrane of frog oocytes, a feature that is recapitulated *in vivo* for invertebrate and vertebrate orthologs. Using native YP membranes, we show that the vertebrate channels are mercury-sensitive polytransporters with intracellular trafficking regulated by Ca^2+^ and cAMP signaling pathways. We identify a novel pan-vertebrate C-terminal YP-targeting domain (YPD) in AQP12, which also drives orthodox aquaporin chimeras and truncated channels to YPs and ZGs. In cultured pancreatic cells, the YPD and Ca^2+^-induced AQP12 N-terminal phosphorylation coregulate secretagogue-triggered channel transport to the ZGs for enzyme secretion. These findings uncover conserved signaling mechanisms for AQP12 trafficking to intracellular protein storage vesicles, and open unexpected avenues for targeted delivery systems.

## Teaser

The discovery of a novel trafficking domain in unorthodox AQP12 yields insight into the channel’s native membrane function and its molecular regulation in intracellular yolk platelets and pancreatic zymogen granules.

## Introduction

Since the discovery of the unorthodox or superaquaporins AQP11 and AQP12 >20 years ago (Ishibashi et al., 2000; Itoh et al., 2005), little progress has been made in understanding their molecular regulation and function, despite their association with major diseases. In contrast to the vertebrate orthodox aquaporins (AQP0-10, AQP13-16) that are trafficked to plasma membranes to facilitate the bidirectional flux of water and other small, mostly uncharged molecules down their concentration gradients, AQP11 and AQP12 are primarily expressed intracellularly (Agre et al., 2002; Ishibashi et al., 2021). The mammalian unorthodox aquaporins are typically observed in the endoplasmic reticulum (ER) with AQP11 identified in tissues such as the liver, testis, brain and kidney, while AQP12 is predominantly expressed in the acinar and beta cells of the pancreas with transfer from the ER to the zymogen granule (ZGs) after secretagogue peptide hormone cholecystokinin (CCK) stimulation (Itoh et al., 2005; Gorelick et al., 2006; Koike et al., 2016; Méndez-Giménez, 2017). The ER or ZG localization has rendered the proteins unamenable to biophysical and structural investigation. Some studies have nevertheless utilized non-native proteoliposomes and yeast as expression vectors, or mammalian cultured cells, to report that under such conditions, the channels are permeable to water, hydrogen peroxide (H_2_O_2_), and glycerol (Yakata et al., 2007; Yakata et al., 2011; Madeira et al., 2014; Bjørkskov et al., 2017; Bestetti et al., 2020; Amro et al., 2024). Genetic approaches using mammalian AQP11-null and AQP12-null knockouts have also revealed respective roles in polycystic kidney disease and acute pancreatitis (Morishita et al., 2005; Okada et al., 2008; Ohta et al., 2009; Rojek et al., 2013; Inoue et al., 2014), but the involved causes remain uncertain, particularly for AQP12. The difficulty of isolating the intracellular vesicles of pancreatic acinar cells and the development of *in vitro* cellular systems for functional studies has hindered the elucidation of the biophysical properties of AQP12, as well as the signal transduction pathways governing channel trafficking to the native membranes. The absence of such knowledge has hampered the establishment of AQP12 and its trafficking regulatory factors as potential therapeutic targets for pancreatic diseases.

To address the experimental accessibility limitation for AQP12, here we took advantage of an earlier observation that an invertebrate co-ortholog of vertebrate AQP12 is targeted to the intracellular yolk platelets (YPs) when heterologously expressed in *Xenopus laevis* oocytes (Stavang et al., 2015). The YPs are low-permeable membrane-bound intracellular vesicles that form during vitellogenesis (oocyte growth) in metazoans to store vitellogenin-derived yolk proteins in liquid and “crystalline-like” structures (Opresko et al., 1980; Wallace and Selman 1990; Leonard et al., 1972). The YPs appear to be modified lysosomes (Fagotto 1995), which can also be considered organelles reminiscent of plant vacuoles and the ZGs from pancreatic acinar cells. We initially investigated the orthologous relationships of AQP12-like (Aqp12L) channels in metazoans ranging from sponges to humans, as well as plant small basic intrinsic proteins (SIPs), and found that each heterologously expressed protein is specifically targeted to the YP membrane (YPM) in frog oocytes. This finding allowed us to use the YPs as native membrane models for deciphering the channel permeabilities of both mammalian and piscine AQP12, and the signal transduction pathways regulating their intracellular trafficking. Structure–function–deletion analyses identified a 7–amino acid signal motif in the carboxy terminus (C terminus) of vertebrate AQP12 orthologs, which we term the YP-targeting domain (YPD). The YPD is both necessary and sufficient for targeting the channels to piscine and amphibian YPs, as well as to the ZGs of mammalian pancreatic acinar cells. Chimeric linking of the YPD to other orthodox channels or truncated proteins resulted in their trafficking to the YPs and ZGs. The data further reveal that both the YPD and hormone-triggered phosphorylation of the AQP12 amino terminus (N terminus) control ZG targeting of the channel for enzyme secretion. The findings elucidate the native biophysical properties of AQP12 channels and the signaling mechanisms mediating their transport to intracellular protein storage vesicles, providing novel insight into the intracellular signaling pathways regulating pancreatic acinar function.

## Results

### Metazoan and plant AQP12-like channels are targeted to the YPM when expressed in *X. laevis* oocytes

**Figure S1.**
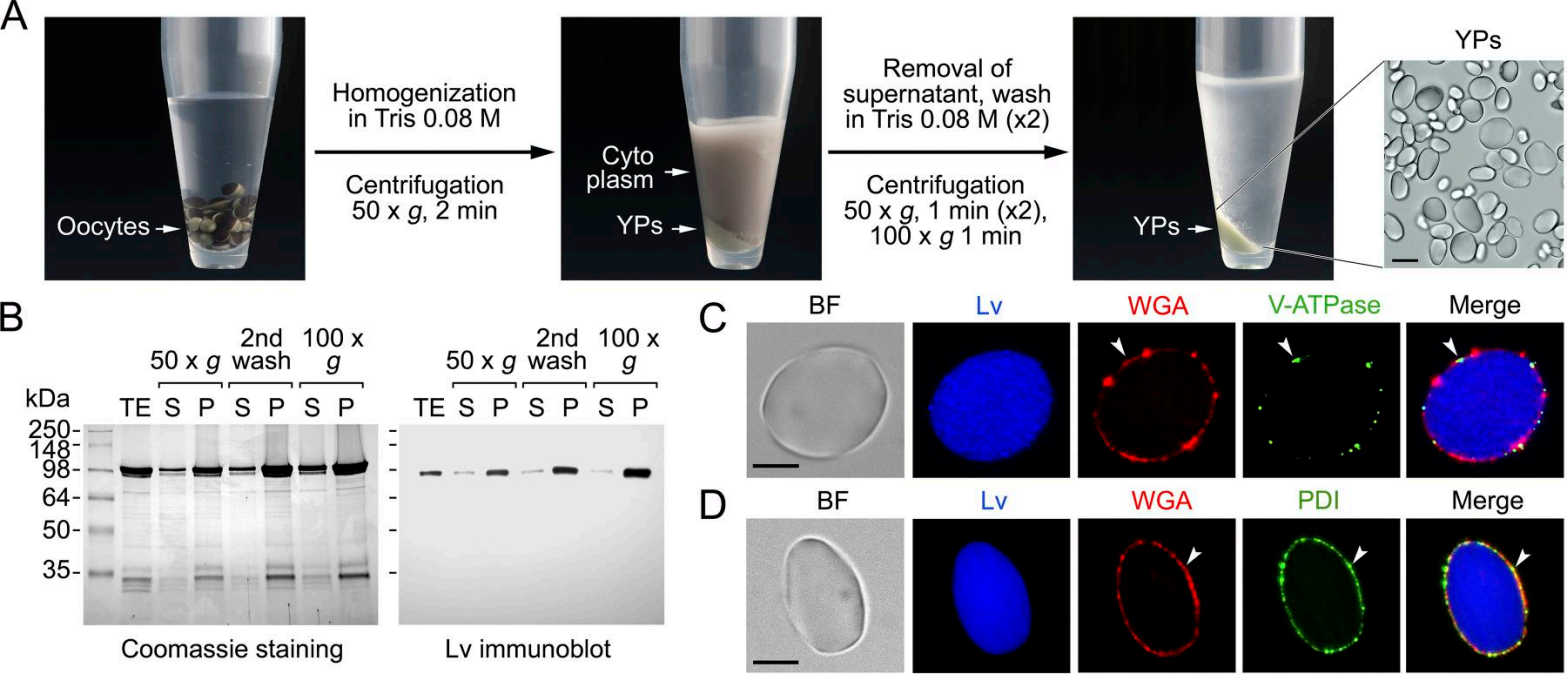
**Procedure for the isolation of intact YPs from *X. laevis* oocytes. (A)** Oocytes (*n* = 30) are homogenized in 200 μl of Tris 80 mM (200 mOsm) at pH 7.5, by gentle pipetting, and the homogenate is centrifuged at 50 × *g* for 2 min at room temperature. The supernatant is discarded, and the pellet is washed twice in 500 μl of Tris 80 mM, pH 7.5, followed by 1-min centrifugation at 50 × *g*. Finally, the pellet is washed in 500 μl of Tris 80 mM, pH 7.5, and centrifuged for 1 min at 100 × *g*. On the right, a bright-field image of the final pellet containing intact YPs is shown. **(B)** SDS-PAGE gel stained with Coomassie blue and immunoblot using an α-Lv antibody of the TE, and supernatants (S) and pellets (P) after the consecutive washings and centrifugations. Molecular mass markers (kDa) are on the left. **(C and D)** Immunofluorescence microscopy images of YPs in which the YPM (arrowheads) is counterstained with fluorescent-conjugated WGA (red), whereas the yolk proteins inside the YPs are labeled with a killifish anti-vitellogenin antibody that stains Lv products (Lv, blue). The YPM is also labeled with vacuolar H^+^-ATPase (V-ATPase) or PDI (both in green), which are known components of this membrane (Fagotto and Maxfield, 1994; Jorgensen et al., 2009). Scale bars, 5 µm. TE, total extracts; Lv, lipovitellin. Source data are available for this figure: SourceData FS1.

**Figure S2.**
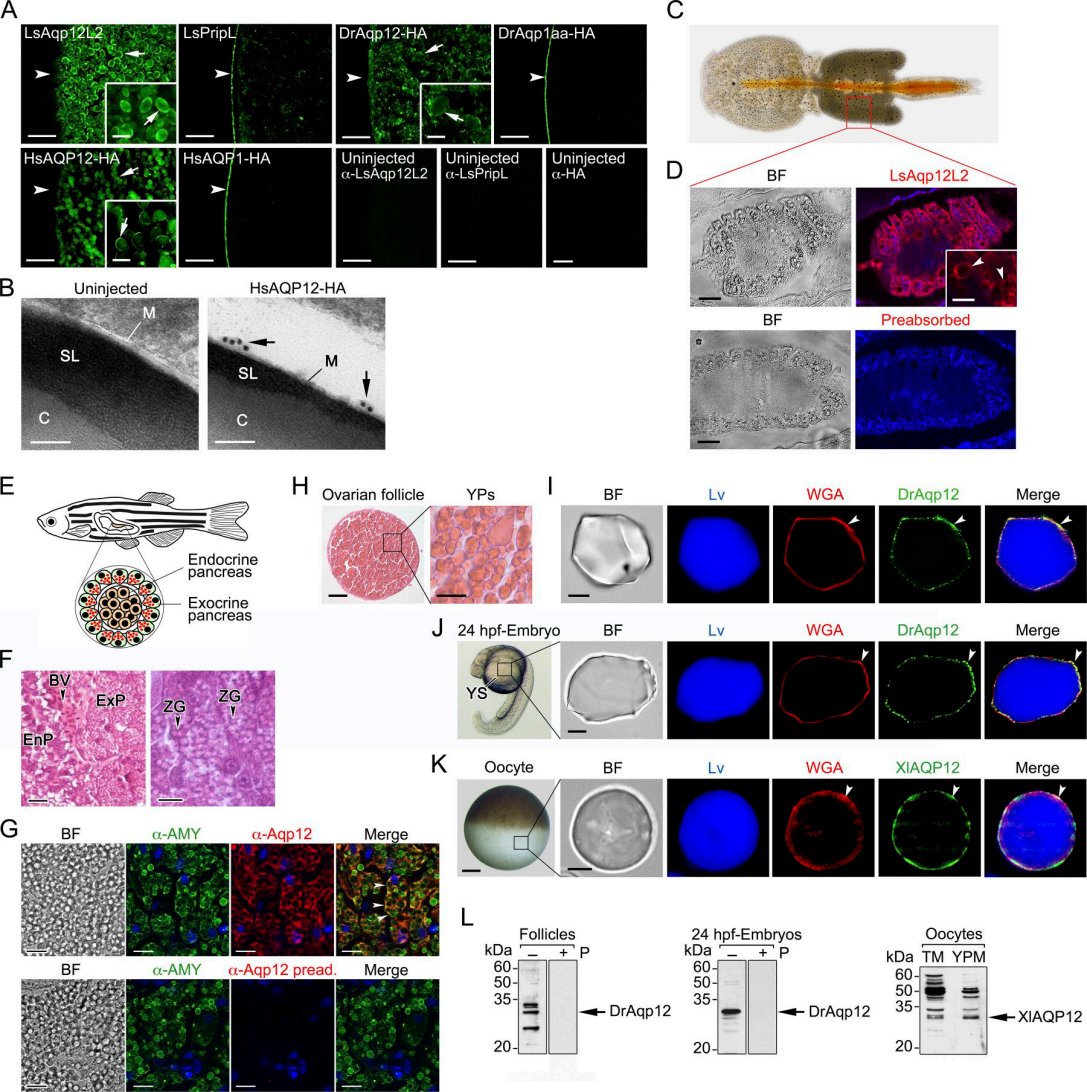
**Exogenous and endogenous AQP12-related channels are targeted to the YPM of yolked oocytes and embryos and membrane of pancreatic ZGs. (A)** Representative immunostaining of *X. laevis* oocytes expressing the salmon louse *L*. *salmonis* Aqp12L2 or PripL (LsAqp12L2 and LsPripL, respectively), HA-tagged human AQP12 or AQP1 (HsAQP12-HA and HsAQP1-HA, respectively), or HA-tagged zebrafish Aqp12 or Aqp1aa (DrAqp12-HA and DrAqp1aa-HA, respectively), using LsAqp12L2-or LsPripL-specific antibodies or an α-HA antibody. The plasma membrane of oocytes is indicated by an arrowhead, whereas the YPM is indicated by arrows. Uninjected oocytes were probed with each of the antibodies as indicated. Scale bars, 20 µm (insets, 10 µm). **(B)** Immunoelectron microscopy micrographs of a YP from a *X. laevis*–uninjected oocyte and an oocyte expressing the HsAQP12 showing immunogold channel particles in the YPM. C, crystalline core of yolk proteins; SL, superficial layer. Scale bars, 100 nm. **(C)** Stereomicroscopic image of an adult salmon louse female. Unmodified image taken from Catalán-García et al. (2021). **(D)** Immunostaining of LsAqp12L2 (red) in the ovary. The nuclei were counterstained with DAPI (blue), and the YPM is indicated by arrowheads. Control sections were incubated with the preabsorbed antisera. Scale bars, 50 µm (inset, 20 µm). **(E)** Schematic diagram of the localization and structure of the adult zebrafish pancreas. ExP, exocrine pancreas; EdP endocrine pancreas. **(F)** Histological section across the exocrine and endocrine pancreatic tissue (left panel), and detail of the acinar cells showing the ZGs (right panel). Scale bars, 50 and 10 µm, respectively. **(G)** Upper panels: Double immunostaining of the pancreatic acinar cells using a custom-made zebrafish Aqp12-specific antibody (Table S1) and the α-AMY antibody (red and green color, respectively) showing the immunolocalization of the channel in the membrane of the ZGs (arrowheads). Lower panels: Control sections incubated with the preabsorbed antiserum were negative. In both sections, nuclei were counterstained with DAPI (blue). Scale bars, 10 µm. **(H)** Histological section of a zebrafish vitellogenic ovarian follicle stained with hematoxylin and eosin (left), and high-magnification image of the oocyte YPs (right). Scale bars, 100 and 25 µm, respectively. **(I)** Immunolocalization of endogenous Aqp12 in isolated YPs from zebrafish oocytes using the α-Aqp12 antibody (green). Scale bar, 10 µm. **(J and K)** Stereomicroscopic image of a 24-hpf zebrafish embryo (J) and a *X. laevis* postvitellogenic oocyte (K), and immunostaining of endogenous AQP12 channels in the corresponding isolated YPs using the zebrafish Aqp12-specific antiserum or a commercial antibody for human AQP12, respectively. In I, J, and K, the YPs were counterstained with the α-Lv antibody (blue) and WGA (red). BF, bright field; YS, yolk sac. Scale bars, 100 µm (K, left) and 5 µm (I, J, right; and K, right). **(L)** Immunoblot of zebrafish ovarian follicles and 24-hpf embryos and frog oocytes probed with the same antibodies. In each panel, the blots on the right (+) were incubated with the preabsorbed Aqp12 antiserum. The arrows indicate the aquaporin monomers, and molecular mass markers (kDa) are on the left. Source data are available for this figure: SourceData FS2.

Previous functional characterization of the aquaporin repertoire in the parasitic copepod salmon louse (*Lepeophtheirus salmonis*) (Stavang et al., 2015) revealed that an aquaporin related to human AQP12, termed LsAqp12L2, was apparently targeted to the YPs when heterologously expressed in *X. laevis* oocytes. Since this *ex vivo* oocyte YP localization has not been previously reported for any animal aquaporin, we initially investigated whether this specific localization is restricted to LsAqp12L2 or is also shared by vertebrate orthologs, such as human and fish AQP12. We expressed wild-type LsAqp12L2 and human influenza hemagglutinin (HA)-tagged human (*Homo sapiens*) and zebrafish (*Danio rerio*) Aqp12 (HsAQP12-HA and DrAqp12-HA, respectively) in *X. laevis* oocytes, and analyzed the subcellular localization of the protein products by immunofluorescence and immunogold transmission electron microscopy (TEM) using LsAqp12L2-specific antibodies or an anti-HA antibody. The localization of the channels was also determined by immunoblotting of protein extracts from oocyte total membranes, as well as from membranes extracted from YPs purified by sequential centrifugation, taking advantage of the very high density of these organelles (Fig. S1). To rule out an artifactual subcellular localization of the expressed channels, additional oocytes were injected with cRNAs coding for aquaporins constitutively expressed in the oocyte plasma membrane from the same species, including LsPripL, HsAQP1-HA, and DrAqp1aa-HA (Preston et al., 1994; Ferré et al., 2023; Stavang et al., 2015), and their localization analyzed as above. For the three species, the AQP12 channels specifically accumulated in the oocyte YPM, while the AQP1-type orthologs were exclusively localized at the oocyte surface (Fig. S2, A and B).

**Figure 1.**
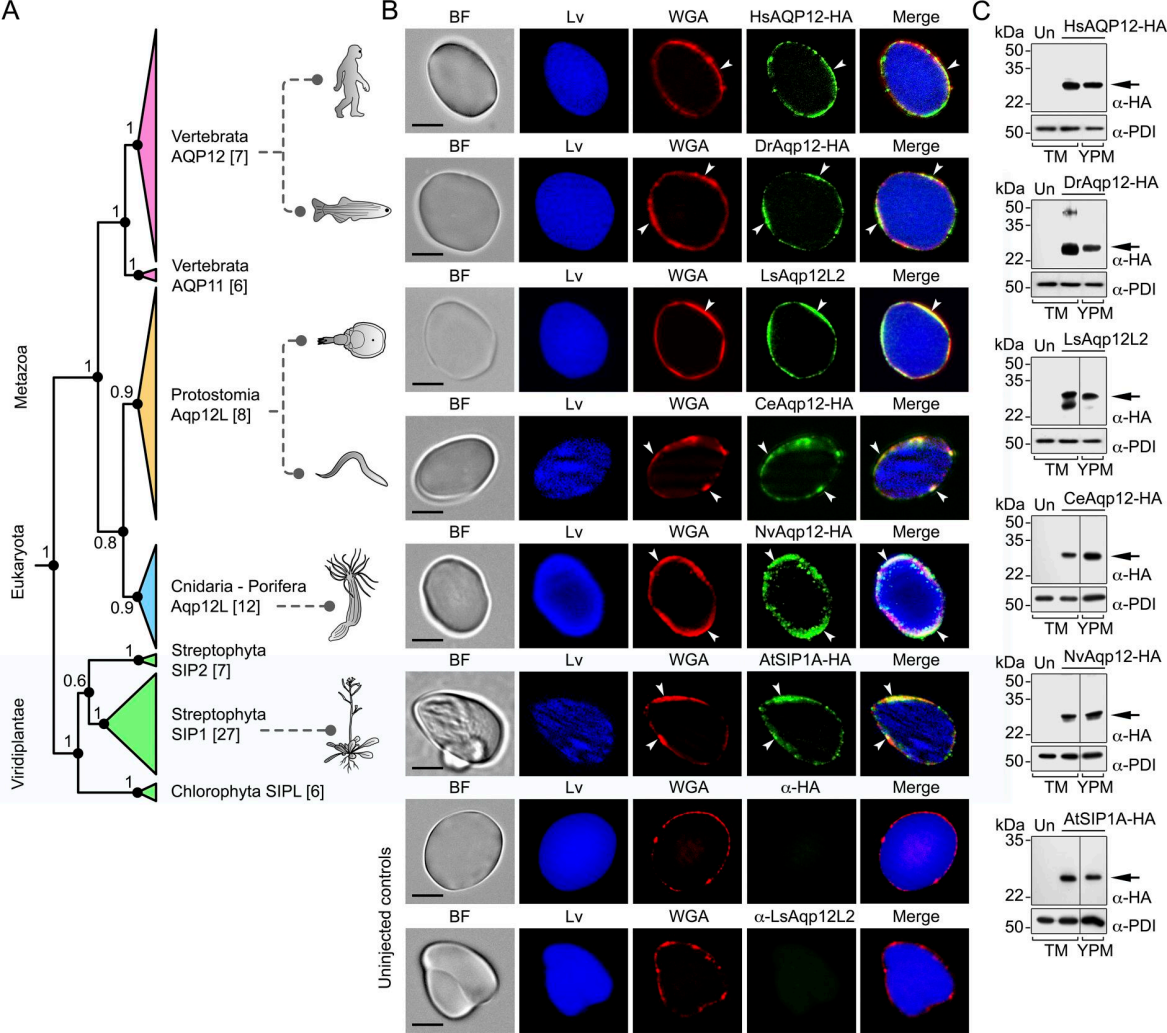
**Metazoan and plant AQP12-like orthologs are targeted to the YPM when expressed in *X. laevis* oocytes. (A)** Bayesian majority rule consensus tree of the unorthodox AQP12-related channel CDS of Viridiplantae and Metazoa. The tree is inferred from 10 million MCMC generations and midpoint rooted with posterior probabilities shown at each node. The number of taxa in each triangle is indicated in square parentheses. **(B)** Representative immunostaining of HA-tagged AQP12-related orthologs (green) in YPs isolated from *X. laevis* oocytes expressing human (HsAQP12A-HA), zebrafish (DrAqp12-HA), nematode (CeAqp12-HA), jellyfish (NvAqp12-HA), thale cress (AtSIP1A-HA), and untagged Aqp12L2 from the salmon louse (LsAqp12L2), using an α-HA antibody or an α-LsAqp12L2–specific antibody. The YPM (arrowheads) is counterstained with WGA (red), whereas the intra-YP yolk proteins are labeled with an α-Lv antibody (blue). The lower two panels show YPs extracted from uninjected oocytes (controls) probed with the α-HA or α- LsAqp12L2 antibodies. BF, bright field. Scale bars, 5 µm. **(C)** Immunoblot of protein extracts from TM and YPM of uninjected oocytes or oocytes expressing the different AQP12-related orthologs using the antibodies specified above. The α-PDI antibody was used as a protein loading control. The arrows indicate the aquaporin monomers. Molecular mass markers (kDa) are on the left. TM, total membrane. Source data are available for this figure: SourceData F1.

To investigate whether the observed AQP12 YPM localization is a wider feature of AQP12-related channels, we initially used Bayesian inference to determine the orthologous relationships of the unorthodox proteins and coding sequences of metazoans ranging from sponges to humans with those of green alga and plant (Viridiplantae) SIPs. The results show that both the amino acid and codon-aligned Metazoan AQP12- and Viridiplantae (chlorophyte and streptophyte) SIP-related channels form a sister clade of unorthodox channels separated from the orthodox channels, encompassing the aquaglyceroporins, AqpM, AqpN, nodulin 26–like intrinsic proteins, AqpZ, and AQP8- and AQP4-related sequences of prokaryotes, plants, fungi, and metazoans (Fig. 1 A). Since humans and some invertebrates (nematodes and salmon louse) encode two or more AQP12-type channels, and vertebrates also encode unorthodox AQP11-type channels with two copies in teleost fishes, the vertebrate AQP12-type channels are co-orthologs of the invertebrate forms. Similarly, streptophyte plants encode multiple SIPs, and we therefore consider these channels co-orthologs of the metazoan unorthodox channels. Immunolocalization experiments using isolated YPs and an anti-lipovitellin antibody and fluorescent-conjugated wheat germ agglutinin (WGA), as markers of yolk proteins and YPM, respectively, as well as immunoblot analyses, using the YPM component protein disulfide isomerase (PDI) (Jorgensen et al., 2009) as endogenous control, revealed that each selected co-ortholog from plant and metazoans is specifically targeted to the YPM when expressed in *X. laevis* oocytes (Fig. 1, B and C).

The *in vivo* YPM localization of endogenous AQP12 channels was further confirmed in the ovary of the salmon louse, in isolated vitellogenic oocytes of the zebrafish and *X. laevis*, and in the yolk sac of 24-h postfertilization zebrafish embryos using species-specific or commercial AQP12 antibodies (Fig. S2, C, D, and H–L). These data therefore indicate that intracellular targeting of AQP12 channels to the YPs is a conserved feature in yolk-retaining oocytes and embryos from invertebrates and vertebrates.

### Human and zebrafish AQP12 channels are mercury-sensitive polytransporters

**Figure 2.**
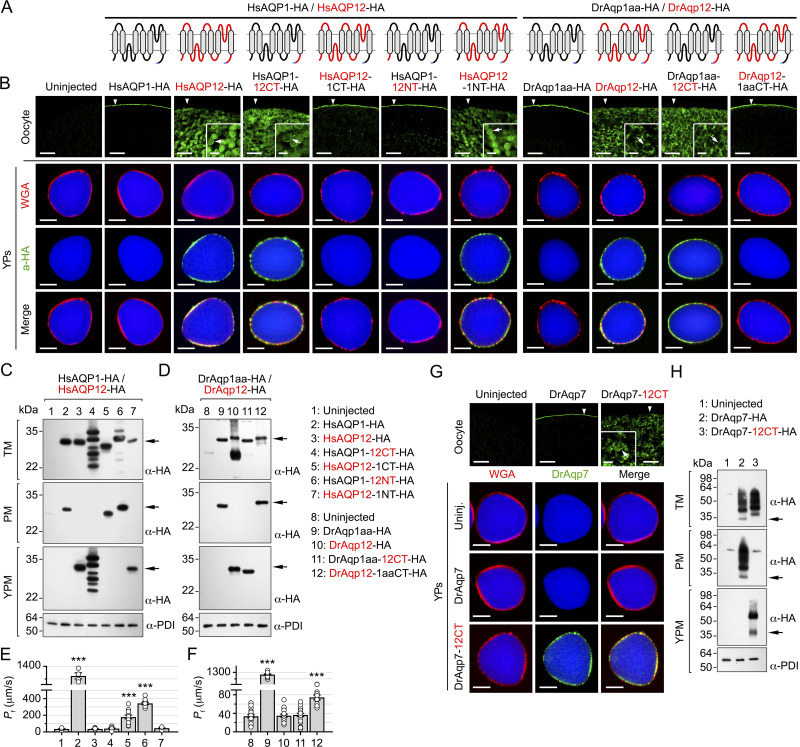
**AQP12 C terminus is involved in channel targeting to the YPM of *X. laevis* oocytes. (A)** Schematic illustrating the structure of wild-type HsAQP1 (black), HsAQP12 (red), DrAqp1aa (black), and DrAqp12 (red), or the chimeric constructs in which the complete N or C termini of AQP1 were exchanged with those of AQP12, and vice versa. **(B)** Immunostaining of uninjected oocytes and oocytes expressing the different HA-tagged constructs on oocyte sections and corresponding isolated YPs. The YPs were counterstained with α-Lv antibodies and WGA. In the upper panels, the arrowheads indicate the oocyte plasma membrane, whereas the arrows indicate the YPM. Scale bars, 50 µm (oocyte sections), 10 µm (insets), and 5 µm (YPs). **(C and D)** Immunoblots of TM, plasma membrane (PM), and YPM extracts from uninjected oocytes and oocytes expressing the different human and zebrafish constructs using α-HA antibody and an α-PDI as a loading control. The arrows indicate aquaporin monomers, and molecular mass markers (kDa) are on the left. **(E and F)** Osmotic water permeability (*P*_f_) of oocytes (mean ± SEM; *n* = 4–12 oocytes per group) expressing the constructs indicated in Fig. 2, C and D. The asterisks denote statistically significant differences with respect to uninjected oocytes (unpaired Student *t* test; ***P < 0.001). **(G)** Immunostaining of whole oocytes and isolated YPs from oocytes expressing HA-tagged wild-type Aqp7 (DrAqp7-HA) or the chimeric construct carrying the complete Aqp12 C terminus (DrAqp7-12CT-HA). The YPs were counterstained as above. In the upper panels, the arrowheads indicate the oocyte plasma membrane, whereas the arrows indicate the YPM. Scale bars, 50 µm (oocyte sections) and 5 µm (YPs). **(H)** Immunoblots of TM, PM, and YPM extracts from oocytes expressing the two constructs using the α-HA and α-PDI antibodies. The arrows indicate aquaporin monomers, and molecular mass markers (kDa) are on the left. Source data are available for this figure: SourceData F2.

Since aquaporin intracellular trafficking is often regulated by specific motifs located in the N and C terminus, respectively, of the channels, we investigated the existence of a YP-targeting signal in the amino acid sequence of these regions in HsAQP12 and DrAqp12. To assess this, cDNA-encoding HA-tagged chimeric HsAQP12 and HsAQP1 proteins, or DrAqp12 and DrAqp1aa, in which the N- and/or C-terminal domains were exchanged were synthesized and the corresponding cRNAs expressed in *X. laevis* oocytes (Fig. 2 A). Oocytes expressing wild-type HsAQP1-HA or HsAQP12-HA showed plasma membrane and YPM localization, respectively, whereas the interchange of their C termini, but not of the N termini, switched the subcellular localization of HsAQP1 to the YPM and of HsAQP12 to the plasma membrane (Fig. 2 B). Identical results were obtained when the C termini of DrAqp12 and DrAqp1aa were interchanged (Fig. 2 B). These observations were confirmed by immunoblotting (Fig. 2, C and D) and oocyte swelling assays (Fig. 2, E and F), despite chimeric HsAQP1-12CT showing lower stability as indicated by the detection of multiple bands possibly as a result of partial degradation. Interestingly, the interchange of the C terminus of the zebrafish aquaglyceroporin Aqp7 with that of DrAqp12 also shifts channel targeting from the oocyte plasma membrane to the YPM (Fig. 2, G and H). These data show that the HsAQP12 and DrAqp12 C termini are mechanistically associated with the targeting of the channels to the YPM of amphibian oocytes.

**Figure 3.**
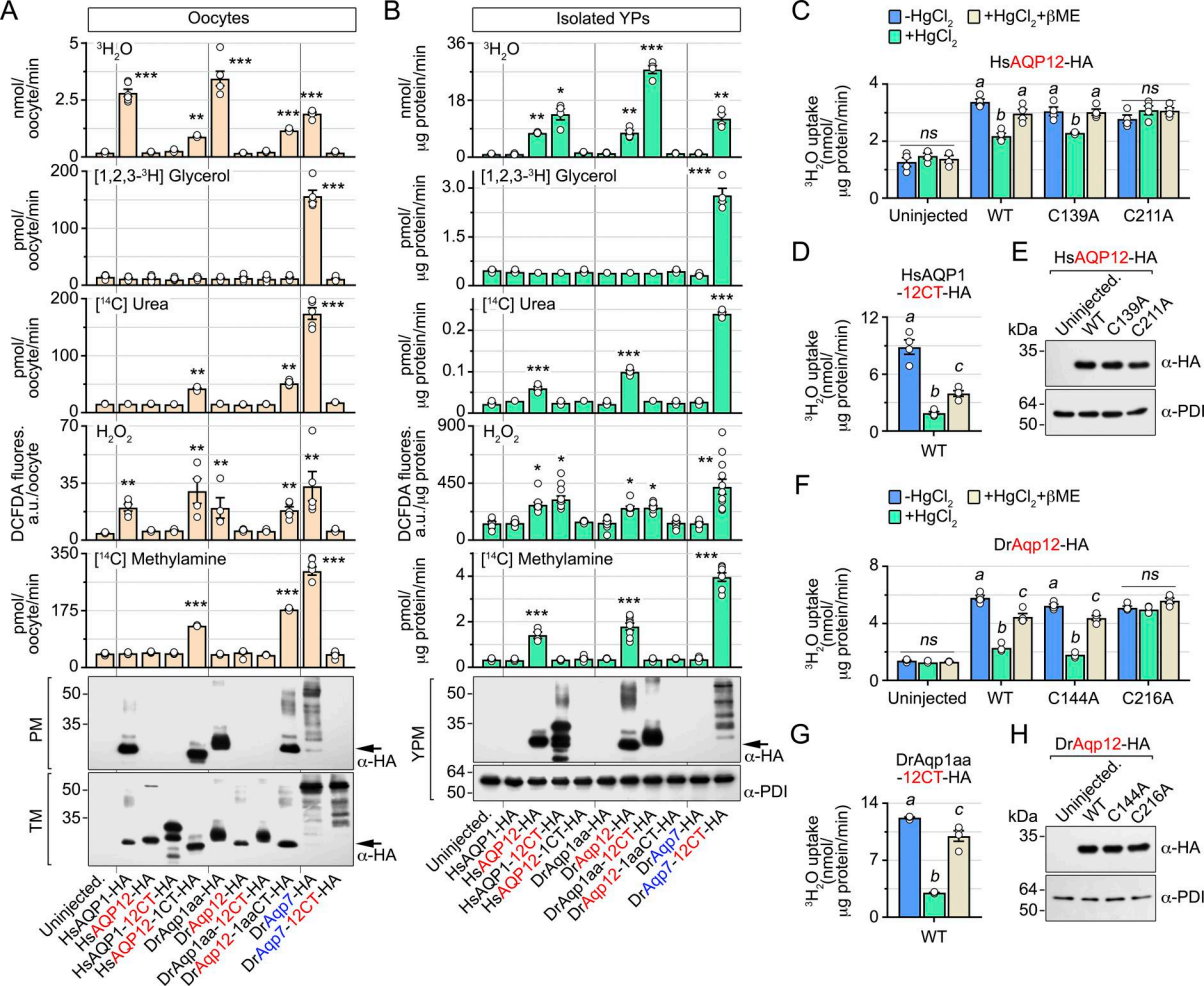
**Mammalian and piscine AQP12 channels are mercury-sensitive polytransporters. (A)** Water and solute (glycerol, urea, H_2_O_2_, and ammonia) permeability of *X. laevis*–uninjected oocytes and oocytes expressing HA-tagged WT HsAQP1, HsAQP12, DrAqp1aa or DrAqp12, or chimeric channels in which the entire CT of the AQP1 and AQP12 paralogs from the same species were swapped. Oocytes expressing the WT or chimeric DrAqp7 (DrAqp7-HA and DrAqp7-12CT-HA, respectively) were used as positive controls for solute transport. **(B)** Water and solute permeability of YPs isolated from oocytes injected with the different constructs as in A. In A and B, the bottom panels show representative immunoblots of TM, PM, and YPM protein extracts from the oocytes using the α-HA antibody and the α-PDI antibody as a loading control. In A and B, data (mean ± SEM, *n* = 5–8 oocytes in A, and *n* = 4–12 biological replicates combined from two independent experiments in B) were statistically analyzed by unpaired Student’s *t* test (*P < 0.05; **P < 0.01; ***P < 0.001; with respect to the controls). **(C, D, F, and G)** Mercurial inhibition (100 µM HgCl_2_) of water permeability, and reversibility by βME, of YPs expressing WT HsAQP12-HA (C) or DrAqp12-HA (F), or mutant channels at potential mercury-binding Cys residues. YP-targeted HsAQP1-12CT-HA (D) or DrAqp1aa-12CT-HA (G) were also tested as positive controls for mercury inhibition. For each construct, data (mean ± SEM, *n* = 4 independent experiments) were statistically analyzed by one-way ANOVA followed by Tukey’s multiple comparisons test. Bars with different superscripts are significantly different (P < 0.05). **(E and H)** Immunoblots of the different constructs in the YPM, normalized to PDI. Molecular mass markers (kDa) are on the left. CT, C termini; WT, wild type; βME, β-mercaptoethanol. Source data are available for this figure: SourceData F3.

The chimeric YPM-targeted water-selective aquaporins and aquaglyceroporins were used as positive controls to investigate the permeability properties of human and piscine AQP12 channels using *X. laevis* oocytes. Oocytes were injected with wild-type HsAQP1-HA, HsAQP12-HA, DrAqp1aa-HA, DrAqp12-HA, DrAqp7-HA, or their C-terminal chimeric forms as above, and their permeability, as well as that of the isolated YPs, to water, glycerol, urea, urea, methylamine (an ammonia analog), and H_2_O_2_ was determined using radioactive or fluorescent substrate uptake assays. Immunoblotting of the subcellular fractions of oocytes expressing each construct using PDI as an endogenous control was performed in parallel to assure that the different channels were localized in the plasma membrane or the YPM as expected from earlier experiments. Oocytes expressing either wild-type HsAQP1-HA or DrAqp1aa-HA at the oocyte surface were permeable to water and H_2_O_2_, whereas those expressing wild-type DrAqp7-HA were also permeable to glycerol, urea, and the ammonia analog methylamine (Fig. 3 A), in agreement with the known properties of these channels (Litman et al., 2009; Tingaud-Sequeira et al., 2010; Almasalmeh et al., 2014). In contrast, oocytes injected with wild-type HsAQP12 or DrAqp12, or HsAQP1, DrAqp1aa, and DrAqp7 chimeric constructs bearing the HsAQP12 or DrAqp12 C termini (HsAQP12-HA, DrAqp12-HA, HsAQP1-12CT-HA, DrAqp1aa-12CT-HA, and DrAqp7-12CT-HA), which remained intracellular, did not incorporate water or any of the solutes tested (Fig. 3 A). However, the oocytes expressing plasma membrane–targeted AQP12 chimeric channels (HsAQP12-1CT-HA and DrAqp12-1aaCT-HA) showed an increase of water, urea, H_2_O_2_, and methylamine uptake with respect to uninjected (control) oocytes (Fig. 3 A). In addition, YPs isolated from oocytes expressing HsAQP12-HA or DrAqp12-HA were also more permeable to water, urea, H_2_O_2_, and methylamine compared with the control YPs, while the YPs from HsAQP1-12CT-HA, DrAqp1aa-12CT-HA, and DrAqp7-12CT-HA oocytes, expressing YPM-targeted chimeric channels, showed the same permeability properties as those observed in oocytes expressing the wild-type channels in the plasma membrane (Fig. 3 B). Accordingly, the uptake of water and solutes by YPs from oocytes injected with HsAQP1-HA, DrAqp1aa-HA, or DrAqp7-HA did not differ from that of the control YPs (Fig. 3 B). These data indicate that in contrast to the AQP1-type aquaporins, human and piscine AQP12 channels facilitate the molecular permeation of urea and ammonia, in addition to water and H_2_O_2_.

Water and solute conductance of many aquaporins, including HsAQP1 and DrAqp1aa, are inhibited by mercurial ions (Preston et al., 1993; Hirano et al., 2010; Tingaud-Sequeira et al., 2010) by binding to cysteine residues lining the pore (Preston et al., 1993; Sui et al., 2001), which can be reversed by reducing compounds such as β-mercaptoethanol. *In silico* studies on HsAQP12 have predicted that pore-exposed Cys^139^ (and its equivalent Cys^144^ in DrAqp12) may be implicated in mercurial sensitivity of the channel (Calvanese et al., 2013). However, another Cys downstream of the second loop E hemihelix bearing the second NPA motif (Cys^211^ and Cys^216^, in HsAQP12, and DrAqp12, respectively) may also play a structural role (Calvanese et al., 2013). To test both hypotheses, we measured the water permeability of YPs containing wild-type HsAQP12-HA or DrAqp12-HA, or mutant channels in which each of the predicted mercury-targeted Cys was replaced by Ala residues (HsAQP1-C139A-HA, HsAQP12-C211A-HA, DrAqp12-C144A-HA, and DrAqp12-C216A-HA), in the presence or absence of 100 µM mercury chloride (HgCl_2_) with or without 5 mM mercaptoethanol to examine the reversibility of mercury inhibition. As positive controls, we employed YPs with YPM-targeted HsAQP1-12CT-HA or DrAqp1aa-12CT-HA chimeras. HgCl_2_ inhibited the ^3^H_2_O permeabilities of YPs expressing HsAQP12-HA, HsAQP12-C139A-HA, or HsAQP1-12CT-HA by 36%, 25%, and 79%, respectively, whereas that of YPs expressing the HsAQP12-C211A-HA mutant was not affected by mercury (Fig. 3, C and D). Similar results were obtained for the zebrafish Aqp12 constructs, although in this case HgCl_2_ was more effective at reducing water permeability of DrAqp12-HA and DrAqp12-C144A-HA (60% and 65% inhibition, respectively) (Fig. 3 F), while it was equally potent at inhibiting that of DrAqp1aa-12CT-HA (75% inhibition) (Fig. 3 G). As previously observed, mercury had no effect on DrAqp12-C216A-HA–mediated ^3^H_2_O permeability (Fig. 3 F). In all cases, the inhibitory effects of HgCl_2_ were fully or partially recovered with β-mercaptoethanol (Fig. 3, C, D, F, and G), while immunoblotting showed that all mutants were expressed in the YPM (Fig. 3, E and H). These data therefore indicate that both human and piscine AQP12 co-orthologs are mercury-sensitive and that HsAQP12 Cys^211^ and DrAqp12 Cys^216^ are the sites for the mercurial inhibition of channel function.

### Identification of a pan-vertebrate AQP12 YP-targeting motif

**Figure 4.**
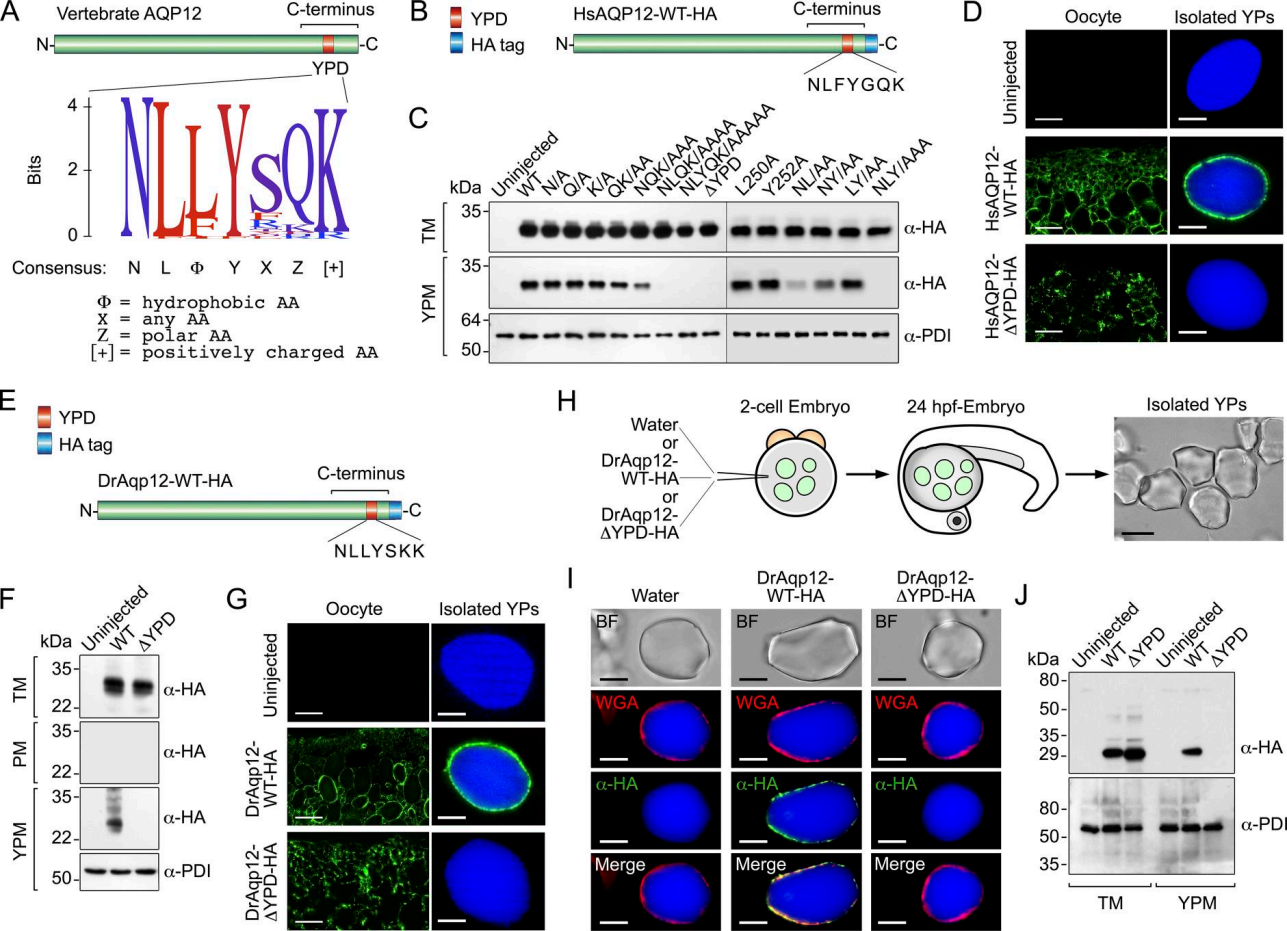
**Identification of a YP-targeting signal in vertebrate AQP12. (A)** Calculated consensus amino acid sequence of the YPD in the AQP12 C terminus after the amino acid sequence alignment of 473 gnathostome AQP12 orthologs. AA, amino acid. **(B)** Diagram of HA-tagged WT HsAQP12 (HsAQP12-WT-HA) showing the YPD amino acid sequence. **(C)** Western blot of TM and YPM protein extracts from *X. laevis*–uninjected oocytes or oocytes expressing HsAQP12-WT-HA or HsAQP12-HA mutants in which different residues comprising the YPD were substituted by Ala, or the complete YPD replaced by Ala residues (ΔYPD). The immunoblots were probed with α-HA or α-PDI antibodies. **(D)** Immunolocalization of HsAQP12-WT-HA and HsAQP12-ΔYPD-HA (green) in oocytes and isolated YPs. Uninjected control oocytes are shown in the top panels. **(E–G)** YPD amino acid sequence of DrAqp12-HA (E), immunoblotting of TM, PM, and YPM of oocytes expressing the DrAqp12-WT-HA or the DrAqp12-ΔYPD-HA mutant channel (F), and immunolocalization of the constructs in oocytes and YPs, and control oocytes. Part of the blot from F is shown in Fig. 5 F. **(H–J)** Overexpression of DrAqp12-WT-HA or DrAqp12-ΔYPD-HA in zebrafish embryos (H), and further analysis of the localization of the channels in isolated YPs from 24-hpf embryos by immunostaining (I; green) and immunoblotting (J). In D, G, and I, the YPM and yolk proteins were respectively labeled with WGA (red) and the α-Lv antibody (blue). Scales bars, 10 µm (D and G, left panels) and 5 µm (D and G, right panels, and I). In C, F, and J, molecular mass markers (kDa) are on the left. hpf, hours postfertilization; WT, wild type. Source data are available for this figure: SourceData F4.

To identify the C-terminal region that mediates the targeting of AQP12 to the YPM of frog oocytes, we aligned the amino acid sequences of AQP12 orthologs from 473 vertebrate taxa. This analysis revealed a short conserved domain of seven amino acids in the C termini of the channels with a calculated consensus sequence defined as NLΦYXΖ[+], where Φ is a hydrophobic amino acid, X is any amino acid, Ζ is a polar amino acid, and [+] is a positively charged amino acid (Fig. 4 A). We therefore termed this conserved sequence as the putative YPD. To assess the functional role of this domain, point mutations were introduced in the HsAQP12 YPD sequence (NLFYGQK) and the different mutants expressed in *X. laevis* oocytes (Fig. 4 B). Immunoblotting data showed that single substitutions of YPD residues by Ala did not disrupt YPM localization of the channel, whereas this was reduced or completely abolished when two or more of the most conserved domain residues were replaced by Ala (Fig. 4 C). Accordingly, the complete deletion of the YPD (HsAQP12-ΔYPD-HA) entirely prevented the accumulation of the channel in the YPM (Fig. 4 C), which was confirmed by immunostaining of the channel in oocyte sections and isolated YPs (Fig. 4 D). The consensus sequence NLΦYXΖ[+] also constitutes a YP-targeting signal in piscine AQP12 orthologs, since a DrAqp12-HA construct lacking its YPD (NLLYSKK) (DrAqp12-ΔYPD-HA) (Fig. 4 E) lost YPM localization (Fig. 4, F and G), as observed for the human channel.

To verify that the YPD is also functional *in vivo*, we injected cRNAs coding for wild-type DrAqp12-HA or DrAqp12-ΔYPD-HA into one- to two-cell-stage zebrafish embryos, and isolated the YPs from the yolk sac ∼24 h later, when embryos reached the 20-somite stage (Fig. 4 H). Immunostaining and immunoblotting of DrAqp12 constructs in WGA-labeled YPs using the HA antibody, which does not detect the endogenous channel, showed that DrAqp12-HA was inserted in the YPM, whereas YPM targeting was prevented in the DrAqp12-ΔYPD-HA mutant (Fig. 4, I and J). These results confirm that the NLΦYXΖ[+] consensus motif from vertebrate AQP12 constitutes a functional signaling domain that specifically targets YPM localization of the channels in yolked oocytes and embryos.

### The YPD is sufficient to confer YPM targeting of constitutive plasma membrane water and glycerol channels

**Figure 5.**
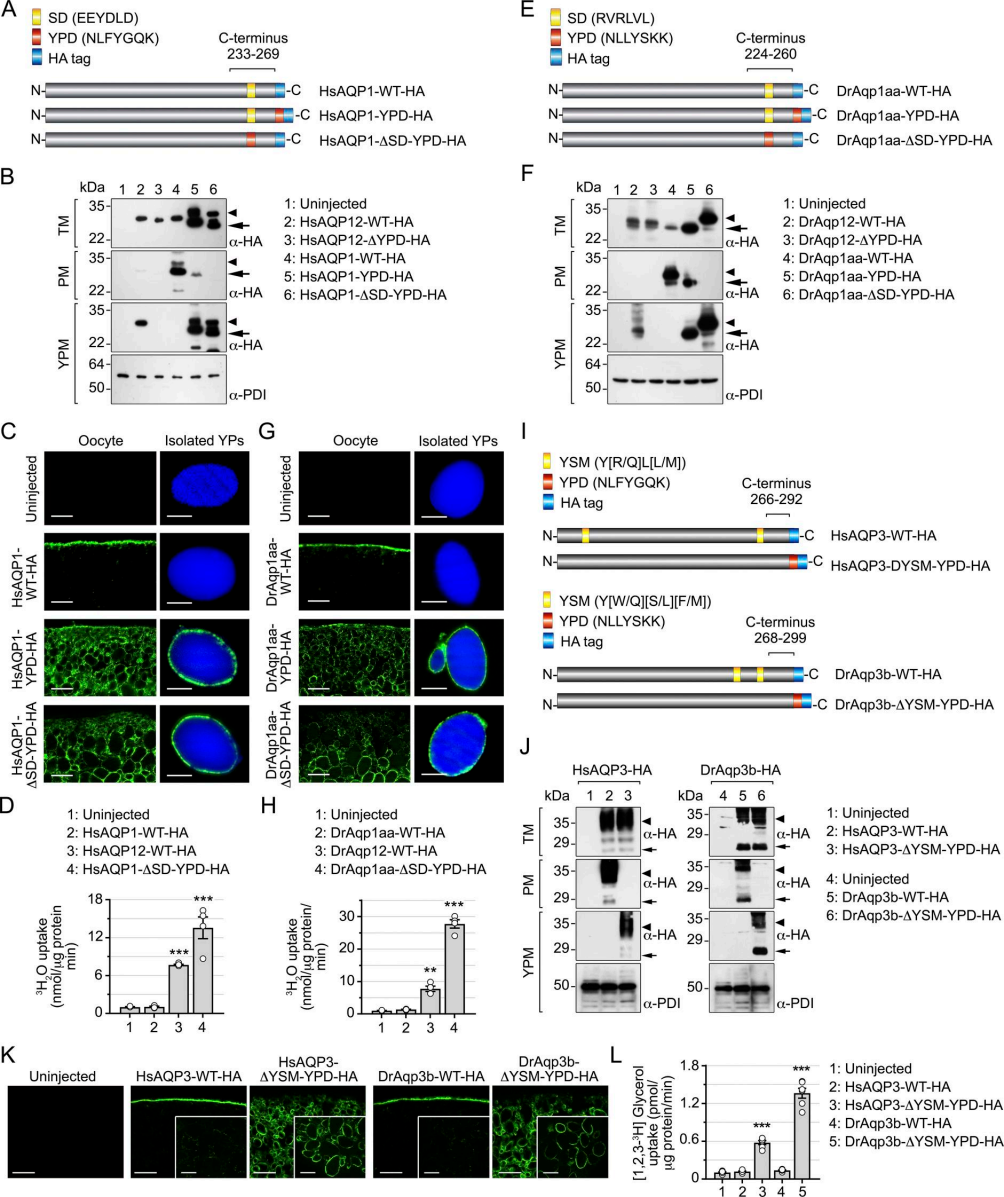
**YPD is sufficient to confer YPM targeting of constitutive plasma membrane AQP1 and AQP3 channels. (A and E)** Schematic diagrams of different HA-tagged HsAQP1 (A) and DrAqp1aa (E) constructs in which the species-specific YPD was inserted at the end of the C termini of the proteins (HsAQP1-YPD-HA and DrAqp1aa-YPD-HA), or by substituting the SD (HsAQP1-ΔSD-YPD-HA and DrAqp1aa-ΔSD-YPD-HA). **(B and F)** Immunoblots of TM, PM, and YPM protein extracts from *X. laevis–*uninjected (control) oocytes or expressing HsAQP12-WT-HA, HsAQP12-ΔYPD-HA, DrAqp12-WT-HA, or DrAqp12-ΔYPD-HA as controls, or the different HsAQP1 or DrAqp1aa mutant constructs. Blots were probed with α-HA or α-PDI antibodies. Part of the blot from F is shown in Fig. 4 F. Arrows indicate aquaporin monomers, whereas the arrowheads indicate likely posttranslational modifications of the channels. Molecular mass markers (kDa) are on the left. **(C and G)** Immunolocalization of HsAQP1-WT-HA and the two YPDs carrying HsAQP1 channels (C), or DrAqp1aa-WT-HA and the two YPDs carrying DrAqp1aa constructs (F), in oocytes and isolated YPs, and in controls, as indicated. Scale bars, 10 µm (left panels), 5 µm (right panels). **(D and H)** Water permeability of YPs isolated from oocytes injected with the different human and zebrafish AQP12 and AQP1 constructs. **(I)** Diagrams of HA-tagged HsAQP3-WT (A) and DrAqp3b-WT (D), and constructs generated for each channel in which the YPD was inserted at the end of the C terminus, while the YSMs in each amino acid sequence (indicated in yellow) were erased by point mutations (HsAQP3-ΔYSM-YPD-HA and DrAqp3b-ΔYSM-YPD-HA). **(J)** Immunoblots of TM, PM, and YPM protein extracts from uninjected oocytes or oocytes expressing the different constructs as indicated, using the α-HA antibody. Protein loading was normalized by PDI, while molecular mass markers (kDa) are on the left. Arrows indicate aquaporin monomers, whereas the arrowheads indicate likely posttranslational modifications of the channels. **(K)** Immunolocalization of the different AQP3 constructs (green) in injected and uninjected oocytes. Scale bars, 20 and 10 µm (insets). **(L)** Glycerol permeability of YPs isolated from oocytes expressing the different human and zebrafish AQP3 constructs. In D, H, and L, data (mean ± SEM, *n* = 4 biological replicates from one experiment) were statistically analyzed by unpaired Student’s *t* test (**P < 0.01; ***P < 0.001; with respect to the uninjected oocytes). Source data are available for this figure: SourceData F5.

**Figure S3.**
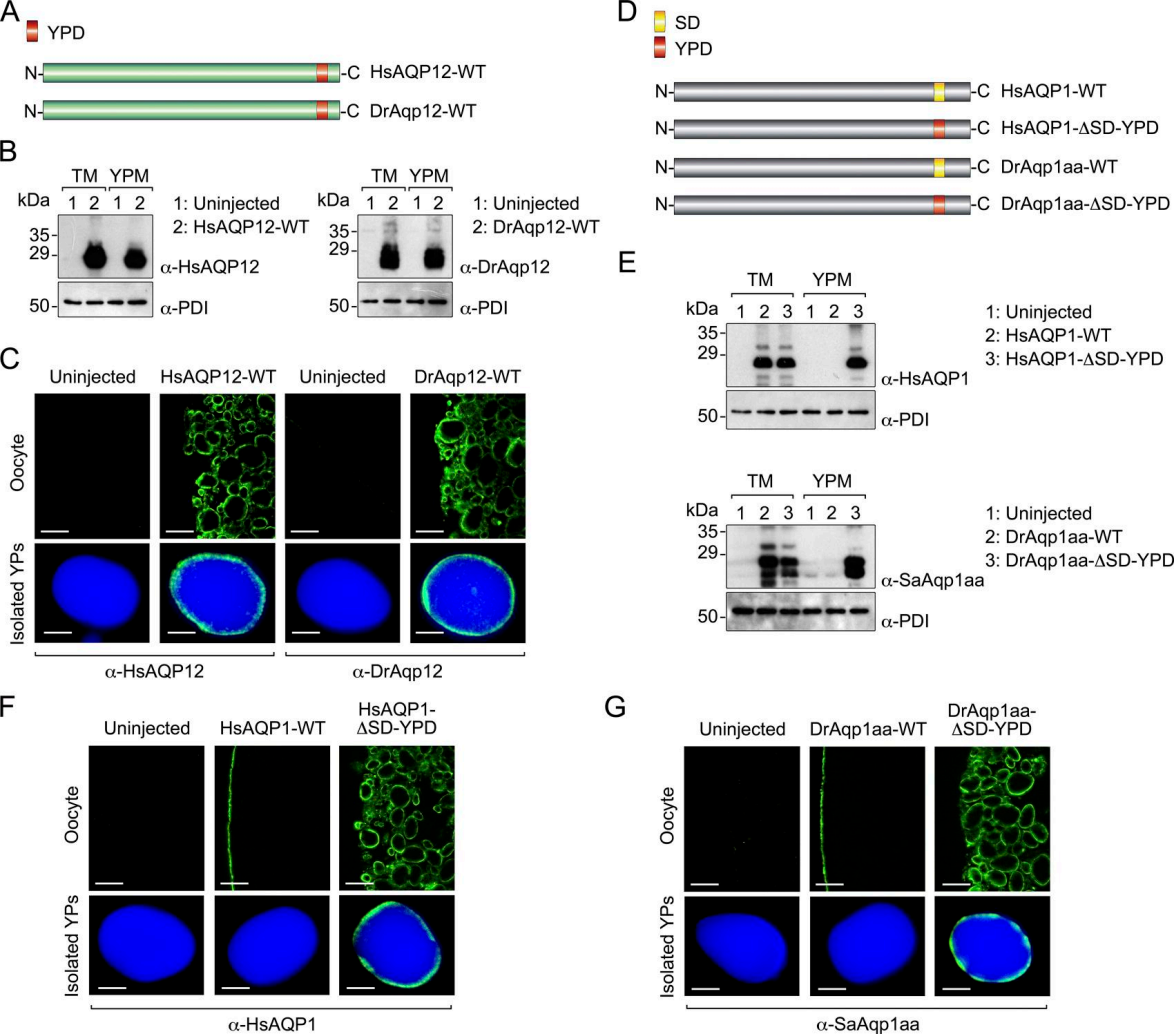
**HA epitope tag in WT AQP12 and AQP1 mutants does not affect YPM channel trafficking. (A)** Schematic diagrams of HsAQP12-WT and DrAqp12-WT without the HA tag. **(B)** Immunoblots of TM and YPM protein extracts from *X. laevis*–uninjected oocytes (control) or expressing HsAQP12-WT or DrAqp12-WT probed with specific antibodies for human AQP12 and zebrafish Aqp12 (Table S1). The α-PDI antibody was used as a protein loading control. **(C)** Immunostaining of oocytes and YPs in control oocytes and expressing the channels. **(D)** Schematic diagrams of HsAQP1, DrAqp1aa, and mutant constructs used in Fig. 5, A and E lacking the HA tag. **(E–G)** Immunoblot (E) and immunostaining (F and G) of the corresponding channels in oocytes and YPs using a human AQP1 and a fish (Gilthead seabream, *Sparus aurata*) Aqp1aa-specific antibody (Table S1), and the α-PDI antibody, as indicated. Molecular mass markers (kDa) are on the left. Scale bars in C, F, and G, 10 µm (upper panels), 5 µm (lower panels). WT, wild type. Source data are available for this figure: SourceData FS3.

We next investigated whether the YPD is sufficient for targeting orthodox aquaporins, such as HsAQP1 and DrAqp1aa, to the YPM of *X. laevis* oocytes. We first added the HsAQP12 YPD to the end of the C terminus of HsAQP1 (HsAQP1-YPD-HA), just preceding the HA-tag sequence (Fig. 5 A). To avoid potential interferences by the constitutive targeting of HsAQP1 to the oocyte plasma membrane, we made another construct in which the YPD replaced a predicted sorting and internalization domain (SD) present in the C terminus of the channel (EEYDLD) (HsAQP1-ΔSD-YPD-HA) (Fig. 5 A). Immunostaining and immunoblot analyses showed that incorporation of the YPD causes targeting of HsAQP1-YPD-HA to the YPM, while the SD maintains some trafficking to the oocyte plasma membrane (Fig. 5, B and C). In contrast, when the YPD replaces the SD the channel is exclusively targeted to the YPM (Fig. 5, B and C). Similarly, the YPD from DrAqp12 was introduced at the end of the DrAqp1aa C terminus (DrAqp1aa-YPD-HA) or in substitution of its predicted SD (RVRLVL) (DrAqp1aa-ΔSD-YPD-HA) (Fig. 5 E). As previously observed for the HsAQP1 constructs, these experiments showed that the YPD drives partial or exclusive YPM localization of DrAqp1aa-YPD-HA and DrAqp1aa-ΔSD-YPD-HA, respectively (Fig. 5, F and G). These observations were corroborated by expressing HA epitope tag-free constructs coding for wild-type HsAQP12 and DrAqp12, and the HsAQP1 and DrAqp1aa mutants, which showed that the tag does not affect channel trafficking to the YPM (Fig. S3). The YPD targets functional mutant channels to the YPs since ^3^H_2_O uptake assays indicate that platelets with inserted HsAQP1-ΔSD-YPD-HA or DrAqp1aa-ΔSD-YPD-HA were ∼13 and ∼27 times more permeable to water than the control YPs isolated from uninjected oocytes (Fig. 5, D and H).

To test whether the YPD is also effective at targeting aquaglyceroporins to the YPM of frog oocytes, we tested human AQP3 (HsAQP3) and zebrafish Aqp3b (DrAqp3b). The human and zebrafish AQP12 YPDs were introduced at the end of the C terminus of HA-tagged HsAQP3 or DrAqp3b, respectively, while two different tyrosine-based sorting motifs (YSMs) present in the wild-type sequences were abolished by double point mutation (Y19A/Y261A and Y241A/Y261A, for HsAQP3 and DrAqp3b, respectively) (Fig. 5 I). Immunoblotting (Fig. 5 J) and immunostaining (Fig. 5 K) data from oocytes expressing wild-type and mutant constructs confirmed that the unmodified channels were mainly expressed at the oocyte surface, whereas both HsAQP3 and DrAqp3b mutants were targeted to the YPM, where they facilitated glycerol transport (Fig. 5 L).

These experiments show that the YPD is sufficient to rewire the targeting of functional orthodox water and glycerol channels from the plasma membrane to the YPM when expressed in frog oocytes.

### YPM trafficking of mammalian and piscine AQP12 is controlled by the PKC and PKA signaling pathways

**Figure 6.**
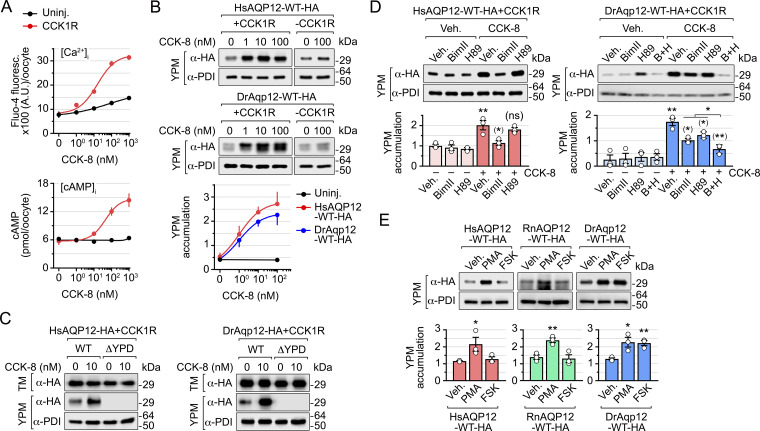
**CCK1R agonist CCK-8 triggers AQP12 YPM targeting via the PKC and PKA signaling pathways in *X. laevis* oocytes. (A)** Dose–response increment in the intracellular levels of Ca^2+^ ([Ca^2+^]_I_; upper panel) and cAMP ([cAMP]_I_; lower panel) (mean ± SEM; *n* = 5 independent experiments) in uninjected oocytes and oocytes expressing the rat CCK1R after treatment with the CCK-8 peptide. **(B)** Anti-HA- and α-PDI–probed western blots of YPM extracts from oocytes injected with HsAQP12-WT-HA (upper blot) or DrAqp12-WT-HA (middle blot), co-expressing or not the CCK1R, and stimulated with increasing doses of CCK-8. The lower panel shows the accumulation of the channels in the YPs normalized to PDI in response to the octapeptide (*n* = 3 independent experiments). **(C)** Representative immunoblots of TM and YPM of oocytes expressing the CCK1R together with HsAQP12-WT-HA or HsAQP12-ΔYPD-HA (left blots), or DrAqp12-WT-HA or DrAqp12-ΔYPD-HA (right blots), and exposed to CCK-8. **(D)** Representative HsAQP12-WT-HA (left) and DrAqp12-WT-HA (right) immunoblots in YPM, and corresponding quantitation normalized to PDI (lower panels), from oocytes expressing the channels and treated with vehicle (0.5% DMSO) or 10 nM of CCK-8, in the presence or absence of 10 μM of PKC and PKA inhibitors BimII and H89, respectively. **(E)** Representative HsAQP12-WT-HA, HA-tagged rat AQP12 (RnAQP12-WT-HA), and DrAqp12-WT-HA immunoblots in YPM, and corresponding quantitation normalized to PDI (lower panels), from oocytes expressing the channels and treated with DMSO alone or 100 nM of PMA or 100 µM FSK. Data in D and E (mean ± SEM, *n* = 3 independent experiments) were statistically analyzed by one-way ANOVA, followed by Tukey’s multiple comparisons test. *P < 0.05; **P < 0.01; with respect to oocytes not treated with CCK-8 (D), or PMA or FSK (E), or with respect to oocytes treated with CCK-8 alone (D in parenthesis), or as indicated in brackets (D). Source data are available for this figure: SourceData F6.

In human pancreatic acinar cells, AQP12 resides in the ER and the ZGs during CCK stimulation of digestive enzyme secretion (Itoh et al., 2005; Ohta et al., 2009), which is mediated through activation of CCK type 1 receptors (CCK1R) (Williams, 2019). This mechanism is likely conserved in fish, since we found that Aqp12 is also localized in the membrane of the ZGs from the acinar cells in the exocrine pancreas of zebrafish (Fig. S2, E–G). We therefore used *X. laevis* oocytes as a surrogate system to investigate CCK-activated signaling pathways controlling the trafficking to YPs of both human and piscine AQP12 channels. To this end, we expressed the rat (*Rattus norvegicus*) CCK1R in oocytes, together with wild-type human AQP12 and zebrafish Aqp12, and subsequently determined the intracellular concentrations of Ca^2+^ ([Ca^2+^]_i_) and cyclic AMP ([cAMP]_i_), as well as the channel targeting to the YPM, in response to sulfated CCK octapeptide (CCK-8). Exposure of oocytes to the peptide generated a dose-dependent increase of [Ca^2+^]_i_ and [cAMP]_i_ according to the binding of the CCK1R to different G proteins (Williams, 2019) (Fig. 6 A). Semiquantitative immunoblot densitometry using PDI for normalization of protein abundance showed that the CCK-8–stimulated increase of the second messengers in the oocytes was associated with the accumulation of HsAQP12 and DrAqp12 in the YPM, whereas in oocytes not expressing the CCK1R, channel trafficking to the YPM did not increase in response to the hormone (Fig. 6 B). AQP12 constructs with an erased YPD did not traffic to the YPM of oocytes expressing the CCK1R either in the presence or in the absence of CCK-8 (Fig. 6 C).

In pancreatic acinar cells, CCK-induced enzyme secretion is linked to the activation of classical protein kinases C (PKCα, PKCβ, and PKCγ) and protein kinase A (PKA) via the increment of [Ca^2+^]_i_ and diacylglycerol (DAG) levels, and of cAMP by adenylyl cyclase activity, respectively (Williams, 2019). Therefore, we tested whether PKC and PKA inhibitors, such as bisindolylmaleimide II (BimII) and N-[2-p-bromocinnamylamino-ethyl]-5-isoquinolinesulfonamide (H89), respectively, could prevent CCK-8–stimulated transport of AQP12 to the YPM of oocytes. The PKC blocker BimII reduced HsAQP12-HA YPM accumulation by ∼90% when induced by CCK-8 with respect to the vehicle-treated controls, while H89 had no effect on channel trafficking (Fig. 6 D). In contrast, each of the inhibitors partially repressed DrAqp12-HA targeting to the YPM (by ∼50% and ∼35% with BimII and H89, respectively), while both compounds together elicited an inhibition of ∼72% (Fig. 6 D).

To confirm these results, oocytes expressing HA-tagged human and zebrafish AQP12 alone were treated with the PKC analog activator phorbol 12-myristate 13-acetate (PMA), which mimics the action of DAG, or the adenylate cyclase activator forskolin (FSK), in the absence of CCK-8. For these experiments, we also expressed RnAQP12-HA to assess whether mammalian channels are regulated in the same manner. Treatment of oocytes with PMA, but not FSK, increased the accumulation of both HsAQP12-HA and RnAQP12-HA in the YPM by approximately twofold with respect to the controls, whereas PMA and FSK enhanced the targeting of DrAqp12-HA to the YPM by ∼1.8 times (Fig. 6 E).

Taken together, these findings suggest that CCK-8–mediated PKC activation is necessary to drive YPM transport of human and rat AQP12, while both PKC and PKA are involved in zebrafish Aqp12 intracellular trafficking.

### PKC and PKA N-terminal phosphorylation of AQP12 regulates channel trafficking

**Figure 7.**
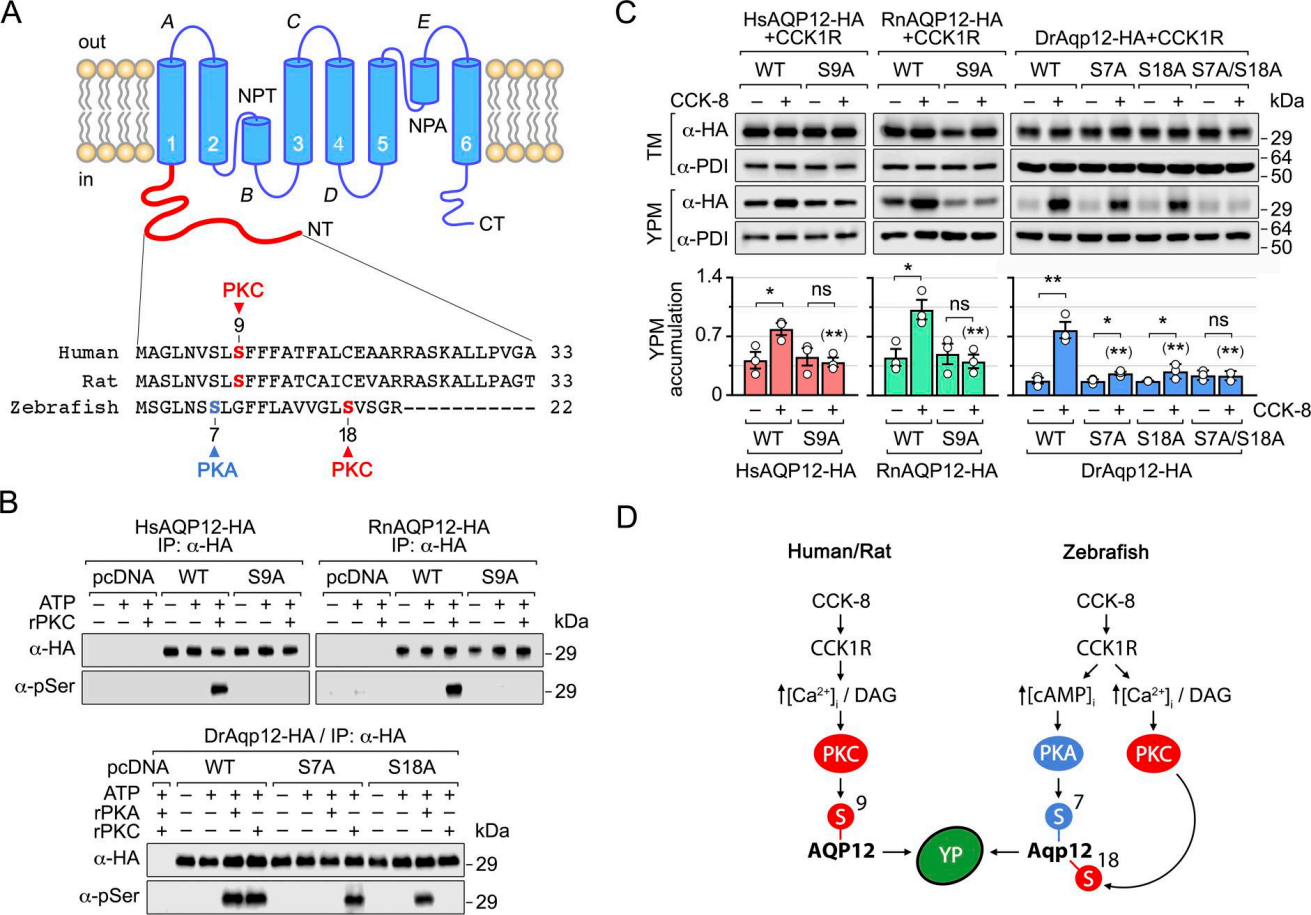
**N-terminal phosphorylation of mammalian and piscine AQP12 by PKC and PKA control YPM channel transport in *X. laevis* oocytes. (A)** Schematic representation of an AQP12 monomer inserted in the plasma membrane showing the putative six transmembrane domains, the five connecting loops (A–E), and the amino acid sequence alignment of the first part of the human, rat, and zebrafish N termini indicating the potential PKC and PKA phosphorylation sites. **(B)** Immunoblots of Ser phosphorylation of immunoprecipitated HsAQP12-HA, RnAQP12-HA, or DrAqp12-HA, and single channel mutants at the PKC and PKA phosphorylation residues, from transiently transfected HEK293T cells by 100 nM of rPKC or rPKA, in the presence or absence of 200 μM ATP. The negative control cells were transfected with an empty pcDNA3 expression vector. Representative blots from two independent experiments are shown. **(C)** Representative immunoblots of HA-tagged human, rat, and zebrafish AQP12 WT and single or double mutants in TM and YPM using α-HA antibody, and corresponding quantitation in YPM normalized to PDI (lower panels), from oocytes expressing the CCK1R and the different constructs and treated with vehicle or 10 nM of CCK-8. Data (mean ± SEM, *n* = 3 independent experiments) were statistically analyzed by one-way ANOVA, followed by Tukey’s multiple comparisons test. *P < 0.05; **P < 0.01; with respect to untreated oocytes, with respect to CCK-treated oocytes (in parenthesis), or as indicated in brackets. **(D)** Proposed model of the CCK1R signaling pathways controlling the trafficking of mammalian and piscine AQP12 to the YPM of *X. laevis* oocytes. TM, total membrane. Source data are available for this figure: SourceData F7.

*In silico* analysis of human, rat, and zebrafish AQP12 proteins revealed the presence of one Ser residue in the N terminus of the human and rat channels (Ser^9^) as a potential phosphorylation recognition site for PKC (Fig. 7 A). In contrast, in zebrafish Aqp12 the N terminus Ser^18^ was identified as a putative PKC-phosphorylated residue, whereas an additional Ser residue (Ser^7^) showed a high score for PKA phosphorylation (Fig. 7 A). To experimentally assess whether PKC and PKA can phosphorylate mammalian and piscine AQP12 channels, we employed *in vitro* phosphorylation assays using *Xenopus sp*. recombinant PKC (rPKC) and human recombinant PKA (rPKA). In these experiments, plasmids expressing HA-tagged HsAQP12, RnAQP12, and DrAqp12, and their corresponding constructs in which all Ser residues in the N termini were independently mutated into Ala, were transiently expressed in human embryonic kidney cells 293T (HEK293T), and further immunoprecipitated using the HA antibody and incubated with rPKC or rPKA in the presence or absence of ATP. Immunoblot analyses of the immunoprecipitates of each construct using phosphorylated Ser-specific antibodies confirmed the ATP-dependent specific phosphorylation of HsAQP12, RnAQP12, and DrAqp12 Ser residues by rPKC, and also by PKA in the case of DrAqp12 (Fig. 7 B). The results also showed that both HsAQP12-S9A-HA and RnAQP12-S9A-HA prevented rPKC Ser phosphorylation, whereas DrAqp12-S7A-HA and DrAqp12-S18A-HA abolished Ser phosphorylation by rPKA and rPKC, respectively (Fig. 7 B). These data therefore indicate that Ser^9^ in HsAQP12 and RnAQP12 is the residue phosphorylated by rPKC, while DrAqp12 Ser^7^ and Ser^18^ are the target sites for rPKA and rPKC, respectively.

Using *X. laevis* oocytes for functional analyses, we next investigated the role of AQP12 phosphorylation in YPM channel trafficking. Oocytes expressing CCK1R combined with wild-type AQP12, or mutant channels at position Ser^9^ for HsAQP12 and RnAQP12 as above, and at Ser^7^ or Ser^18^, or both together, for DrAqp12, were treated with CCK-8, and subsequently, the YPM targeting of the encoded proteins was determined by semiquantitative immunoblotting. The analyses of total protein extracts showed that all mutants were equally expressed compared with the corresponding wild-type channels (Fig. 7 C). However, YPM trafficking in response to CCK-8 was totally inhibited in both HsAQP12-S9A-HA and RnAQP12-S9A-HA constructs with respect to that shown by HsAQP12-HA and RnAQP12-HA (Fig. 7 C). Similarly, hormone-induced YPM transport was partially reduced in both DrAqp12-S7A-HA and DrAqp12-S18A-HA mutants with respect to that elicited by the wild-type channel, whereas trafficking of the DrAqp12-S16C/T24A-HA double mutant was completely abolished (Fig. 7 C). These results confirm that YPM trafficking of mammalian AQP12 in frog oocytes is regulated by N-terminal Ser^9^ phosphorylation by PKC, while Ser^7^ and Ser^18^ in the zebrafish ortholog are, respectively, the target residues of PKA and PKC controlling channel transport to the YPM (Fig. 7 D).

### The YPD and PKC signaling pathway control the targeting of AQP12 to the ZGs in AR42J pancreatic acinar cells

**Figure S4.**
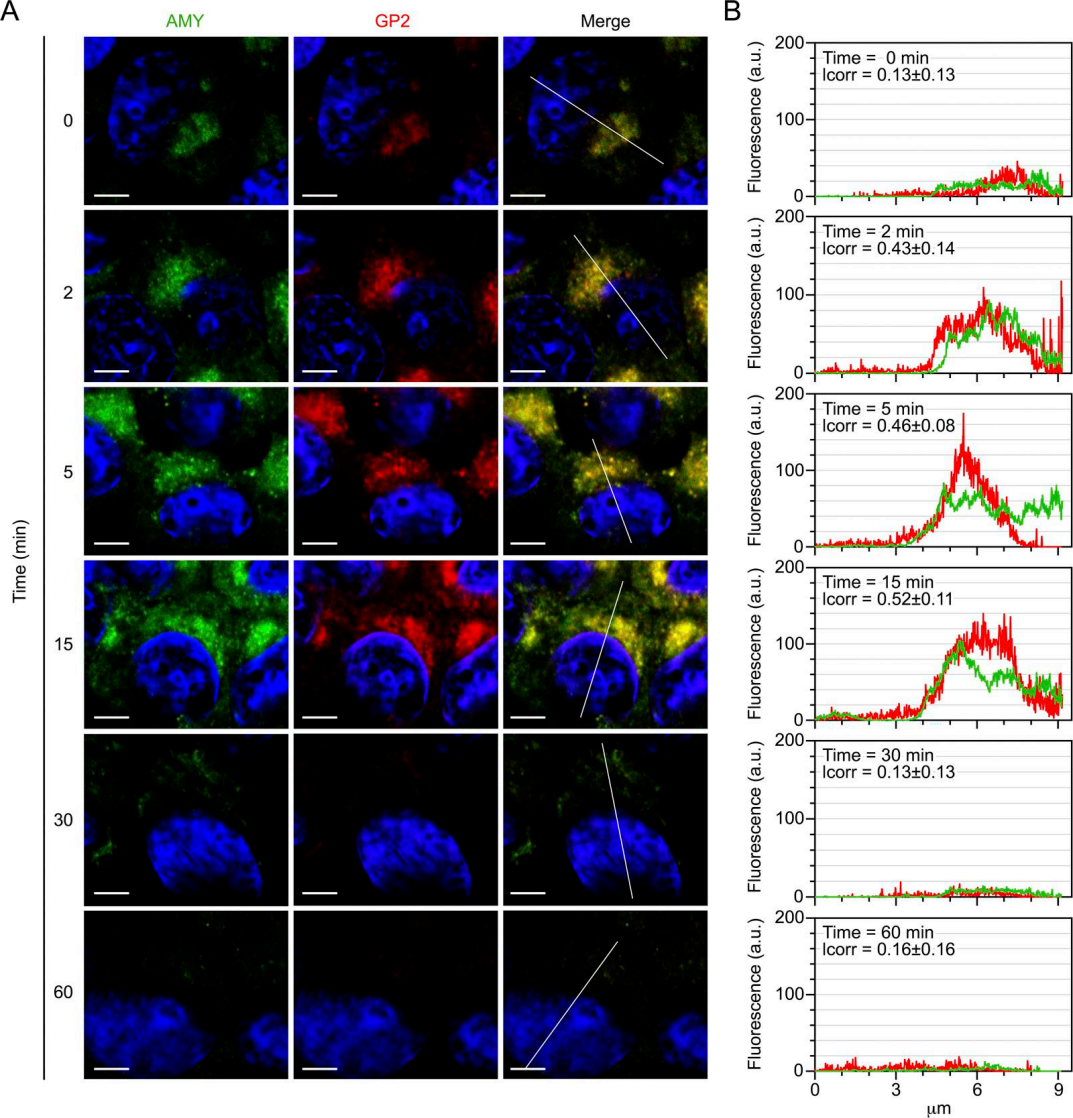
**Time course of ZG activation in AR42J cells induced by CCK-8. (A)** Time course analysis of AMY detection and of the ZG membrane GP2 expression in CCK-8–treated AR42J cells by double immunofluorescence microscopy using AMY- and GP2-specific antibodies (α-AMY and α-GP2, respectively). Scale bars, 2 µm. **(B)** Line-scan profiles (white bars in merged images on the right in A) of fluorescence intensity for the analysis of colocalization of AMY (green curves) and GP2 (red curves). The index of correlation (Icorr) was calculated to measure the amount of colocalization between the AMY and GPS stains in the images (values typically range from 0 to 1, where 0 indicates no colocalization, and 1 denotes complete colocalization).

To investigate whether the YPD and PKC signaling also regulate the trafficking of AQP12 to the intracellular ZGs of pancreatic acinar cells, we employed the AR42J cell line derived from a transplantable tumor of a rat exocrine pancreas. We first evaluated the time course of ZG activation in response to CCK-8 in cells differentiated into amylase (AMY)-secreting exocrine cells with dexamethasone, using immunofluorescence microscopy and antibodies against AMY and the ZG membrane protein glycoprotein 2 (GP2) (Hoops and Rindler, 1991) as molecular markers of the secretory granules (Fig. S4). Confocal laser scanning microscopy analysis showed that maximum colocalization of AMY and GP2 signals occurred within 2 and 15 min after CCK-8 stimulation, suggesting a fast activation of the ZG secretory pathway in response to the secretagogue.

**Figure 8.**
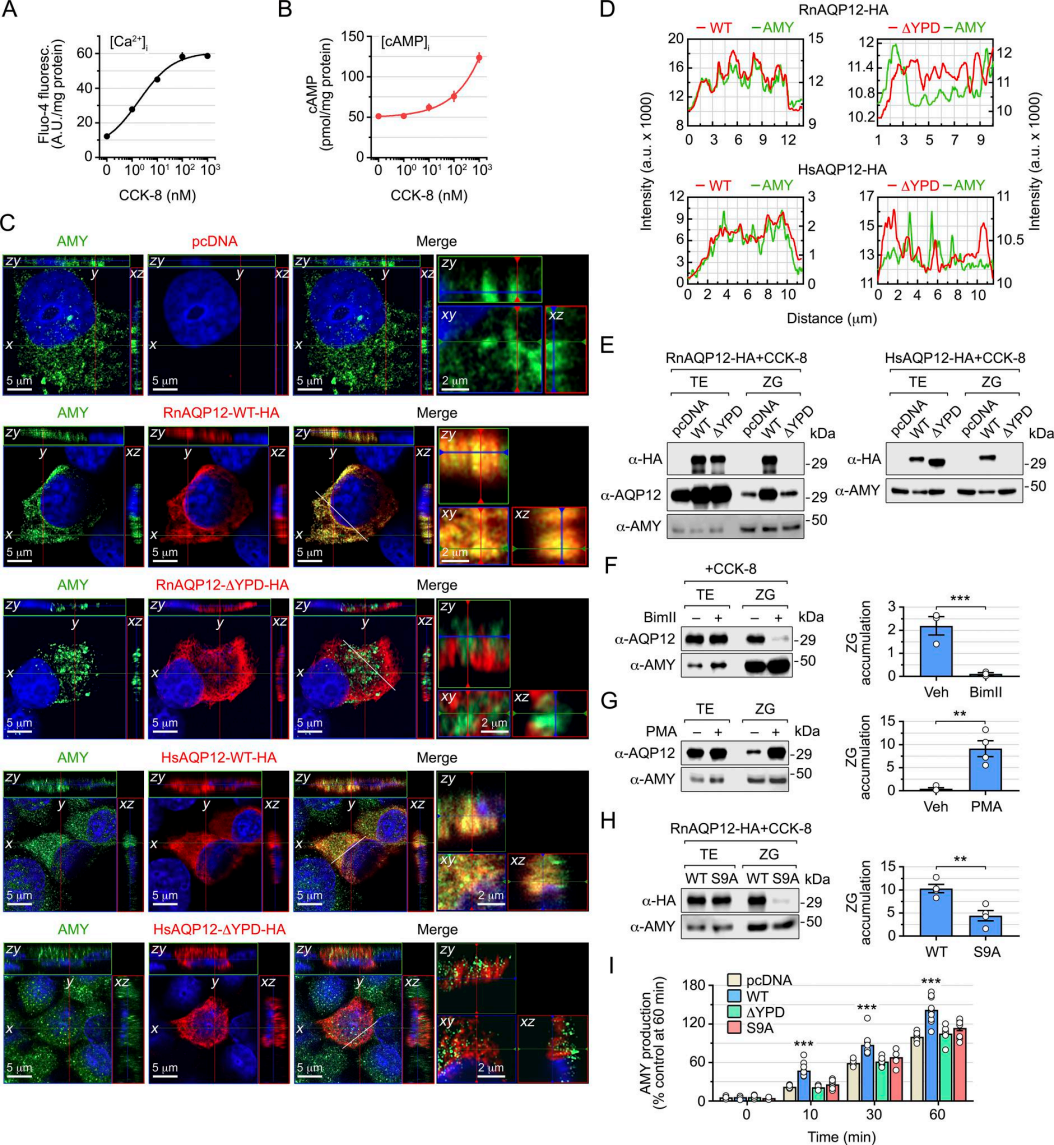
**PKC and YPD control the targeting of rat and human AQP12 to the ZGs in AR42J pancreatic acinar cells. (A and B)** Accumulation of [Ca^2+^]_i_ (A) and [cAMP]_i_ (B) in dexamethasone-treated AR42J cells induced with increasing doses of CCK-8 for 15 min. **(C)** Confocal fluorescent images of dexamethasone-differentiated AR42J cells transiently transfected with an empty pcDNA3 expression vector (pcDNA, control) or a vector carrying the RnAQP12-WT-HA, RnAQP12-ΔYPD-HA, HsAQP12-WT-HA, or HsAQP12-ΔYPD-HA constructs, and incubated with 10 nM CCK-8. The channels are stained with α-HA antibody (red), whereas ZG-specific AMY is detected with a monoclonal antibody (green). The images show the compilation of Z-stack series into orthogonal projections (reconstructed 3D image). **(D)** Line-scan profiles (white bar in the left merge image in C) of fluorescence intensity for the analysis of colocalization of RnAQP12-WT-HA, RnAQP12-ΔYPD-HA, HsAQP12-WT-HA, or HsAQP12-ΔYPD-HA (red curves), and AMY (green curves). **(E)** Western blot of TE or purified ZGs from AR42J cells transfected with the empty pcDNA3 plasmid or expressing the different rat (left) or human (right) constructs as indicated. The different polypeptide constructs were detected using the α-HA antibody or an α-RnAQP12-specific antibody (to detect the endogenous channel), and protein loading was normalized by the α-AMY antibody. **(F and G)** Representative immunoblots of endogenous AQP12 and AMY in TE and ZG extracts from using the α-RnAQP12–specific antibody from nontransfected cells treated with 10 nM CCK-8, in the presence of 10 µM of the PKC inhibitor BimII or the drug vehicle (Veh) (F), or from cells treated only with 100 nM PMA (G). The right panels show the corresponding quantitation of the channel in ZGs normalized to AMY. **(H)** Representative immunoblots of RnAQP12-WT-HA, RnAQP12-S9A-HA mutant, and AMY in TE and ZG extracts using α-HA and α-AMY antibodies, and corresponding quantitation in ZGs normalized to AMY (lower panels), from cells expressing both constructs and treated with 10 nM of CCK-8. In F-H, data (*n* = 3–4 independent experiments) were statistically analyzed by unpaired Student’s *t* test (**P < 0.01; ***P < 0.001; as indicated in brackets). **(I)** Time course of AMY secretion into the culture medium by cells transiently transfected with empty pcDNA3 or vector carrying the different RnAQP12 constructs as indicated and induced by CCK-8. Data (mean ± SEM, *n* = 3 independent experiments) were statistically analyzed by one-way ANOVA at each time point, followed by Tukey’s multiple comparisons test. ***P < 0.01, with respect to empty pcDNA3-transfected cells. TE, total extracts. Source data are available for this figure: SourceData F8.

Treatment of AR42J cells with low doses of CCK-8 induced a robust surge of [Ca^2+^]_i_ but a low increment of the [cAMP]_i_, which only clearly increased in response to supraphysiological doses (>100 nM) of the peptide hormone (Fig. 8 A). Accordingly, AR42J cells were transiently transfected prior to dexamethasone differentiation with empty expression vector (pcDNA3, controls), or carrying wild-type RnAQP12-HA or the RnAQP12-ΔYPD-HA mutant channel, and ZG targeting of the encoded proteins was determined by super-resolution confocal microscopy after 15 min of CCK-8 exposure using α-HA and α-AMY antibodies. Double immunostaining (Fig. 8 B) and analysis of 2D reconstructed fluorescent images (Fig. 8 D) showed the colocalization of RnAQP12-HA and AMY signals, suggesting that the wild-type AQP12 channel is targeted to the ZGs during the activation of secretion (Fig. 7, A and B). In contrast, the signals corresponding to the RnAQP12-ΔYPD-HA construct did not colocalize with those of AMY (Fig. 8, B and D), indicating that this mutant does not traffic to the ZGs in response to CCK-8. Immunoblotting of protein extracts from whole cells and purified ZGs confirmed the CCK-8–induced targeting of RnAQP12-HA to the intracellular secretory granules, whereas the mutant, although equally expressed as the wild type, did not accumulate in these compartments (Fig. 8 E). A parallel blot probed with a RnAQP12-specific antibody to detect both endogenous and exogenous channels also showed an increased amount of AQP12 in the ZGs of cells overexpressing RnAQP12-HA, while this was not observed in cells transfected with the RnAQP12-ΔYPD-HA mutant (Fig. 8 E). These data suggested that exogenously expressed AQP12 constructs did not affect the trafficking of the endogenous channel to the granules. Using AR42J transfectants transiently expressing human AQP12 constructs (HsAQP12-HA or HsAQP12-ΔYPD-HA), we obtained the same results (Fig. 8, C–E), therefore supporting the role of the YPD also as a ZG-targeting signal of AQP12 channels in pancreatic acinar cells.

The observation that low doses of CCK-8 selectively increase [Ca^2+^]_i_, rather than [cAMP]_i_ is consistent with the secondary role of the CCK-cAMP-PKA signaling pathway controlling AMY secretion by pancreatic cells (Williams, 2019). This suggested that only PKC regulates AQP12 ZG transport, as for the frog oocyte YPs. To test this hypothesis, nontransfected cells were treated with CCK-8, in the presence or absence of BimII, or with PMA alone, with ZG targeting of the endogenous AQP12 assessed by semiquantitative immunoblot. The data showed that the PKC inhibitor reduced the amount of AQP12 in the ZG by ∼80% (Fig. 8 F), whereas the kinase activator increased the channel transport to the granules by ∼20-fold (Fig. 8 G). To further investigate the role of PKC driving AQP12 ZG trafficking, cells transfected with wild-type RnAQP12-HA or its S9A mutant were exposed to CCK-8, and the amount of the channels in ZGs, as well as the time course of AMY release into the culture medium, was determined. In these experiments, CCK-8–stimulated enzyme release of cells expressing RnAQP12-ΔYPD-HA was also evaluated. Immunoblot data corroborated that mutation of Ser^9^ reduced CCK-8–triggered AQP12 accumulation in the secretory granules (Fig. 8 H). The control cells elicited a progressive increase in AMY release up to 60 min, whereas those overexpressing RnAQP12-HA showed an enhanced AMY secretion (Fig. 8 I). Such an increment in AMY release with respect to the controls was not observed in cells transfected with RnAQP12-S9A-HA or RnAQP12-ΔYPD-HA, in which the overexpressed channels do not traffic to the ZGs (Fig. 8 I). These data confirm that both the YPD and PKC-mediated N-terminal phosphorylation of Ser^9^ control AQP12 trafficking to ZGs, and suggest that these mechanisms regulate pancreatic acinar enzyme secretion.

### The YPD is a dominant trafficking signal that targets exogenous channels and truncated proteins to ZGs

**Figure 9.**
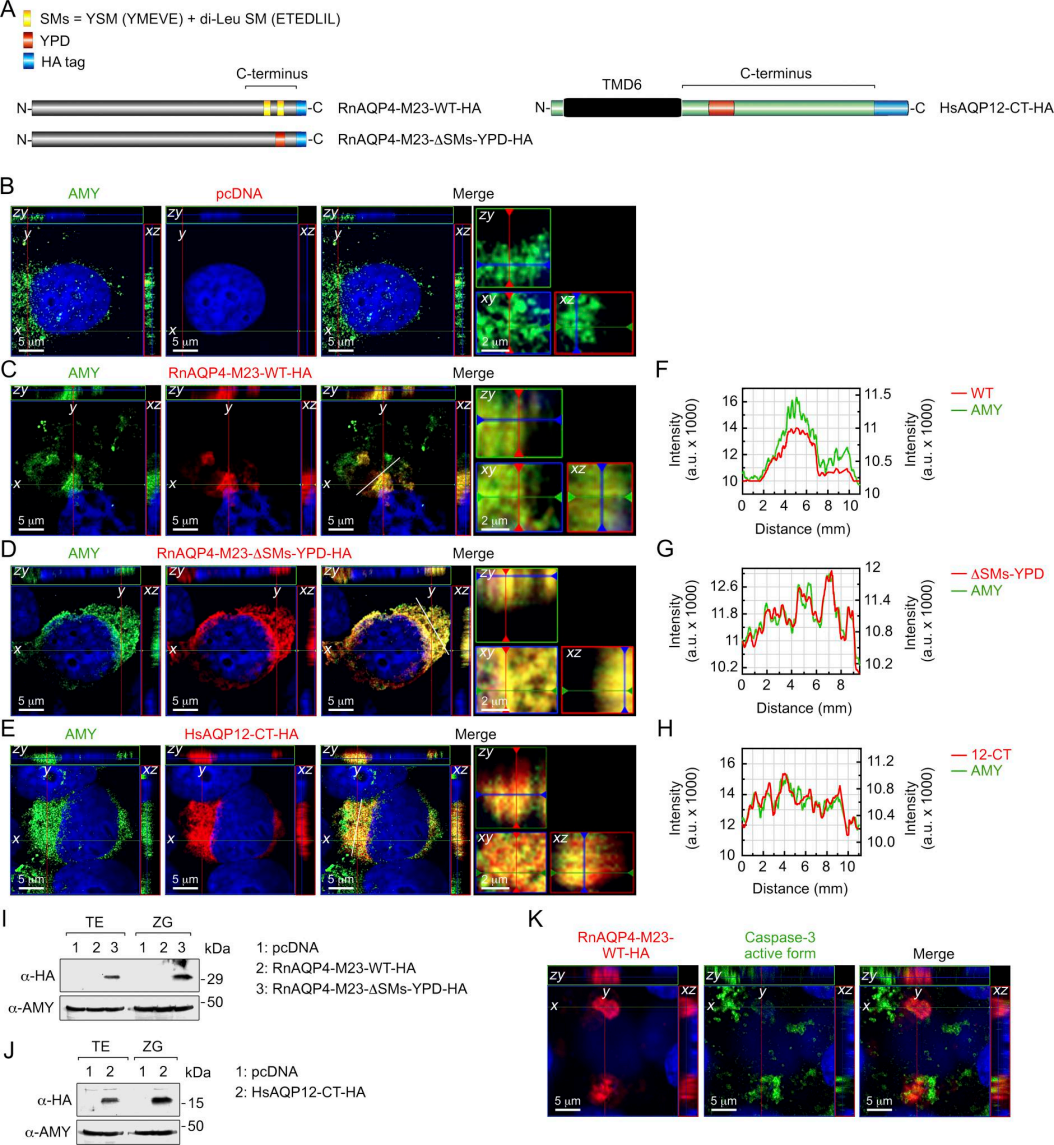
**YPD is a potent trafficking domain that targets exogenous channels and truncated proteins to ZGs in AR42J cells. (A)** Schematic diagrams of HA-tagged WT rat AQP4-M23 isoform (RnAQP4-M23-WT-HA) and mutant in which the two SMs in the C-terminal amino acid sequence, a YSM and diLeu SM, were first erased and the other substituted by the YPD of rat AQP12 (RnAQP4-M23-ΔSMs-YPD-HA). On the right is the diagram of HA-tagged truncated HsAQP12 bearing only the last transmembrane domain (TMD6) and the complete C terminus (HsAQP12-CT-HA). **(B–E)** Confocal fluorescent images of dexamethasone-differentiated AR42J cells transiently transfected with empty pcDNA3 vector (B), or expressing RnAQP4-M23-WT-HA (C), RnAQP4-M23-ΔSMs-YPD-HA (D), or HsAQP12-CT-HA (E), and incubated with CCK-8. The aquaporin channels (red) and AMY (green) are stained with the α-HA and a-AMY antibodies, respectively. The images show the compilation of Z-stack series into orthogonal projections (reconstructed 3D image). **(F–H)** Line-scan profiles (white bar in the merged images, left panels) of fluorescence intensity for the analysis of colocalization of the different constructs (red curves) and AMY (green curves). **(I and J)** Immunoblots of TE and purified ZG from AR42J cells transfected with the pcDNA3 empty plasmid or expressing RnAQP4-M23-WT-HA or mutant construct (I), or HsAQP12-CT-HA (J), as indicated. The different polypeptide constructs were detected using the α-HA, and protein loading was normalized with the α-AMY antibody. Molecular mass markers (kDa) are on the left. **(K)** Confocal fluorescent images of AR42J cells transiently transfected with RnAQP4-M23-WT-HA and exposed to CCK-8 as in B-E stained with α-HA antibody (red) and an antibody against the active form of caspase-3 (green). The images show the compilation of Z-stack series into orthogonal projections (reconstructed 3D image). WT, wild type; TE, total extracts; diLeu SM, dileucine SM. Source data are available for this figure: SourceData F9.

We next evaluated the competence of the AQP12 YPD for ZG targeting of membrane channels that are not endogenously expressed in pancreatic acinar cells, such as the AQP4-M23 isoform which unlike the M1 isoform stabilizes orthogonal arrays of particles in the plasma membrane and facilitates the formation of wider arrays (Furman et al., 2003). For this, we used a HA-tagged RnAQP4-M23 isoform (RnAQP4-M23-HA) and a mutant construct in which the two sorting motifs (SMs) in the C terminus of the channel, a YSM (YMEVE) and a di-Leu motif (ETEDLIL) (Madrid et al., 2001; Wang et al., 2023), were respectively disrupted by a point mutation in Thr^225^ or substituted by the YPD (RnAQP4-M23-ΔSMs-YPD-HA) (Fig. 9 A). The cells were transiently transfected with these constructs and the empty pcDNA3 as a control and stimulated for ZG secretion as before. Immunostaining results showed that wild-type RnAQP4-M23-HA remained intracellular but accumulated in collapsed organelles in the cytoplasm, as observed for the AMY immunostaining (Fig. 9, B and C), while the cells expressing this channel were also positive for the active form of caspase-3 (Fig. 9 K). This construct was not detected in either whole-cell lysates or purified ZG by immunoblotting (Fig. 9 I), which suggests the activation of apoptotic pathways in the cells expressing RnAQP4-M23-HA resulting in the degradation of the protein. In contrast, the mutant RnAQP4-M23-ΔSMs-YPD-HA was dispersed in the cytoplasm, as observed previously for RnAQP12-HA overexpressed in AR42J cells, and colocalized with AMY as indicated by 2D image analysis (Fig. 9, D and G). In addition, immunoblotting data showed that this mutant channel could be detected in both whole-cell and ZG protein extracts (Fig. 9 I). These results therefore suggest that the YPD can rescue exogenously expressed membrane channels from the degradation pathway for targeting to the ZGs.

We finally investigated whether the YPD can transport a truncated AQP12 channel to the ZGs of AR42J cells. Accordingly, an HA-tagged 94–amino acid polypeptide carrying the amino acid sequence of the last transmembrane domain and complete C-terminal sequence of HsAQP12 (HsAQP12-CT-HA) (Fig. 9 A) was synthesized and transiently expressed in the cells. Following differentiation and CCK-8 stimulation, double HA and AMY immunostaining and immunoblot analysis showed that this construct was not degraded but targeted to the ZGs (Fig. 9, E, H, and J). Altogether, these data indicate that the YPD represents a dominant structural unit that is sufficient to specifically drive membrane channels and shorter polypeptides to the ZGs of pancreatic acinar cells.

## Discussion

Our observation that invertebrate and vertebrate AQP12 co-orthologs are intracellularly localized in the YPM when heterologously expressed in frog oocytes prompted us to investigate the broader taxonomic significance of this localization. Using Bayesian inference, we find that the SIPs of plants and green algae and the AQP12-related channels of metazoans from sponges to humans display co-orthologous relationships, a finding that is consistent with previous studies utilizing maximum-likelihood methods (Abascal et al., 2014; Irisarri et al., 2024). Strengthening the notion that the SIPs and AQP12-related channels are likely co-orthologous is the present experimental evidence showing that each of the derived proteins from plants and animals consistently localizes to the intracellular YPM when heterologously expressed in *X. laevis* oocytes. To date, the majority of studies of plant SIPs have reported their intracellular localizations in ER membranes (Ahmed and Chaumont, 2023); however, they have also recently been observed in the pollen-containing anthers, with suggested hydration or peroxiporin roles in germination and pollination (Windari et al., 2021; Miao et al., 2024). Some SIPs have also been observed in the endomembrane system extended from the ER (Miao et al., 2024), but whether this confers a functional role in intracellular vesicle formation and mobilization remains to be ascertained. For AQP12, previous studies have only reported intracellular localizations in the ER and ZGs of mammalian pancreatic cells (Itoh et al., 2005; Gorelick et al., 2006; Koike et al., 2016; Méndez-Giménez 2017). Our data for salmon louse, zebrafish, and Western clawed frog now establish that the observed *ex vivo* YPM localization is recapitulated *in vivo*, while those for rat and zebrafish also show *in vivo* expression in the ZGs (Itoh et al., 2005; Ohta et al., 2009). Since YPs and ZGs are considered to exist in Precambrian organisms, as well as in extant metazoans and bilaterians (Davidson and Erwin, 2009; LaDouceur, 2021), the present findings suggest that the intracellular targeting of AQP12-related channels to intracellular protein storage vesicles is an evolutionarily ancient feature associated with the formation and mobilization of the encapsulated proteins. Within extant Metazoa, we establish that the *in vivo* YP localization is conserved between vertebrate and invertebrate taxa that retain yolked oocytes, while the ZG localization appears to be conserved in vertebrates.

Taking advantage of the observations that AQP12-like channels are targeted to the YPM, we show that isolated frog YPs can be employed as native membrane systems for testing their permeability properties. Using this system, our data indicate that the piscine and mammalian AQP12 channels are polytransporters permeable to water, urea, ammonia, and H_2_O_2_, thus sharing biophysical properties common to both classical and AQP8-type aquaporins and aquaglyceroporins. Construction of chimeric AQP12 channels containing the paralogous AQP1 C termini that direct the heterologously expressed zebrafish and human proteins to the plasma membrane of frog oocytes further corroborated these findings. Permeability to H_2_O_2_ has previously been reported for AQP11, which is suggested to play a role as a peroxiporin for H_2_O_2_ transfer from the ER to the cytosol, or from the mitochondria to the ER, with implications in the development of the polycystic kidney disease (Bestetti et al., 2020; Sorrentino et al., 2022; Speranza et al., 2025), or the prevention of oxidative stress in kidney proximal tubules (Atochina-Vasserman et al., 2013), visceral adipocytes (Frühbeck et al., 2020), and neuronal and glial cells (Amro et al., 2024). The localization of AQP12 in the acinar cells of the exocrine pancreas (Ohta et al., 2009) may indicate a similar role of this channel in redox homeostasis and signaling in pancreatic cells, although direct experimental evidence for this is currently lacking.

**Figure S5.**
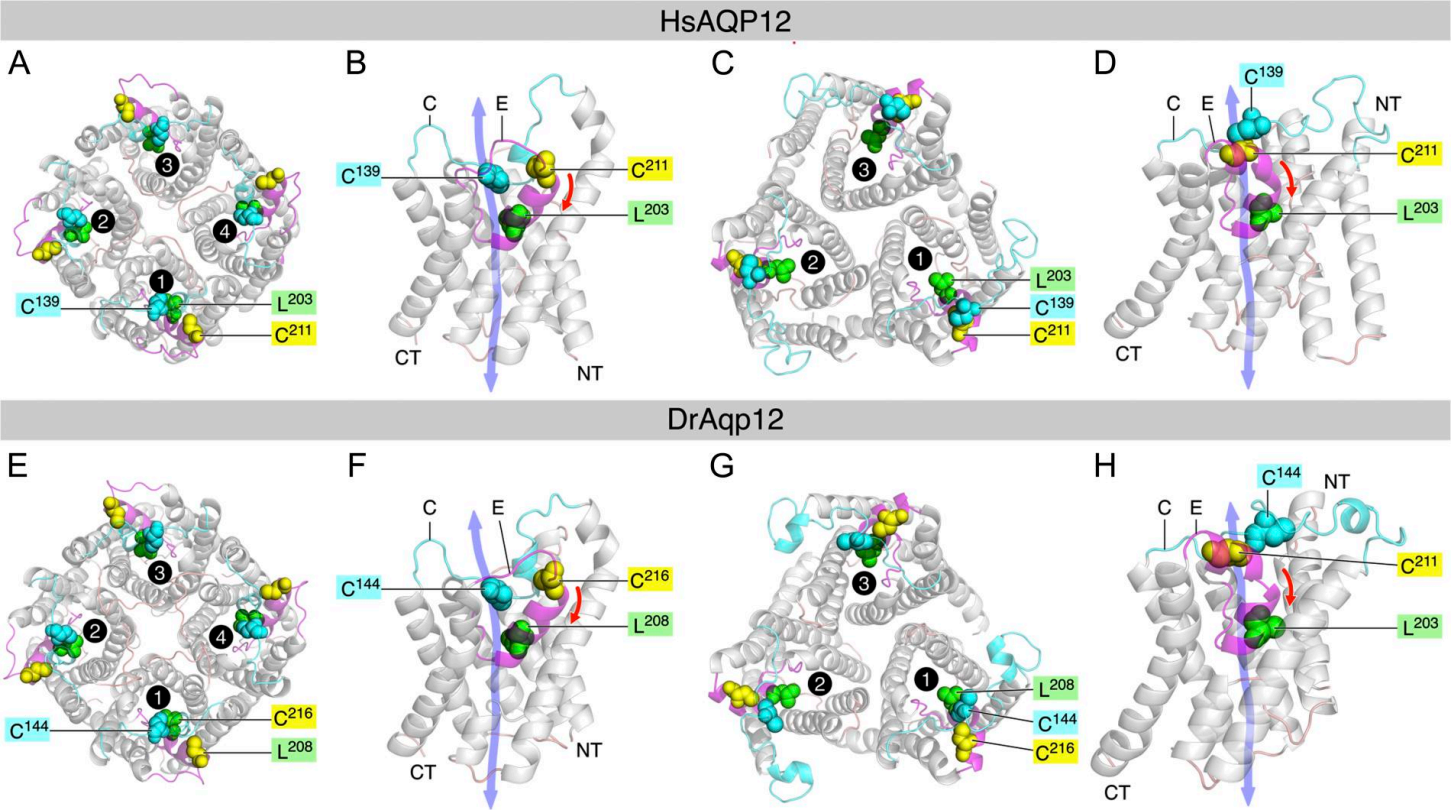
**Quaternary and tertiary *in silico* structures of human and zebrafish AQP12. (A, C,** **E** **, and** **G** **)** Cartoon renders of HsAQP12A and DrAqp12 putative tetramers and trimers showing the monomeric pores 1–4 and 1–3, respectively. The tetramers are modeled on GlpF templates (8Y8W, 8Y8X) and the trimers on an AQP11 template (9VXW). In each render, loop E holds the second hemihelix (magenta) and the ar/R residues Leu^203^ and Leu^208^ (spacefill, green) for HsAQP12 and DrAqp12, respectively. The mercury-sensitive HsAQP12 Cys^211^ and DrAqp12 Cys^216^ residues (spacefill, yellow) are located at the end of the second hemihelix. The inactive HsAQP12 Cys^139^ and DrAqp12 Cys^144^ residues (spacefill, cyan) reside in loop C (cyan). In the trimer, the inactive and mercury-sensitive Cys residues form a disulfide bridge between loop C and loop E, respectively. **(B, D, F, and H)** Membrane planar views of HsAQP12 and DrAqp12 monomers showing the second hemihelix, Cys, and ar/R residues labeled as in A, C, D, and E. The amino and carboxy termini are labeled NT and CT, respectively. Red arrows indicate the potential basis for pore closure through rotation or collapse of the second hemihelix when mercury binds to the sensitive Cys residues.

As shown for most vertebrate orthodox aquaporins (Verkman et al., 2014; Tingaud-Sequeira et al., 2010), our data reveal that the water permeabilities of piscine and mammalian AQP12 channels are sensitive to mercury. The mechanism for mercurial inhibition of orthodox AQP1 results from the binding of Hg^2+^ to the thiol group of Cys^189^, causing a rotation of the residue and a collapse of the aromatic/arginine selectivity filter within each monomeric pore, thus precluding water permeation (Hirano et al., 2010). Here, however, we find that the HsAQP12 Cys^211^ and DrAqp12 Cys^216^ residues are the target sites for mercurial inhibition. Based on *in silico* tetramer and trimer models respectively using an orthodox template and the recently published AQP11 structure (Suzuki et al., 2026), the renders suggest that these mercury-sensitive Cys residues are both likely located at the surface of the E-loop region away from the monomeric pore (Calvanese et al., 2013 and Fig. S5). This location is apparently directly adjacent to the hemihelix bearing the second NPA motif and the Leu^203^ and Leu^208^ selectivity filter residues of HsAQP12 and DrAqp12, respectively. As in AQP11 (Suzuki et al., 2026), the AQP12 trimer models indicate that a disulfide bridge may be formed by Cys^139/144^ in loop C and the mercury-sensitive Cys^211/216^ in loop E. If such is the case, our mutant and mercury-inhibition experiments suggest a dual mechanism for pore occlusion dependent on the presence of this bridge. In the wild-type protein, Hg^2+^ may intercalate the existing disulfide bond (Sperling and Steinberg, 1974), causing a significant structural elongation that may propagate physical distortion through the local architecture, ultimately inducing conformational changes occluding the AQP12 pore. Conversely, when the disulfide bond is absent but the mercury-sensitive Cys^211/216^ remains, such as in the C139A/C144A mutant, direct binding of Hg^2+^ to the free thiol in loop E appears sufficient to block water transport. In this unbridged scenario, it can be envisaged that Hg^2+^ binding causes rotation or collapse of the second hemihelix with either Leu^203/208^ or the NPA junctures blocking the pore in a fashion homologous to the mercury-induced inhibition of AQP1 (Hirano et al., 2010). Importantly, an AQP11 point mutation of Cys^227^ to Ser (the equivalent to Cys^211/216^ in AQP12 channels) has also been reported to produce a phenotype similar to that of AQP11-null mice (Tchekneva et al., 2008). Perturbations in the peripheral loop E sites could therefore translate into changes in protein–protein interactions rather than in pore formation (Yakata et al., 2011). This latter hypothesis is supported by the observed effects of amino acid substitution in the E-loop region on the oligomerization state of orthodox aquaporins (Bron et al., 1999; Owens et al., 2015). Although the molecular structure of AQP12 channels has not yet been elucidated, our data support the notion that in the absence of a Cys^139/144^-Cys^211/216^ disulfide bridge, the mechanism of the mercury-mediated inhibition of water transport may be linked to conformational changes induced by the binding of Hg^2+^ to Cys^211/216^.

We further identified a C-terminal YPD heptapeptide (NLΦYXΖ[+]) that is necessary and sufficient for targeting AQP12 channels to the YPM. Homology analyses reveal that the YPD is highly conserved in the orthologous proteins of 473 jawed vertebrates; however, it is not present in the invertebrate AQP12-related channels or the SIP-related channels of Viridiplantae. This latter observation suggests that other motifs or signaling factors direct the plant and invertebrate channels to the YPs when heterologously expressed in frog oocytes. The YPD nevertheless retains high potency since it targets the vertebrate AQP12 channels to the YPs and ZGs, as well as chimeric constructs of orthodox water channels and aquaglyceroporins and truncated AQP12 channels consisting of only the last transmembrane domain and the C terminus. It is further able to rescue exogenously expressed membrane channels, such as AQP4 that is not endogenously expressed in pancreatic acinar cells, from the degradation pathway by directing them to the ZGs.

In addition to AQP12, the ZGs of pancreatic acinar cells also contain other aquaporins, such as AQP1, which is involved in GTP-mediated water entry and swelling affecting pancreatic ductal fluid and bicarbonate secretion (Cho et al., 2002; Venglovecz et al., 2018), and possibly AQP8 (Rindler et al., 2007), as well as some ion channels and transporters (Thévenod, 2015). AQP1 and AQP8 channels normally reside in the plasma membrane, but in certain cell types, they can be targeted to intracellular secretory vesicles, as well as the mitochondria (Marinelli et al., 2004; Calamita et al., 2005; Chauvigné et al., 2021). However, none of these water and ion channels show a conserved YPD, which suggests that trafficking to the ZGs may also be regulated by different pathways not involving this protein domain.

Employing frog oocytes and cultured AR42J cells, we identified the CCK-8–mediated signal transduction pathways that, in addition to the YPD, control the trafficking of the AQP12 channels to intracellular protein storage vesicles. Our observations indicate that N-terminal phosphorylation of HsAQP12 and RnAQP12 Ser^9^ via PKC is required for channel targeting to the ZGs, which is consistent with the major role of this pathway for CCK-8–induced enzyme secretion in pancreatic acinar cells (Williams 2019). The YPD and the phosphorylation of AQP12 are thus both necessary for channel trafficking to the ZGs. However, a double N-terminal phosphorylation of Ser^7^ and Ser^18^ in DrAqp12, via PKA and PKC, respectively, is required for the transport of the channel to the frog YPs. Since the expression of DrAqp12 is more ubiquitous (Tingaud-Sequeira et al., 2010; Qin et al., 2019) than that of mammalian AQP12, the dual phosphorylation of the zebrafish Aqp12 N terminus may be indicative of the existence of different signaling pathways regulating the intracellular transport of the channel during the formation and processing of the YPs, as well as in other piscine tissues.

The role of AQP12 in pancreatic acinar cells is hypothesized to involve secretory granule swelling as a prerequisite for secretory granule fusion with the plasma membrane with the degree of osmotically driven swelling determining the amount of exocytosis (Sugiya et al., 2008). Such a role would be consistent with the water permeation properties of AQP12 determined here and elsewhere (Bjørkskov et al., 2017). However, pancreatic fluid secretion is not altered in AQP12 knockout mice (Ohta et al., 2009). This may be caused by functional compensation of the other aquaporins expressed in acinar cells (Ishibashi et al., 2021). AQP12-null mice nevertheless develop severe acute pancreatitis after CCK stimulation (Ohta et al., 2009). In this instance, the accumulation of ER H_2_O_2_ in AQP12-null mice lacking the peroxiporin function or the reduction of osmotically driven ZG fusion and exocytosis has been hypothesized as the underlying mechanisms (Ishibashi et al., 2021). Here, the observation that overexpression of wild-type AQP12 in AR42J cells increases AMY secretion, but not when the overexpressed channel is prevented from trafficking to the ZGs, provides support for the role of AQP12 in ZG exocytosis and enzyme secretion. However, whether the role of the channel is to mediate water influx in the ZGs to facilitate vesicle fusion or water efflux to concentrate the digestive enzymes therein (Ishibashi et al., 2021) remains unknown.

In conclusion, we uncover a previously unreported *in vivo* localization of AQP12-related channels in the intracellular YPMs of invertebrates and vertebrates. The demonstration that the plant and metazoan unorthodox channels also traffic to the YPM when heterologously expressed in frog oocytes provides an alternative method for elucidating the biophysical properties of AQP12-like channels including plant SIPs and the signal transduction pathways regulating trafficking of these proteins in native membranes. The native YPM localization further yields opportunities for studying the tertiary, quaternary, and oligomeric structures of the unorthodox channels, which to date has remained challenging due to the absence of expression in plasma membranes. Finally, the discovery of the YPD and demonstration of its potency for targeting and rescuing channel proteins in the YPs and ZGs can advance the development of biotechnologies seeking to deliver cargos of interest to intracellular protein storage vesicles.

## Materials and methods

### *X. laevis* and zebrafish husbandry

Adult *X. laevis* were purchased from the Centre de Ressources Biologiques Xénopes (University of Rennes, France) and maintained at the AQUAB facilities of the Universitat Autònoma de Barcelona (UAB, Spain). Oocytes were collected by surgical laparotomy from anesthetized females following a procedure approved by the Commission for Ethics in Animal and Human Experimentation (CEEAH) from UAB and the Catalan Government (Direcció General de Polítiques Ambientals i Medi Natural; Project no. 10985). The zebrafish were obtained from Pisciber Bio Secure Fishes, S.L., and maintained according to the guidelines provided by Westerfield (2000) and EuFishBioMed/Felasa (Aleström et al., 2020), in compliance with EU 2010/63 guidelines, and approved by the CEEAH from UAB.

### Antibodies and reagents

The commercial and custom-made antibodies employed and dilutions used are described in Table S1. Rabbit polyclonal antisera for human AQP12 and zebrafish Aqp12 were raised against synthetic peptides located in the C-terminal region of the corresponding predicted proteins (REPGRSGVEGPHSS and RLPKGKTNDEKSS, respectively) (Agrisera AB). The antisera were purified by affinity chromatography against the synthetic peptides, and their specificity was confirmed by ELISA and heterologous expression of the protein in *X. laevis* oocytes followed by immunoblotting. The key drugs, enzymes, and other compounds used for the different experiments are listed in Table S2. Other reagents were purchased from Merck unless indicated otherwise.

### DNA constructs and site-directed mutagenesis

The full-length cDNAs used in the present study (Table S3), most of them carrying a HA epitope tag at the end of the C terminus, were cloned or synthesized *in vitro* (GeneArt, Thermo Fisher Scientific), and subsequently subcloned into the pT7Ts (Deen et al., 1994) or pcDNA3 (V79020; Invitrogen) expression vectors. The interchange of complete N or C termini between aquaporin paralogs was carried out by polymerase chain reaction. Point mutations in the amino acid sequences were introduced with QuikChange Lightning Site-Directed Mutagenesis Kit (210518; Agilent Technologies) using the pT7Ts or pcDNA3 plasmids as DNA templates. Oligonucleotides were ordered from Thermo Fisher Scientific. All constructs were verified by DNA Sanger sequencing to confirm that only the desired chimeras or mutations were produced. Truncated HsAQP12 bearing the TMD6 and complete C terminus was synthesized as above.

### Cell cultures

HEK293T and AR42J cells were obtained from American Type Culture Collection (CRL-3216 and CRL-1492, respectively). HEK293T cells were cultured in Dulbecco’s modified Eagle’s medium (10938025; Gibco) supplemented with 2 mM L-glutamine (25030-081; Gibco), 260 U/ml penicillin/streptomycin (15140-122; Gibco), and 10% vol/vol fetal bovine serum (10500064; Gibco) at 37°C in a humidified incubator equilibrated with 5% CO_2_. The AR42J cells were grown in Ham’s F-12K (Kaighn’s) medium (21127022; Gibco), supplemented with penicillin/streptomycin and FSB as above, and also maintained at 37°C under 5% CO_2_. In both cases, cells were transiently transfected at 70% of confluence in a 10-mm diameter petri dish with 5 μg of empty pcDNA3 or vector carrying the selected aquaporin construct using Lipofectamine 3000 (L3000008; Invitrogen) following the manufacturer’s instructions.

### Phylogeny

For phylogenetic analyses, aquaporin sequences were retrieved from open-source protein or transcriptome (NCBI, Ensembl) via blastp or tblastn or assembled from genome databases using either full-length proteins, or exon-deduced peptides as queries. The proteins were then aligned using ClustalW and converted to codon alignments using the corresponding nucleotide sequences with PAL2NAL (Suyama et al., 2006). Phylogenetic analyses were conducted via Mr Bayes v3.2.7a (Ronquist and Huelsenbeck, 2003) with model parameters nucmodel = 4by4, nst = 2, rates = gamma, and aamodel = mixed for the core codon and protein alignments, respectively, after removal of the N and C termini. For each alignment, 10 million Markov chain Monte Carlo generations were run with three heated and one cold chain with resulting posterior distributions examined for convergence and an effective sample size >1,000 using Tracer version 1.7.1 (Rambaut et al., 2018). The majority rule consensus trees were summarized with a burn-in of 25%.

### AQP12 amino acid sequence analysis

*In silico* homology models of human and zebrafish AQP12 were constructed using the SWISS-MODEL automated pipeline (swissmodel.expasy.org) via the ProMod3 engine version 3.6.0 (Waterhouse et al., 2018). The HsAQP12 and DrAqp12 models are based on GlpF (8Y8W, 8Y8X) and AQP11 (9VXW) available templates (https://www.rcsb.org/structure/).

To identify the YPD, the AQP12 amino acid sequences from 473 different gnathostome species were retrieved from open-source protein, transcriptome, and whole genome sequence databases (NCBI, Ensembl, Genome Ark) (www.ncbi.nlm.nih.gov; www.ensembl.org; https://vgp.github.io) via the blastp or tblastn algorithms using the BLOSUM62 matrix and other default parameters (Altschul et al., 1990). The retrieved peptides were aligned via ClustalW and trimmed to the heptapeptide YPD, and the consensus sequences were calculated with MacVector v9.5.2 (MacVector Inc.) using the Zappo scheme of physicochemical properties with a 51% threshold (Waterhouse et al., 2009).

Prediction of kinase-specific phosphorylation residues in AQP12 orthologs was carried out using the NetPhos 3.1 Server (http://www.cbs.dtu.dk/services/NetPhos/) (Blom et al., 2004) and The Eukaryotic Linear Motif resource (http://elm.eu.org/) (Kumar et al., 2024). The SDs and YSMs in the protein sequences of AQP1 orthologs were identified using The Eukaryotic Linear Motif resource software (http://elm.eu.org/) (Gouw et al., 2018).

### Ectopic expression in *X. laevis* oocytes and zebrafish embryos and isolation of YPs

The cRNAs corresponding to the different aquaporin constructs were synthesized *in vitro* from their corresponding cDNAs with T7 RNA polymerase from the *Xba*I-digested pT7Ts vector carrying the *Xenopus* 5′ and 3′ untranslated β-globin sequences. *X. laevis*-stage IV-V oocytes were injected with 15 ng of the cRNAs in 50 nl of water or not injected (controls). After 72 h of incubation in Modified Barth’s Solution (MBS: 88 mM NaCl, 1 mM KCl, 2.4 mM, NaHCO_3_, 0.82 mM MgSO_4_, 0.33 mM Ca(NO_3_)_2_, 0.41 mM CaCl_2_, 10 mM HEPES, and 25 μg/ml gentamycin, pH 7.5), injected and control oocytes (*n* = 30) were homogenized in 80 mM Tris, pH 7.5, and the YPs were isolated by sequential centrifugations following a modified method from Bergeron et al. (2011) (Fig. S1). The effect of PKC or PKA activation on YP channel trafficking was tested by treating oocytes expressing the CCK1R and the constructs with CCK-8 (1–100 nM) for 1 h, in the presence or absence of 10 µM of the BimII or H89 inhibitors, prior to YP extraction. In other experiments, oocytes expressing the constructs alone were preincubated with 100 nM PMA for 30 min, or with 100 μM 3-isobutyl-1-methylxanthine for 1 h, and subsequently with 100 μM FSK for 30 min, before YP isolation. In both types of experiments, control oocytes were treated with 0.1% of the drug vehicle dimethyl sulfoxide (DMSO).

Zebrafish 2-cell embryos were obtained through natural mating and transferred to E2 medium (15 mM NaCl, 0.5 mM KCl, 1 mM MgSO_4_, 0.15 mM KH_2_PO_4_, 0.05 mM Na_2_HPO_4_, 1.0 mM CaCl_2_, 0.7 mM NaHCO_3_) containing 0.1% methylene blue. Embryos were injected with 2 ng of the cRNAs in a volume of 5 nl into the yolk sac, transferred to fresh E2 medium, and grown up to the 20- to 25-somite stage at 28°C. The YPs were then isolated using the same protocol as for *X. laevis* oocytes.

### Immunofluorescence microscopy of *X. laevis* oocytes and YPs

*X. laevis* oocytes expressing HA-tagged aquaporin constructs were fixed for 6 h in 4% paraformaldehyde (PFA) in PBS (137 mM NaCl, 2.7 mM KCl, 10 mM Na_2_HPO_4_, 1.8 mM KH_2_PO_4_, pH 7.5), dehydrated, and mounted in paraffin. Sections (8 μm) were blocked with PBS with 0.05% Tween-20 (PBST) containing 5% normal goat serum and 0.1% BSA for 1 h at room temperature. Isolated YPs were attached to UltraStick/UltraFrost Adhesion slides (63734-01; Electron Microscopy Sciences), fixed in 4% PFA for 15 min, and permeabilized and blocked as above. The slides carrying sections or intact YPs were incubated with primary antibodies (at dilutions indicated in Table S1) overnight at 4°C in a humidified chamber. Slides were subsequently washed three times with PBS and incubated with secondary antibodies for 1 h at room temperature. Slides were washed with PBS and further incubated with WGA for 10 min. The slides were finally mounted with fluoromount aqueous anti-fading medium (F4680; Merck) and examined on a Zeiss Axio Imager Z1/ApoTome fluorescence microscope (Carl Zeiss Corp.). Images were acquired at room temperature with an AxioCam MRm3 camera and ZEN2 (blue edition) software using Zeiss Immersol 518-F immersion oil and EC Plan-Neofluar 40×/1.30 Oil M27 or EC Plan-Neofluar 100×/1.30 Oil M27 objectives (Zeiss), for oocyte sections and YPs, respectively.

### Immunogold TEM

*X. laevis* oocytes were fixed in 4% PFA and 0.1% glutaraldehyde in 0.1 M PBS overnight at 4°C. After washing with 2% PFA in 0.1 M PBS, the samples were dehydrated and embedded into Lowicryl HM20 embedding resin following the progressive lowering temperature method by using a Leica EM AFS freeze substitution device. Preliminary sections of 0.5 μm were carried to localize the YPs, and subsequently, ultrathin transversal sections (70–80 nm) were collected onto 200-mesh copper grids. The grids were blocked in 10 mM M PBS and 50 mM glycine for 6 min at room temperature, and subsequently with 10 mM PBS and 5% bovine serum albumin (BSA) for 15 min. The sections were incubated with α-HA rabbit antibodies (1:50 dilution) overnight at 4°C, washed with PBST (0.25% Tween 20) followed by PBS with 1% BSA, and incubated with the secondary antibodies for 30 min. The grids were washed with 10 mM PBS, refixed with 1% glutaraldehyde in PBS for 10 min, and finally contrasted with 2% aqueous uranyl acetate under dark and with Reynolds’ lead citrate. The sections were observed under a transmission electron microscope Tecnai Spirit 120 kV, equipped with Eagle 4kx4k CCD camera (Thermo Fisher Scientific) at room temperature.

### Water and solute uptake assays

The osmotic water permeability (*P*_f_) of oocytes was determined by measuring the volume changes of uninjected oocytes and aquaporin-expressing oocytes in 10-fold diluted MBS during 20 s at pH 7.5. The permeability of oocytes to radiolabeled water was measured by incubating the oocytes in twofold diluted MBS containing 50 µCi/ml of ^3^H_2_O (ART 0194A; American Radiolabeled Chemicals Inc.) for 10 min. After washing the oocytes in diluted MBS, they were transferred to scintillation vials containing 300 μl of 10% SDS, incubated for 30 min, and mixed with 2.7 ml of liquid scintillation cocktail (LLC6013326; PerkinElmer LLC Ultima Gold). The counts per minute were measured by using a Beckman Coulter scintillation counter. Uptake of glycerol, urea, and methylamine was determined under isotonic conditions in the presence of 20 μCi of [1,2,3-^3^H] glycerol (50 mCi/mmol), [^14^C] urea (58 mCi/mmol), or methylamine [^14^C] hydrochloride (55 mCi/mmol) (ART 0413, ARC 0150A, and ARC 0167; American Radiolabeled Chemicals Inc., respectively), and cold glycerol, urea, or methylamine at 1 mM final concentration. For H_2_O_2_ uptake assays, oocytes were incubated with isotonic MBS plus 0.5% DMSO and 200 μM of CM-H_2_DCFDA (C6827; Life Technologies Corp.) for 1 h, and fluorescence was measured at excitation and emission wavelengths of 495 and 525 nm, respectively, using Spark Multimode Microplate Reader (Tecan Group Ltd.).

The permeability of isolated YPs to radiolabeled water and solutes was determined by incubating the YPs with 10 µCi/ml of ^3^H_2_O in twofold diluted 80 mM Tris-HCl in ultra-pure water during 10 min, centrifuged at 100 × *g* for 1 min, and resuspended in 100 μl 40 mM Tris-HCl. Samples were transferred to scintillation vials and counted as above. In some experiments, ^3^H_2_O permeability of YPs was determined in the presence or absence of 100 µM HgCl_2_ with or without 5 mM β-mercaptoethanol. For glycerol, urea, and methylamine, YPs were incubated with 10 µCi/ml of the radiolabeled compounds in 80 mM Tris-HCl containing cold glycerol, urea, or methylamine at 1 mM final concentration for 10 min. After centrifugation, YP pellets were resuspended in 100 μl 80 mM Tris-HCl supplemented with 1 mM of the cold solutes, transferred to scintillation vials, and counted. For H_2_O_2_ uptake, YPs were incubated with isotonic MBS with 0.5% DMSO and 200 μM of CM-H_2_DCFDA for 1 h, and after 100 × *g* centrifugation, the YPs were washed in MBS+0.5% DMSO, and pelleted again at 100 × *g*. The YP pellet was resuspended in MBS+0.5% DMSO containing 100 µM H_2_O_2_ for 10 min, and fluorescence was subsequently measured at excitation and emission wavelengths of 495 and 525 nm, respectively, using the multimode microplate reader. In all cases, the uptake of the radiolabeled compounds and H_2_O_2_ was normalized to the amount of protein in the YPs determined by using the Bradford protein assay (Bio-Rad Laboratories).

### Determination of [cAMP]_i_ and [Ca^2+^]_i_ and AMY release

The [cAMP]_i_ was determined using the Cyclic AMP ELISA kit (581001; Cayman). Five replicates of CCK1R-expressing *X. laevis* oocytes (*n* = 10 each) were treated with CCK-8 (0, 1, 10, 100, 1000 nM) for 15 min, homogenized in 125 μl of 0.1M HCl, and subsequently centrifuged at 1,000 × *g* for 10 min at room temperature. The supernatant was transferred to a new tube and mixed with the same volume of assay buffer to neutralize the acid. Then, 50 μl of the sample was used for the assay, which was performed following the manufacturer’s instructions. The production of cAMP was measured spectrophotometrically at a wavelength of 450 nm using the same multimode microplate reader as above. To determine the [cAMP]_i_ in AR42J cells, the cells grown in 12-well plates and previously differentiated with dexamethasone were treated with the same doses of CCK-8 for 15 min, lysed in 250 μl of 0.1M HCl, and processed as described for *X. laevis* oocytes. Each group was assayed in triplicate, and protein concentration was measured with the Bradford method.

The [Ca^2+^]_i_ in *X. laevis* oocytes was measured by loading 12 oocytes from each group with 5 µM of the fluorescent calcium probe Fluo-4 AM (F14201; Life Technologies), previously mixed with 20% Pluronic F-127 dissolved in DMSO before use, for 1 h. Oocytes were washed in MBS and incubated with the different doses of CCK-8 for 15 min, and subsequently, each oocyte was deposited in one well of a black 96-well microplate (Nunc F96 MicroWell Black and White Polystyrene Plate; Thermo Fisher Scientific Inc.). The fluorescence intensity was measured fluorometrically at excitation and emission wavelengths of 345 and 494 nm, respectively, and the background signal from oocytes not loaded with Fluo-4 AM was subtracted. The same protocol was employed to measure [Ca^2+^]_i_ in AR42J cells, which were grown as previously described for cAMP determination.

The amount of secreted AMY into the culture media by AR42J cells was carried out using EnzChek Ultra AMY Assay Kit (E33651; Molecular Probes) following the manufacturer’s instructions. The medium (150 μl) was collected at time 0 and 10, 30, and 60 min following CCK-8 treatment. An aliquot of the medium was used for protein determination using the Bradford method. The AMY fluorometric assay was performed in duplicate using 50 μl of the medium and read using a Spark Multimode Microplate Reader, at an excitation wavelength of 485 nm and an emission wavelength of 530 nm. Four independent experiments were carried out, and the secretion of AMY was calculated as the percentage with respect to the pcDNA3-transfected cells (controls) at 60 min after CCK-8 induction. For each data point, background fluorescence was corrected by subtracting the value from the medium without enzyme.

### Confocal microscopy analysis of ZG activation and aquaporin ZG trafficking in cultured AR42J cells

The time course of ZG activation was evaluated by incubating the cells attached to 15-mm-diameter round coverslips (72196-15; Electron Microscopy Sciences) with 100 nM dexamethasone during 48 h to induce their differentiation into the exocrine secreting phenotype. Initiation of secretion of ZGs was subsequently induced by stimulation of the cells with 10 nM CCK-8. At different times after CCK-8 addition up to 60 min, cells were fixed in methanol during 6 min, and subsequently in acetone for 30 s, at −20°C. After two washes in PBS, cells were permeabilized with PBS+0.1% Triton X-100 for 10 min and blocked in 5% goat serum and 0.1% BSA in PBST (0.1% Tween-20) for 1 h before incubation with a monoclonal antibody anti-α-AMY 1A and a polyclonal antibody against GP2 (Table S1) overnight at 4°C. After washing in PBS, cells were exposed to secondary antibodies for 1 h at room temperature. The nuclei were counterstained with 4′,6-diamidino-2-phenylindole at 1:5,000 dilution for 3 min, and coverslips were mounted with Fluoromount aqueous anti-fading medium as above. Images were acquired on a Zeiss LSM 700 confocal microscope equipped with Examiner.D1 scanner and Axio Imager 7.2 detector, AxioCam MRm camera, and Zen 2010 B SP1 software. Imaging was carried out at room temperature using a Plan-Apochromat 63×/1,40 Oil DIC M27 objective (Zeiss) and 20 z-stack scan mode. The digital scan zoom was fixed at 2× (124× final). To quantify the amount of colocalization between the AMY and GP2 fluorescent stains in both images during the time course, we calculated Pearson’s correlation coefficient (or index of correlation) using ImageJ software with the Colocalization Colormap plugin (Jaskolski et al., 2005).

To investigate aquaporin trafficking to ZGs, the AR42J cells were transiently transfected with HA-tagged aquaporin constructs before dexamethasone differentiation and treated with CCK-8 for 15 min as above. Immunostaining was carried out with the α-AMY 1A monoclonal antibody and the HA polyclonal antibody. Super-resolution images were acquired at room temperature on a Zeiss LSM 980-PicoQuant super-resolution confocal microscope equipped with a GaAsp-Airyscan 2-PMT detector, AxioCam 506 mono camera, and Zen 3.5 lite software using a Plan-Apochromat 63×/1.40 Oil DIC M27 objective (Zeiss). The scan mode of 30 z-stacks was bidirectional, and the digital scan zoom was fixed at 6× (378× final). The Fast Airyscan Sheppard Sum SR-4Y deconvolution mode was applied for all images that were further analyzed using the ZEN 3.5 software. The “Ortho” function allowed the visualization of the signals in red, green, and blue in the x and y axis of the 2D picture in a particular z-stack, while the yellow color reflects colocalization of both proteins. The “Profile” function was used to plot fluorescence intensity data for both green and red channels, and superposition of the graphs reflects colocalization. For each picture, a numerical zoom was created on a region of interest (ROI) where the ZGs were localized based on α-AMY staining. The ROI corresponds to a 2,000× magnification, and the ortho and profile functions were also applied to the ROI.

### Protein extraction and immunoblot analysis

*X*. *laevis* oocytes and zebrafish embryos (*n* = 10) were homogenized in HbA buffer (20 mM Tris, pH 7.4, 5 mM MgCl_2_, 5 mM NaH_2_PO_4_, 1 mM EDTA, 80 mM sucrose, and EDTA-free protease inhibitor cocktail), and the total and plasma membrane fractions were isolated by sequential centrifugations (Ferré et al., 2023). For the isolation of the YPM, YPs isolated from oocytes and embryos were homogenized by pipetting with 200-μl pipette tips in HbA buffer, followed by vortexing during 1 min. The homogenate was centrifuged at 200 × *g* for 5 min and the supernatant placed on ice. The pellet was homogenized again in 200 μl HbA buffer and centrifuged at 200 × *g* for 5 min, and the resulting supernatant was pooled with that of the first extraction. The YPM extracts were pelleted at 20,000 × *g* for 20 min and resuspended in 1 × Laemmli sample buffer.

The isolation of ZG from cultured AR42J cells was performed following the protocol from Chen and Andrews (2008). Cells grown on 10-cm petri dishes were resuspended in 3.2 ml of a homogenization buffer containing 0.25 M sucrose, 25 mM 2-morpholinoethanesulfonic acid at pH 6.0, 0.1 mM MgSO_4_, 2 mM EGTA, and 0.1 mM phenylmethylsulfonyl fluoride, and homogenized in a Teflon-glass homogenizer on ice. A 100-μl aliquot of the homogenate (total extract) was mixed with 100 μl of 2 × Laemmli sample buffer containing 10% β-mercaptoethanol, heated at 95°C for 10 min, deep-frozen in liquid nitrogen, and stored at −80°C until further analysis by western blot. The rest of the extract was separated into three 1-ml aliquots, transferred to 1.5-ml Eppendorf tubes, and centrifuged at 200 × *g* for 10 min at 4°C. The supernatants were removed and the pellets gently resuspended with 4 ml of 50% Percoll, transferred to 4-ml centrifuge tubes (3127-LG03 84-13-2-5; Thermo Fisher Scientific), and centrifuged at 67,000 × *g* for 20 min at 4°C in a Sorvall Discovery M150 SE ultracentrifuge (Thermo Fisher Scientific) using a S52ST-252 swinging bucket rotor. Bottom white bands were collected and washed with 1.5 ml homogenization buffer and centrifuged at 1,500 × *g* or 10 min 4°C to remove the Percoll. The pellet containing purified ZGs was resuspended in 100 μl of 1 × Laemmli sample buffer and treated as above.

Laemmli-mixed protein samples were heated at 95°C for 10 min and subjected to 12% sodium dodecyl sulfate–polyacrylamide gel electrophoresis, and blotted onto Immun-Blot nitrocellulose 0.2 μm membranes (Bio-Rad Laboratories). Membranes were blocked with 5% nonfat dry milk in TBST (20 mM Tris, 140 mM NaCl, 0.1% Tween 20, pH 8) for 1 h at room temperature, and subsequently incubated overnight at 4°C with the selected primary antibody (Table S1) diluted in TBST with 5% milk. Bound antibodies were detected with horseradish peroxidase–coupled secondary antibodies (Table S1) diluted as above and applied for 1 h at room temperature. Immunoreactive bands were revealed by WesternSure PREMIUM Chemiluminescent Substrate (LI-COR) using a digital scanner (C-DiGit Blot Scanner, LI-COR). For semiquantitation of protein abundance, the intensity of the corresponding immunoreactive bands was determined by densitometry using the software Image Studio 5.2 (LI-COR) and normalized to that of either PDI (for *X. laevis* oocyte or zebrafish embryo YPs) or α-AMY (for AR42J cells and ZGs).

### *In vitro* phosphorylation assays

HEK293T cells were transfected with empty pcDNA3 or pcDNA3 plasmids containing different HA-tagged AQP12 constructs. After 48 h, the cells were lysed in a radioimmunoprecipitation assay buffer (150 mM NaCl, 50 mM Tris, pH 8.0, 1.0% Triton X-100, 0.5% sodium deoxycholate, 0.1% SDS, supplemented with 1 mM NaF, 1 mM Na_3_VO_4_, and protease inhibitors). The extracts were mixed with activated G protein beads (Pure Proteome Protein G Magnetic Beads, LSKMAGG) coupled to rabbit α-HA antibody following the manufacturer’s instructions, and incubated overnight at 4°C under constant agitation. The beads were further washed three times with PBST, eluted in 50 μl of elution buffer (0.1% trifluoroacetic acid in water, pH 2.0), and neutralized with 10 μl Tris 0.1M, pH 8.5. The eluates were mixed on ice with 60 μl of 2 × kinase assay buffer (50 mM MOPS, pH 7.2, 25 mM β-glycerophosphate, 50 mM MgCl_2_, 10 mM EGTA, 4 mM EDTA, 0.2 mM Na_3_VO_4_, and 0.5 mM DTT), containing 100 ng/ml BSA with or without 200 μM ATP and 100 nM of rPKC or PKA (Table S2). The mixture was incubated at 30°C for 30 min and the reaction terminated by adding 4 × Laemmli sample buffer containing 20% β-mercaptoethanol and heating at 95°C for 10 min. Samples were subsequently processed for immunoblotting as described above.

### Statistical analysis

Plots were generated, and statistical analyses were performed using GraphPad Prism 10. Error bars represent the SEM. Data were tested for both normal distribution and homogeneity of variance by the Kolmogorov–Smirnov and Bartlett’s tests, respectively. Statistical comparisons were made by two-tailed unpaired Student’s *t* test or one-way analysis of variance (ANOVA), followed by Tukey’s multiple comparisons test. The number of independent experiments and biological replicates performed for all experiments, P values, and the specific statistical test used are indicated in the figure legends.

### Online supplemental material

Fig. S1 shows the procedure for the isolation of intact YPs from *X. laevis* oocytes. Fig. S2 shows the localization of endogenous and exogenous AQP12-like channels in yolk-retaining oocytes and embryos from invertebrates and vertebrates, and ZG of zebrafish pancreatic acinar cells. Fig. S3 shows that the HA epitope in the C termini of HsAQP12, DrAqp12, or HaAQP1 and DrAqp1aa mutants does not affect the trafficking of the channels to the YPM. Fig. S4 shows the time course of ZG activation in AR42J cells induced by CCK-8. Fig. S5 shows the *in silico* structural models for HsAQP12 and DrAqp12 and a putative mechanism for mercury inhibition. Table S1 summarizes the technical specifications of all primary and secondary antibodies used in this study. Table S2 indicates the key reagents employed for some of the experiments. Table S3 lists the GenBank accession numbers of the aquaporin- and CCK1R-coding cDNAs used in the study.

## Supplementary Material

Review History

Table S1 shows antibodies used in this study.

Table S2 shows key reagents employed.

Table S3 shows aquaporin- and CCK1R-coding cDNAs used in the study.

SourceData F1 is the source file for Fig. 1.

SourceData F2 is the source file for Fig. 2.

SourceData F3 is the source file for Fig. 3.

SourceData F4 is the source file for Fig. 4.

SourceData F5 is the source file for Fig. 5.

SourceData F6 is the source file for Fig. 6.

SourceData F7 is the source file for Fig. 7.

SourceData F8 is the source file for Fig. 8.

SourceData F9 is the source file for Fig. 9.

SourceData FS1 is the source file for Fig. S1.

SourceData FS2 is the source file for Fig. S2.

SourceData FS3 is the source file for Fig. S3.

## Data Availability

Data are available in the article itself and its supplementary materials. The amino acid alignments and phylogenetic trees are available upon request.
