# Supplementary material for: A pan-vertebrate signaling motif controls the molecular function of intracellular AQP12
Source: J Cell Biol. 2026 Jul 2;225(8):e202512040. doi: 10.1083/jcb.202512040 (PMC13344155; doi:10.1083/jcb.202512040)
Supplement: Table S1 — shows antibodies used in this study. [file jcb_202512040_tables1.docx]

**Supplementary Table S1. Antibodies used in this study**

| **Antibody** | **Source** | **Cat. No./Reference** | **Dilution^a^** |
| --- | --- | --- | --- |
| HA Tag | Thermo Fisher Scientific | PA1-985 | 1:2000 (WB)/1:500 (IF) |
| Vitellogenin | Biosense laboratories | V01407101 | 1:500 (WB)/1:100 (IF) |
| PDI | Merck | P7496 | 1:2000 (WB)/1:500 (IF) |
| Louse Aqp12L2 | Custom-made | Stavang et al. (2015) | 1:500 (WB)/1:400 (IF) |
| Louse PripL | Custom-made | Stavang et al. (2015) | -/1:400 (IF) |
| Human AQP12 | Custom-made | This study | 1:500 (WB)/1:250 (IF) |
| Zebrafish Aqp12 | Custom-made | This study | 1:500 (WB)/1:250 (IF) |
| Rat AQP12 | GeneTex | GTX47925 | 1:500 (WB)/1:250 (IF) |
| Human AQP12B | Invitrogen | PA5-48287 | 1:200 (WB)/- |
| Human AQP1 | Merck | AB2219 | 1:500 (WB)/1:250 (IF) |
| Seabream Aqp1aa | Custom-made | Raldúa et al. (2008) | 1:500 (WB)/1:250 (IF) |
| α-AMY1A | Merck | SAB4200673 | 1:1000 (WB)/1:200 (IF) |
| V-ATPase | GenScript | A00938-40 | -/1:200 (IF) |
| GP2 | Invitrogen | PA5-42593 | 1:1000 (WB)/1:250 (IF) |
| Caspase-3 active form | Merck | MAB10753 | 1:100 (IF) |
| Phosphoserine (5B12) | Signalway Antibody | 12828 | 1:1000 (WB)/- |
| Rabbit IgG HRP | BioRad | 172-1019 | 1:5000 (WB)/- |
| Mouse IgG HRP | BioRad | 172-1011 | 1:5000 (WB)/- |
| Rabbit IgG Cy3 | Merck | C2306 | -/1:1000 (IF) |
| Mouse IgG Alexa Fluor 488 | Invitrogen | A11001 | -/1:1000 (IF) |

^a^ WB, Western blot; IF, immunofluorescence microscopy.
