# Supplementary material for: A pan-vertebrate signaling motif controls the molecular function of intracellular AQP12
Source: J Cell Biol. 2026 Jul 2;225(8):e202512040. doi: 10.1083/jcb.202512040 (PMC13344155; doi:10.1083/jcb.202512040)
Supplement: Table S2 — shows key reagents employed. [file jcb_202512040_tables2.docx]

**Supplementary Table S2. Key reagents employed**

| **Reagent** | **Abbreviation** | **Source** | **Cat. No.** |
| --- | --- | --- | --- |
| Wheat germ agglutinin Alexa Fluor 647 conjugate | WGA | Life Technologies | W32466 |
| Fluo-4 Esther AM | Fluo-4-AM | Life Technologies | F14201 |
| H89 dihydrochloride hydrate | H89 | Merck | B1427 |
| Bisindolylmaleimide II | BimII | Santa Cruz Biotechnology | sc-221366 |
| Phorbol 12-myristate 13-acetate | PMA | Merck | P8139 |
| Forskolin | FSK | Merck | F6886 |
| Recombinant PKC-α | rPKC-α | Merck | SRP5251 |
| PKA Protein, Recombinant | rPKA | Merck | 14-440-M |
| Cholecystokinin fragment 26-33 amide | CCK-8 | Merck | C2175 |
| Radiolabeled water | ^3^H_2_O | American Radiolabeled Chemicals | ART 0194A |
| Radiolabeled glycerol | [1,2,3-^3^H] glycerol | American Radiolabeled Chemicals | ART 0218A |
| Radiolabeled urea | [^14^C] Urea | American Radiolabeled Chemicals | ARC 0150 |
| Radiolabeled methylamine | [^14^C] Methylamine | American Radiolabeled Chemicals | ARC 0167 |
