# Supplementary material for: A pan-vertebrate signaling motif controls the molecular function of intracellular AQP12
Source: J Cell Biol. 2026 Jul 2;225(8):e202512040. doi: 10.1083/jcb.202512040 (PMC13344155; doi:10.1083/jcb.202512040)
Supplement: Table S3 — shows aquaporin- and CCK1R-coding cDNAs used in the study. [file jcb_202512040_tables3.docx]

**Supplementary Table S3. Aquaporin- and CCK1R-coding cDNAs used in the study**

| **GenBank acc.no** | **Species** | **Protein** | **Tag^1^** |
| --- | --- | --- | --- |
| NM_198998.2 | *Homo sapiens* | AQP12A | HA/- |
| NM_198098.4 | *Homo sapiens* | AQP1 | HA/- |
| NM_004925.5 | *Homo sapiens* | AQP3 | HA |
| NM_001109009.1 | *Rattus norvegicus* | AQP12 | HA |
| NM_004028.5 | *Rattus norvegicus* | AQP4-M23 | HA |
| NM_012688.3 | *Rattus norvegicus* | CCK1R | - |
| NP_001039327.1 | *Danio rerio* | Aqp12 | HA/- |
| NM_207059.1 | *Danio rerio* | Aqp1aa | HA/- |
| EU341832.1 | *Danio rerio* | Aqp3b | HA |
| KR005666.1 | *Lepeophtheirus salmonis* | Aqp12L2 | - |
| NM_001264219.3 | *Caenorhabditis elegans* | Aqp12 (Aqp-9)^2^ | HA |
| XM_001647472.1 | *Nematostella vectensis* | Aqp12 | HA |
| NM_111280.5 | *Arabidopsis thaliana* | SIP1A | HA |

^1^ The HA epitome tag was introduced at the end of the C-terminus. Some constructs were also untagged indicated as ‘-‘.

^2^ Formerly termed Aqp-9.
