# Supplementary figures and images for: A pan-vertebrate signaling motif controls the molecular function of intracellular AQP12
Source: J Cell Biol. 2026 Jul 2;225(8):e202512040. doi: 10.1083/jcb.202512040 (PMC13344155; doi:10.1083/jcb.202512040)

Figure 1C

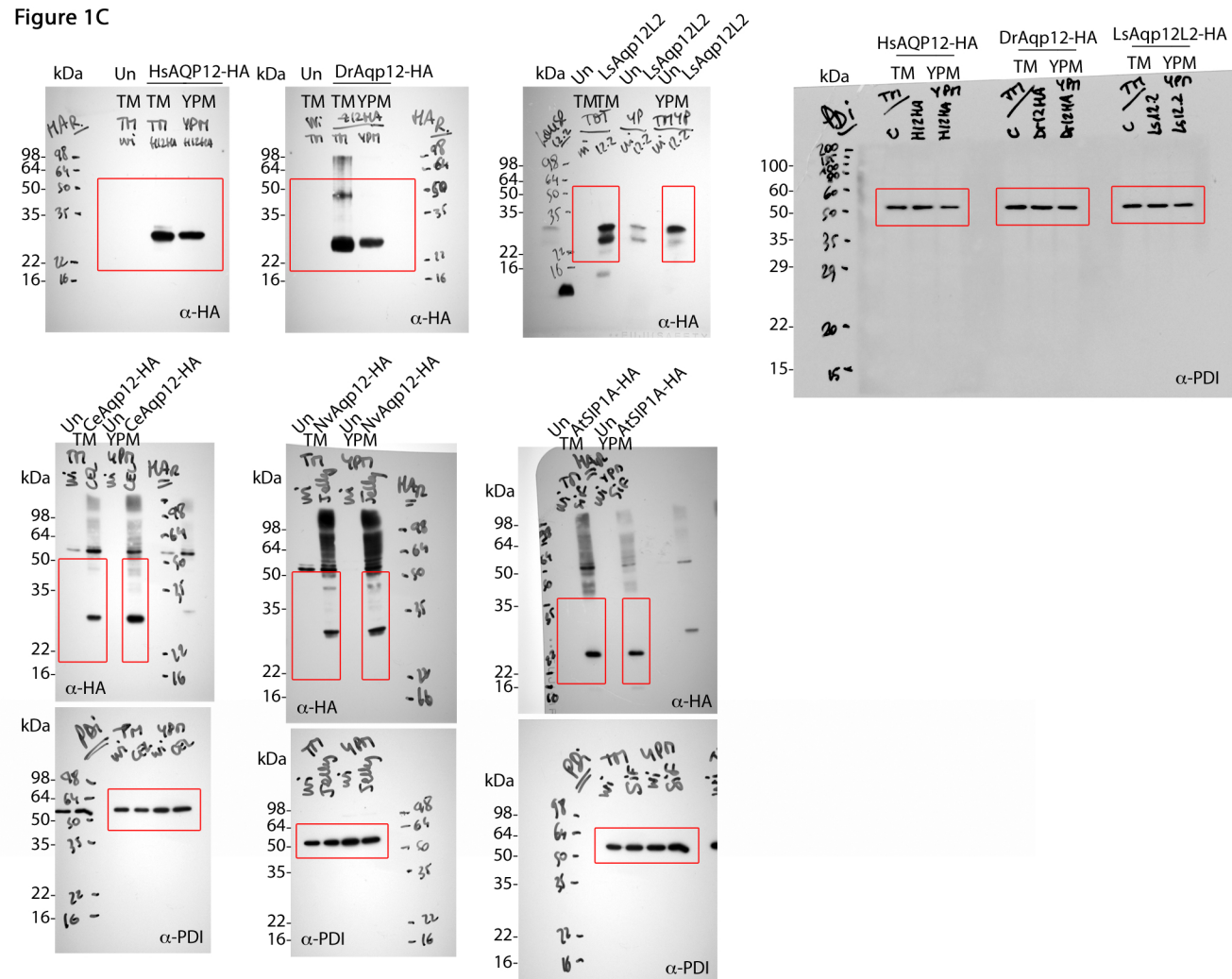

Supplement: SourceData F1 — is the source file for Fig. 1. [file jcb_202512040_sourcedataf1.pdf]

### Figure 2C

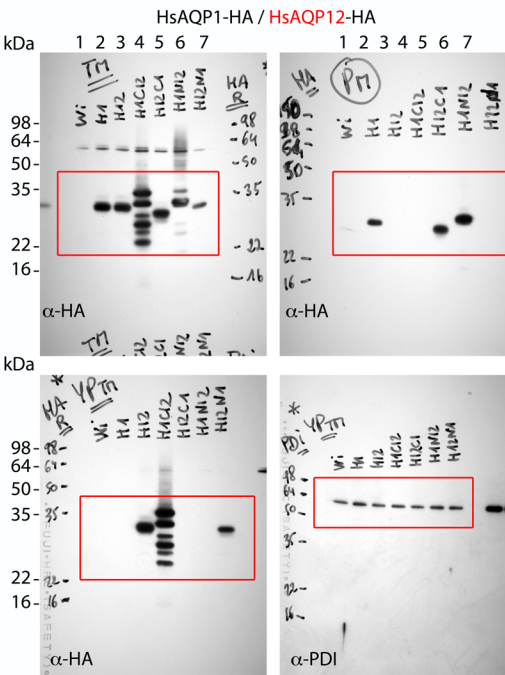

### Figure 2D

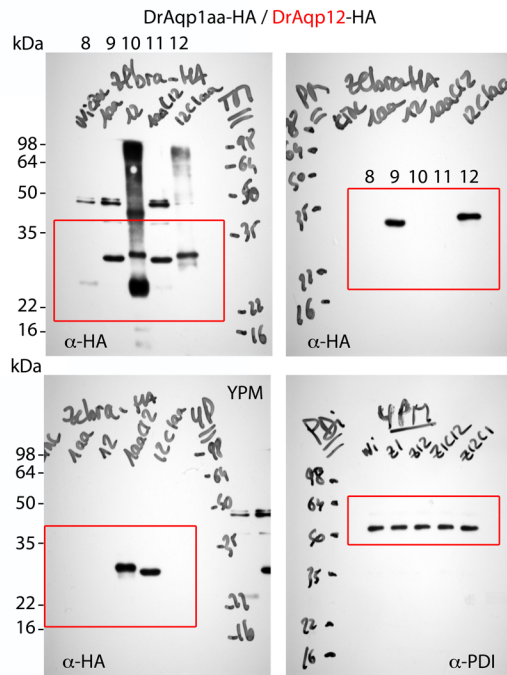

Figure 2H

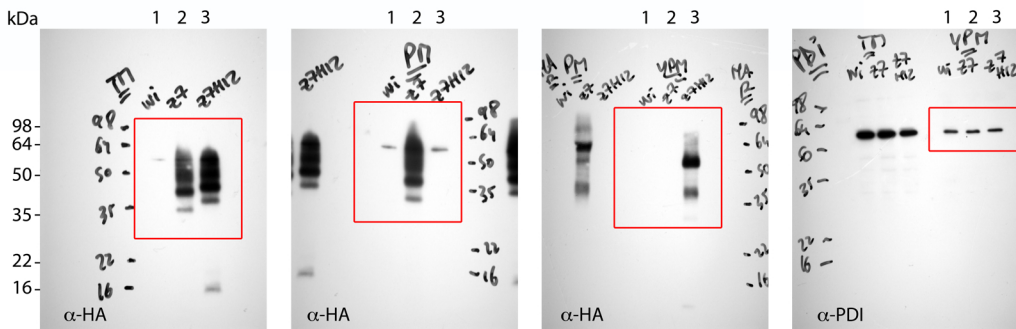

Supplement: SourceData F2 — is the source file for Fig. 2. [file jcb_202512040_sourcedataf2.pdf]

Figure 3A

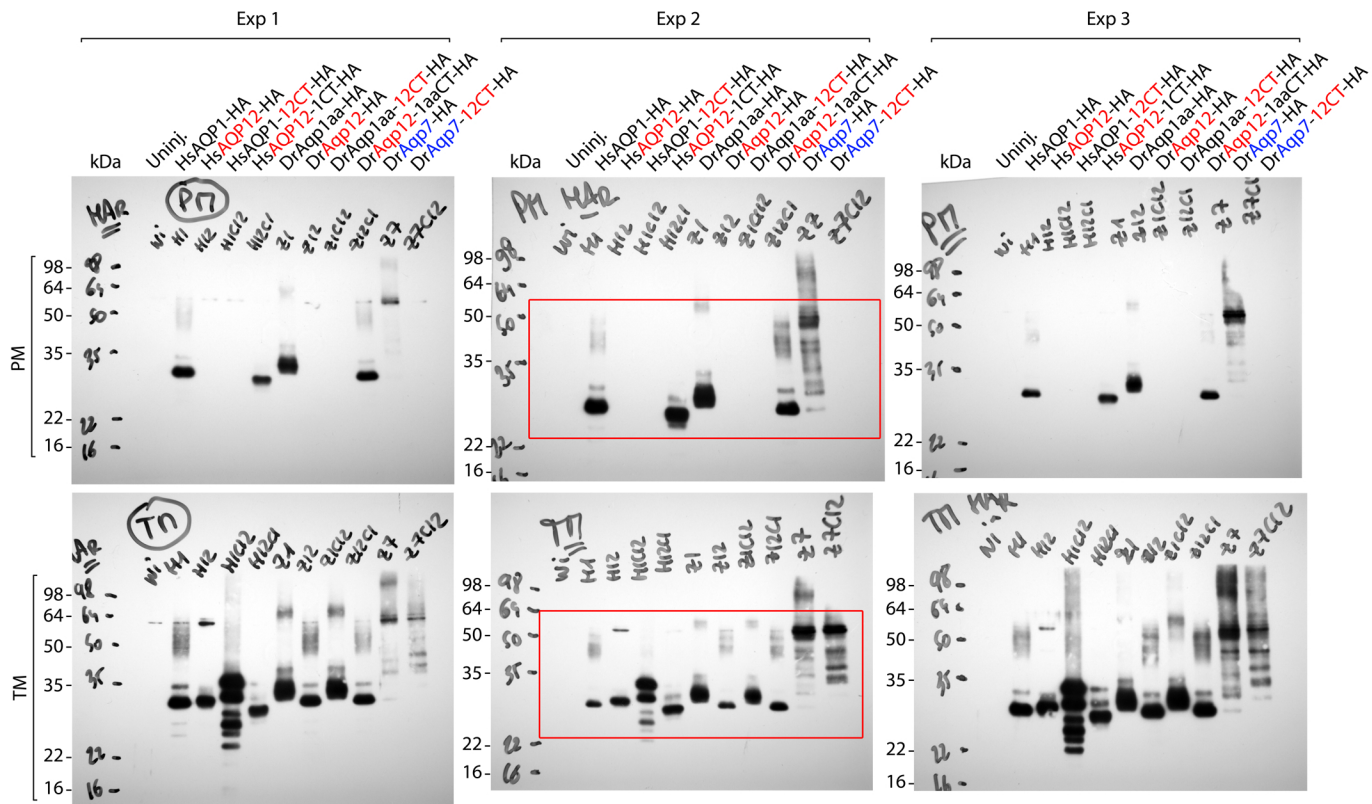

### Figure 3B

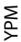

Figure 3E

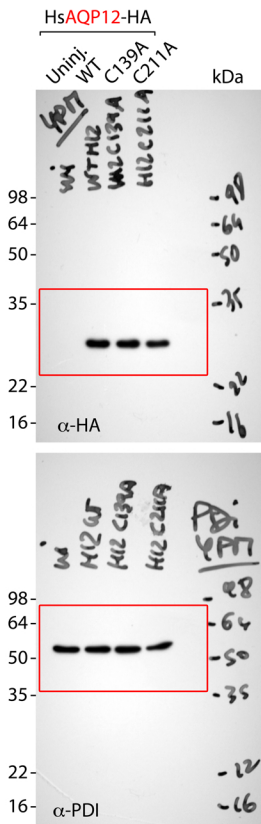

Figure 3H

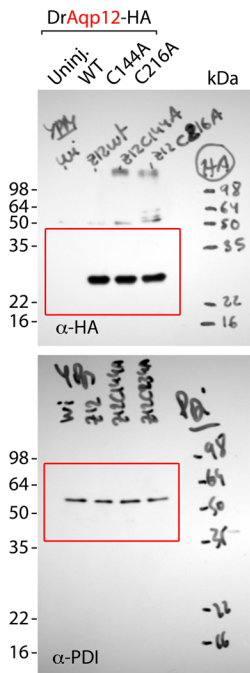

Supplement: SourceData F3 — is the source file for Fig. 3. [file jcb_202512040_sourcedataf3.pdf]

Figure 4C

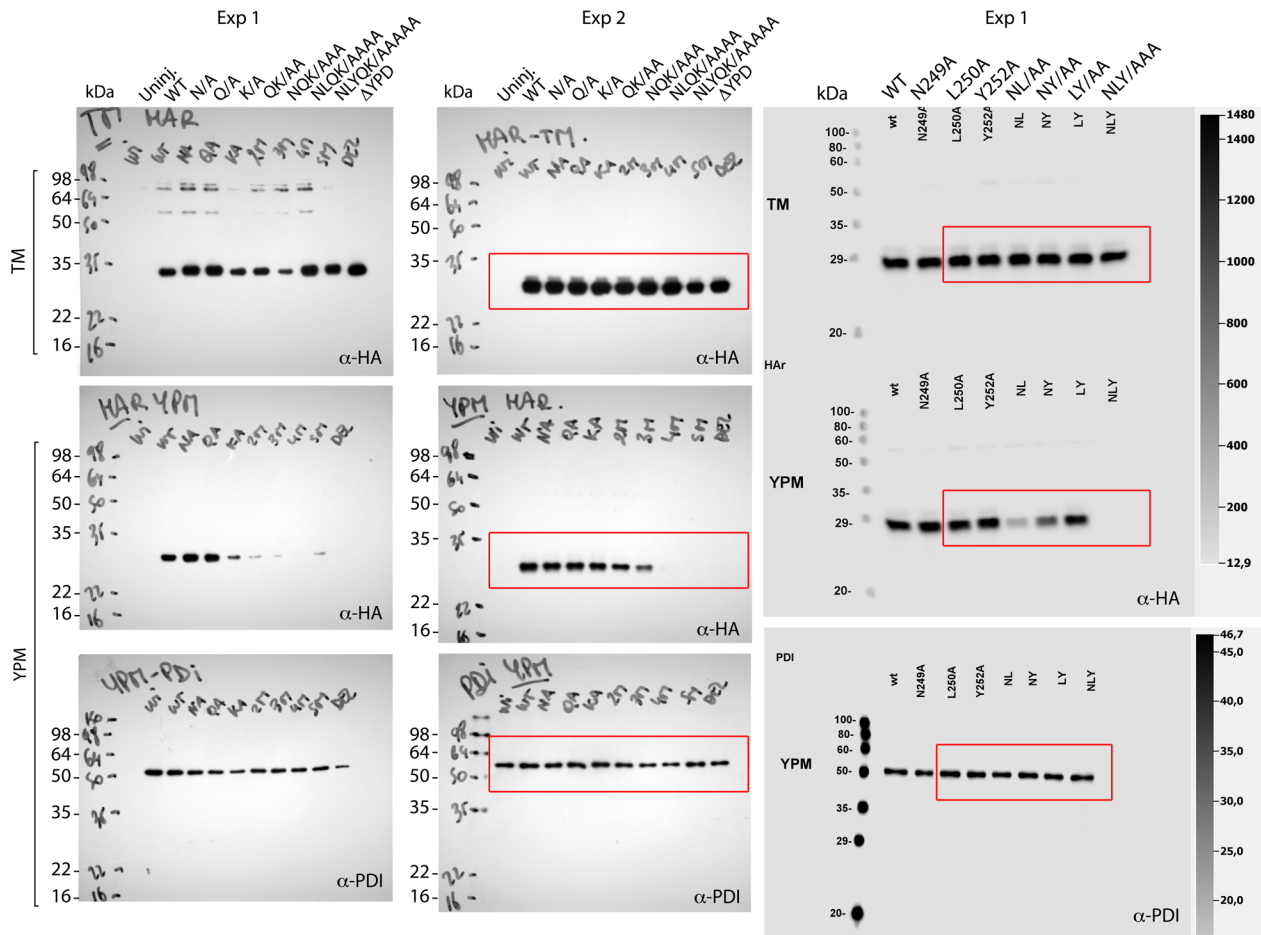

Figure 4F

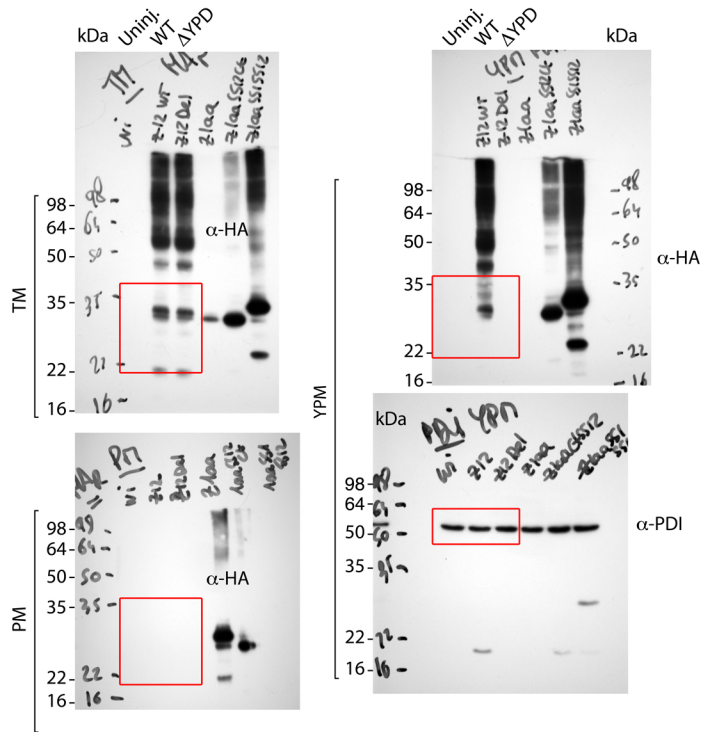

Figure 4J

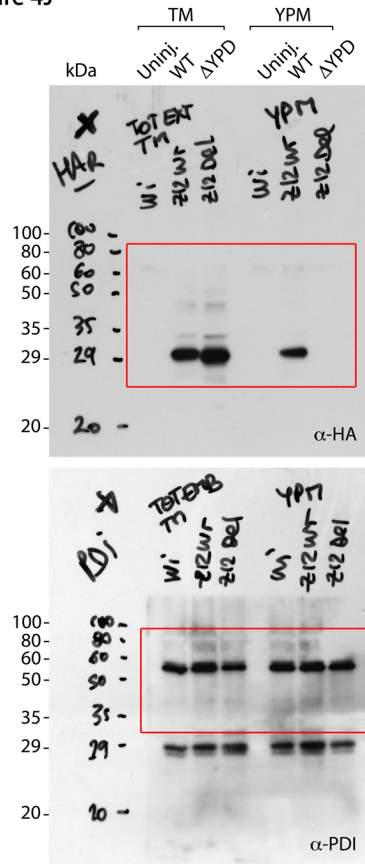

Supplement: SourceData F4 — is the source file for Fig. 4. [file jcb_202512040_sourcedataf4.pdf]

**Figure 6B**

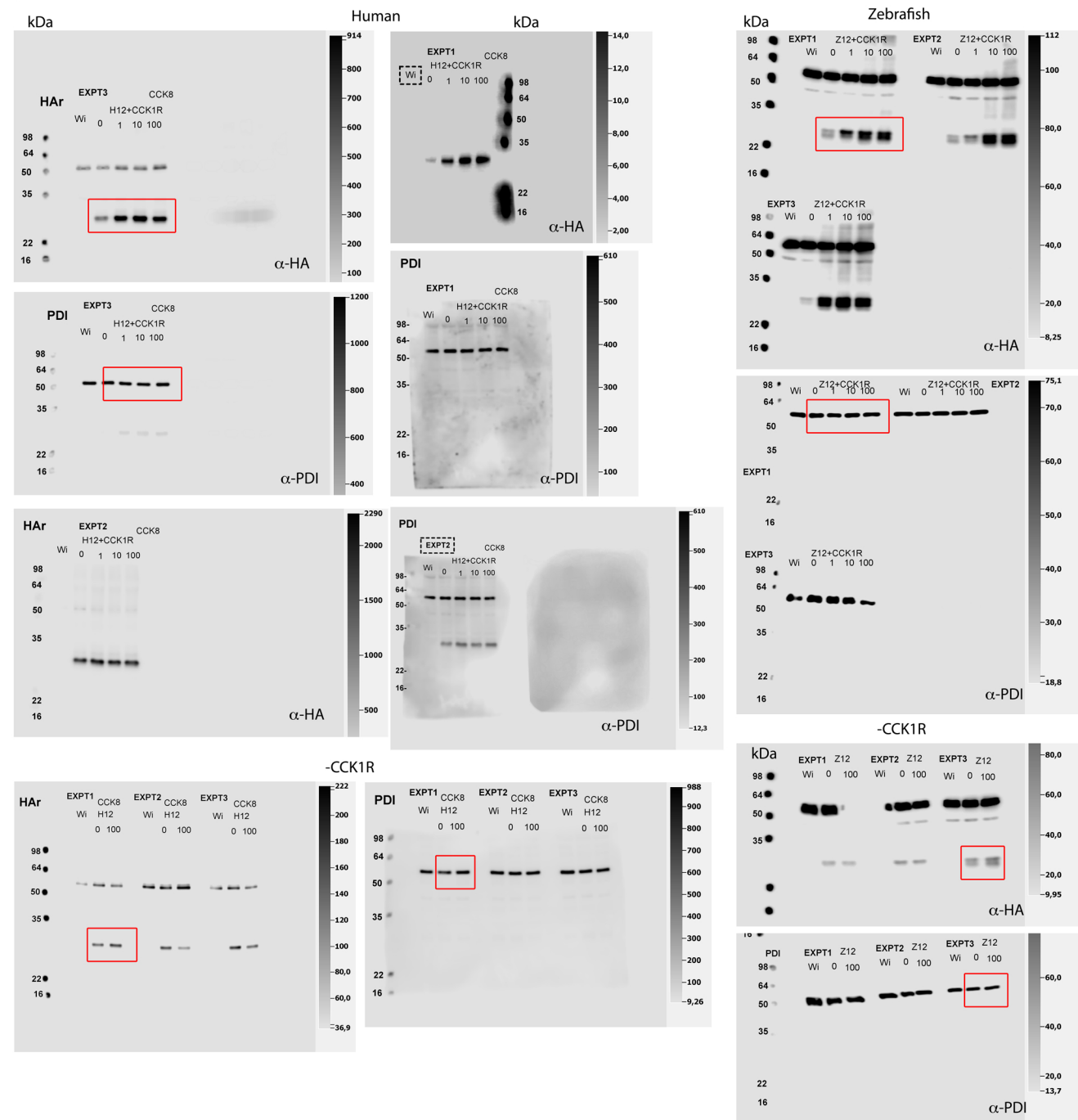

**Figure 6C**

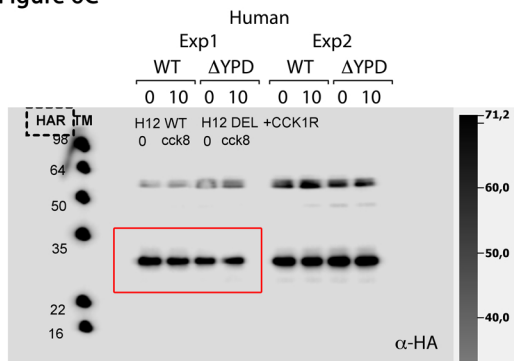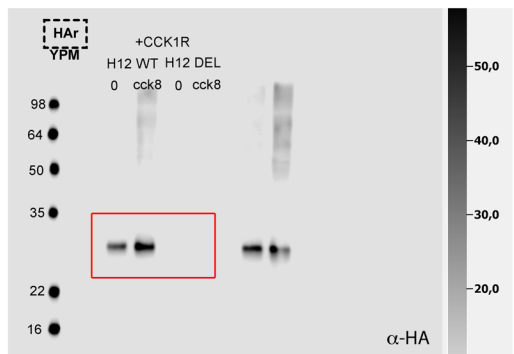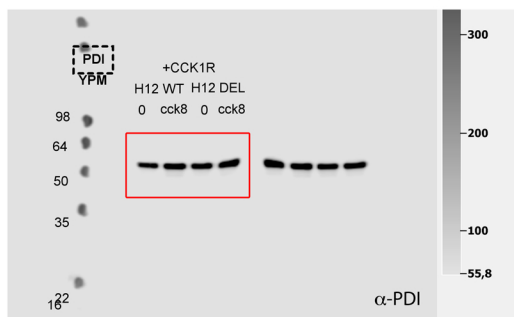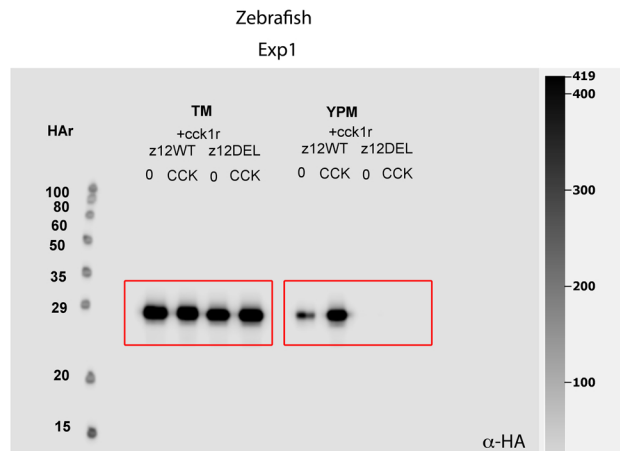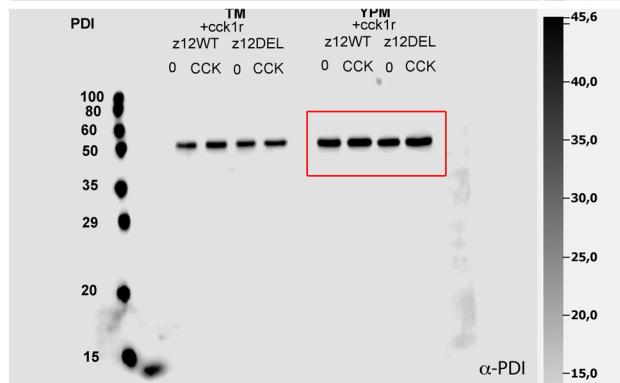

Figure 6D

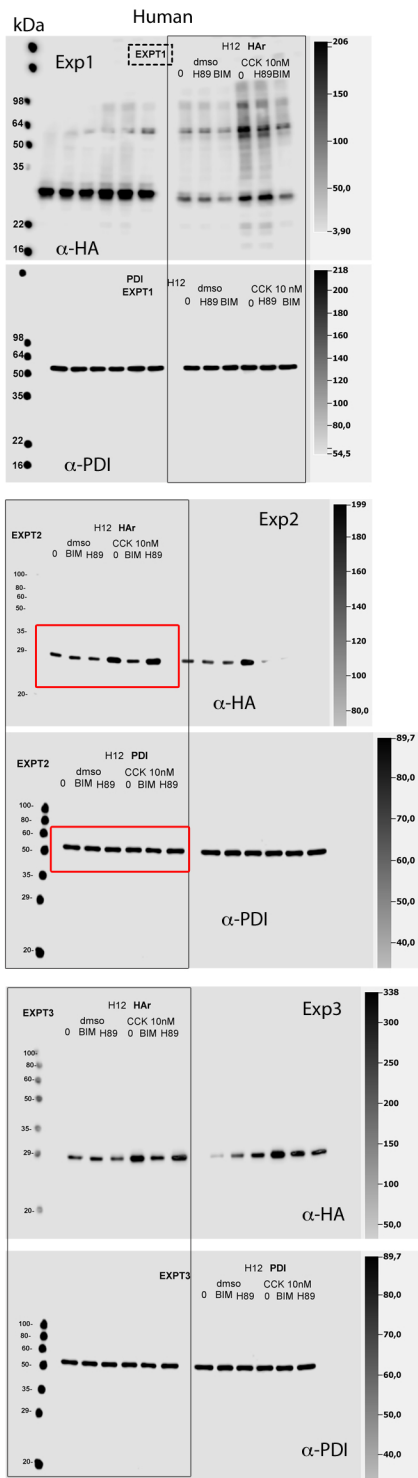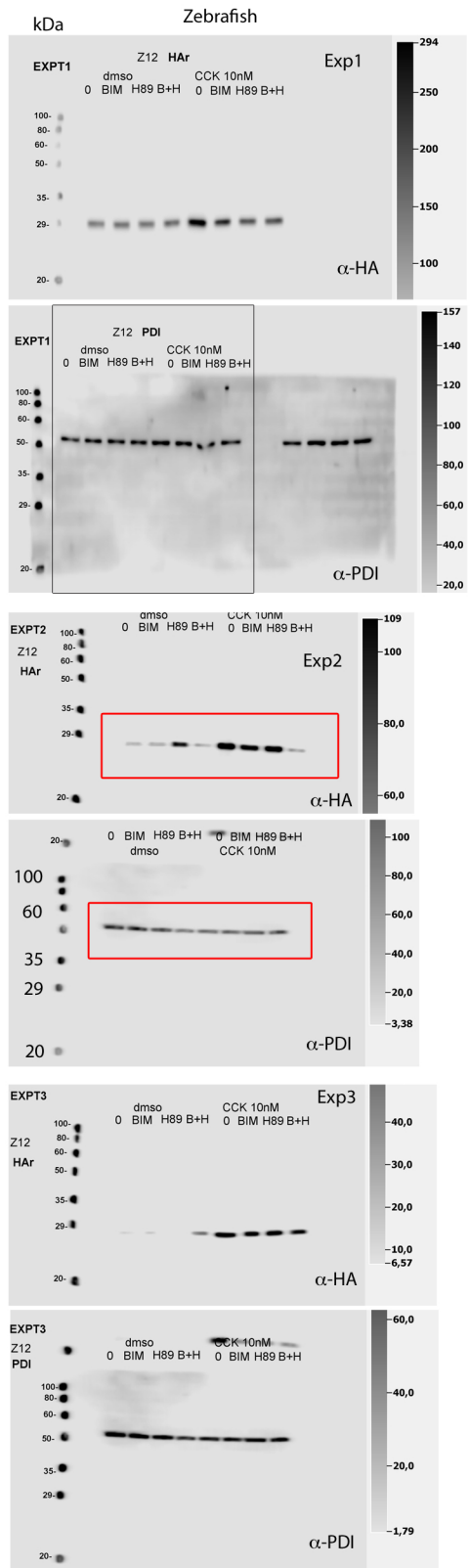

Figure 6E

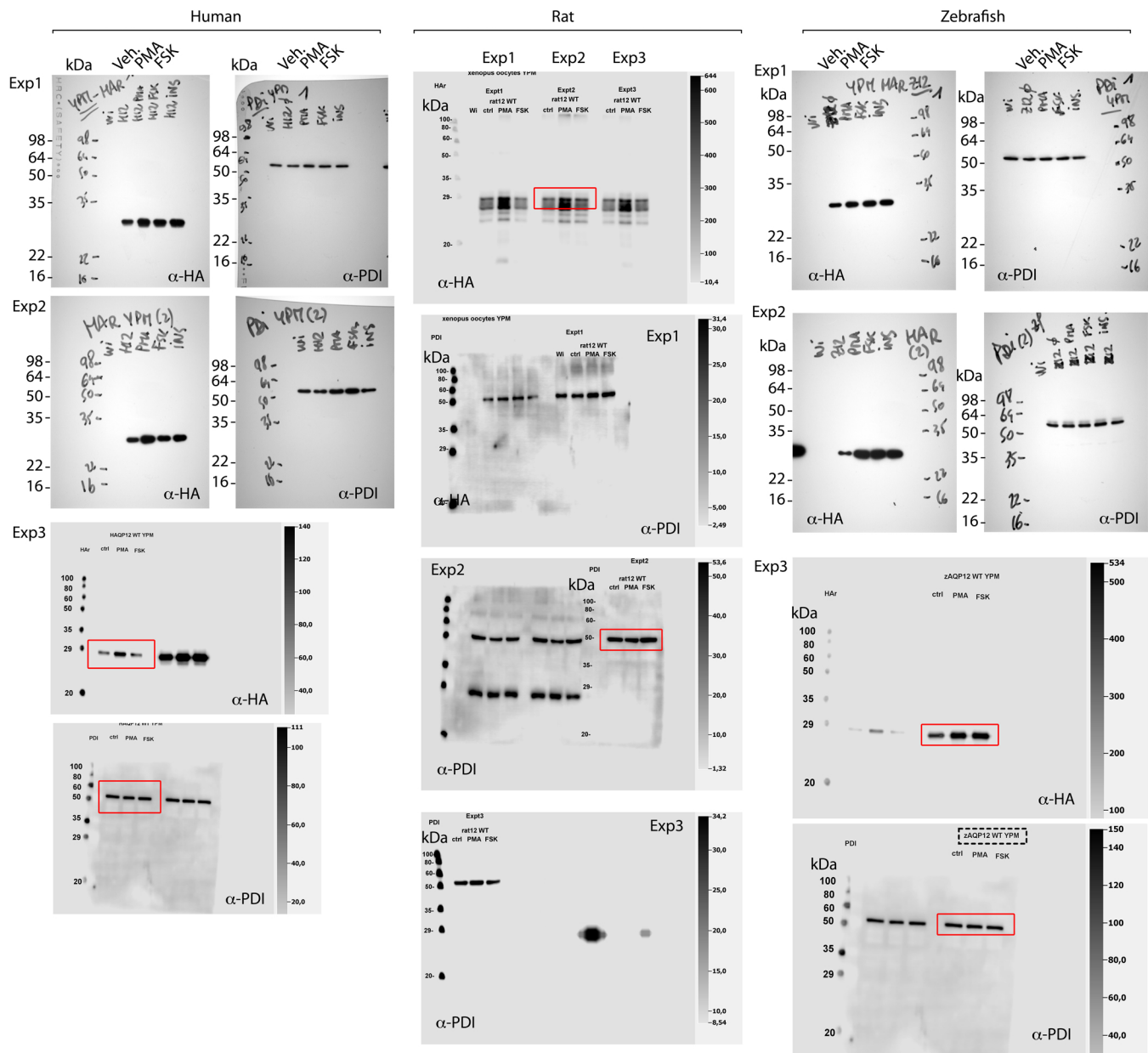

Supplement: SourceData F6 — is the source file for Fig. 6. [file jcb_202512040_sourcedataf6.pdf]

Figure 7B

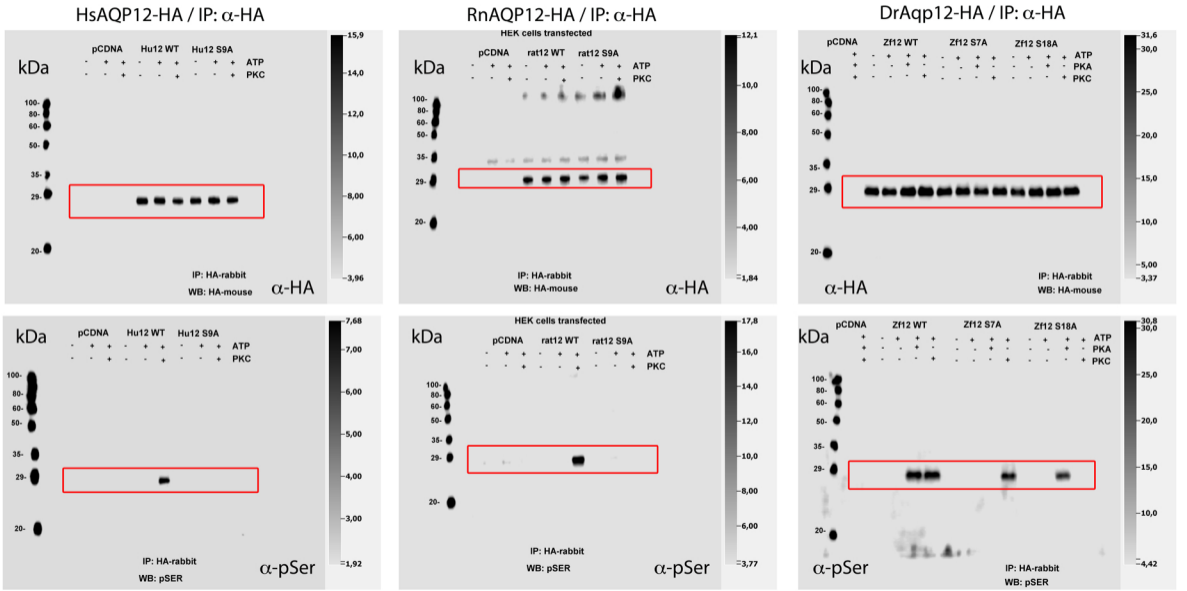

Figure 7C

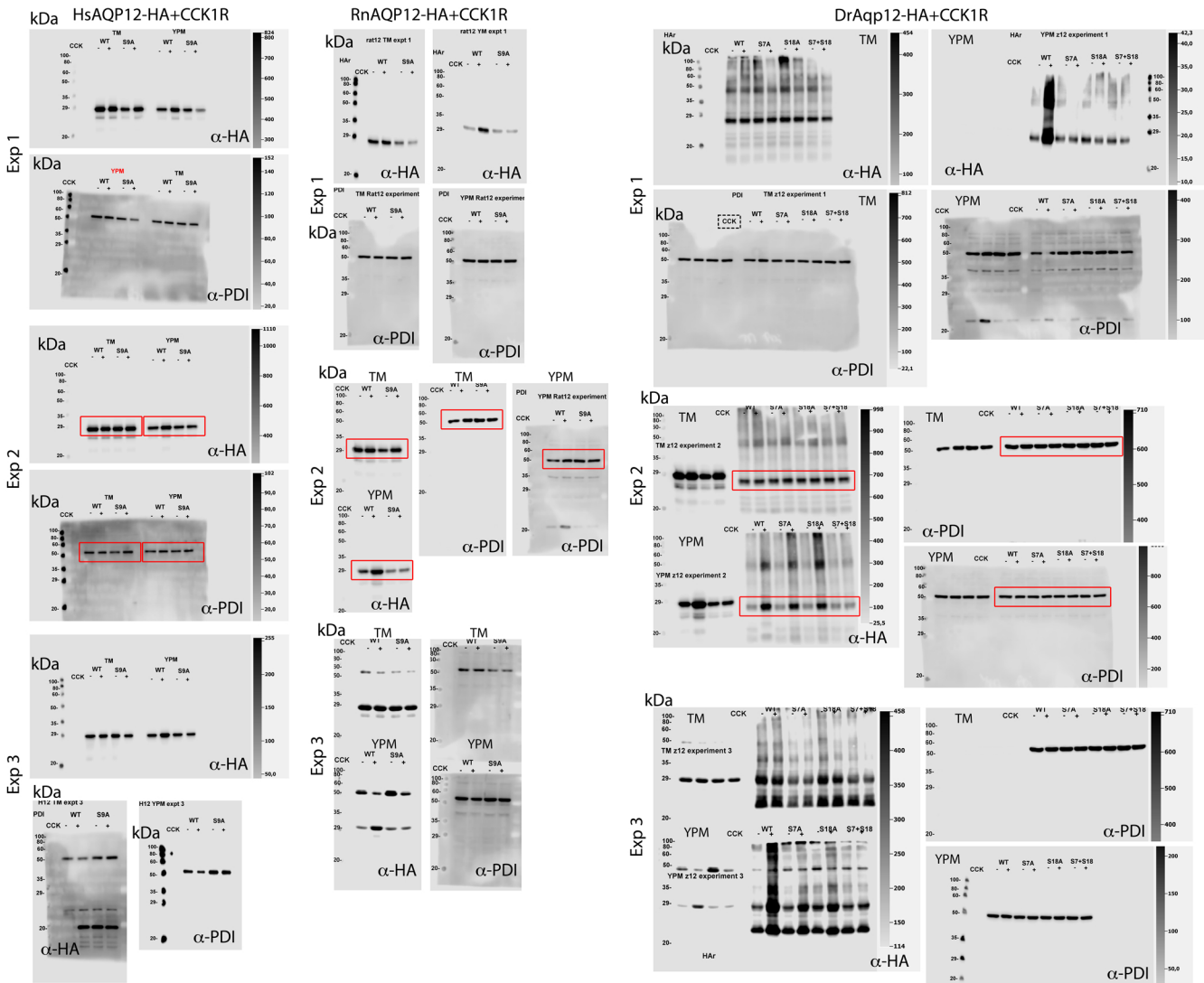

Supplement: SourceData F7 — is the source file for Fig. 7. [file jcb_202512040_sourcedataf7.pdf]

Figure 8E

Rat AQP12

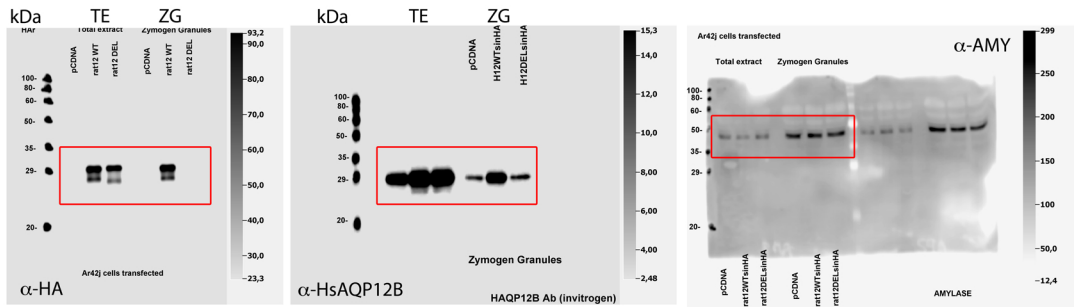

Human AQP12

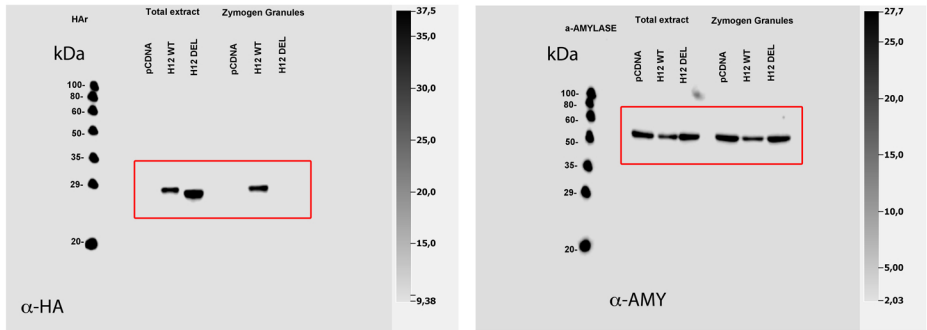

**Figure 8F**

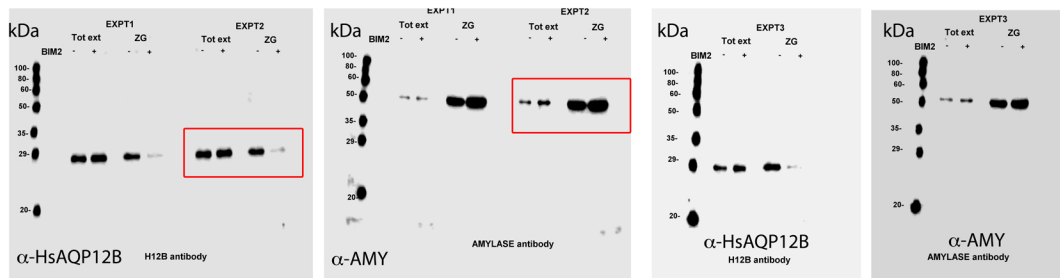

**Figure 8G**

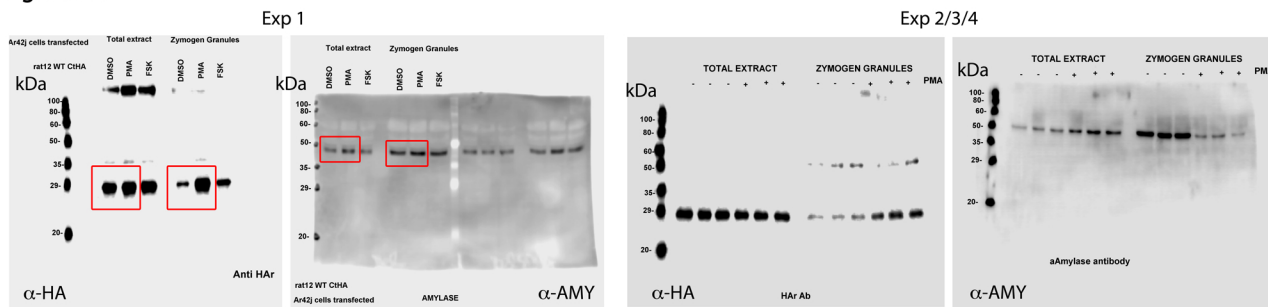

**Figure 8H**

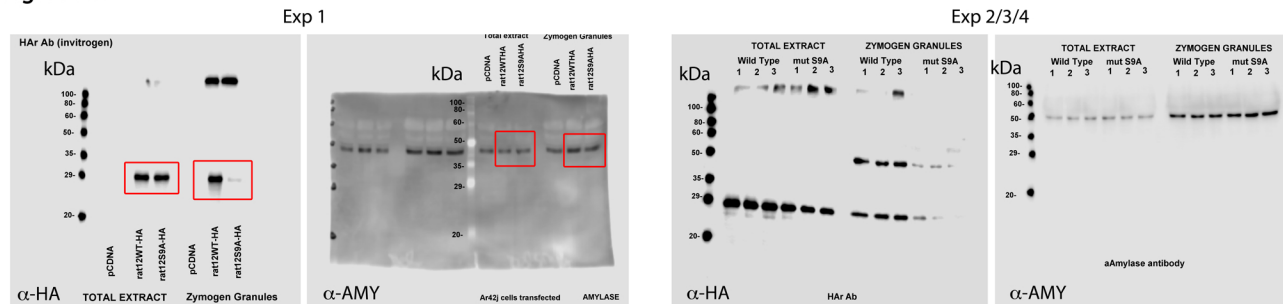

Supplement: SourceData F8 — is the source file for Fig. 8. [file jcb_202512040_sourcedataf8.pdf]

Figure 9I

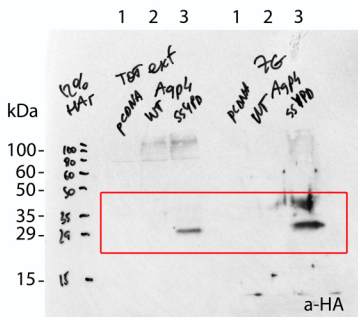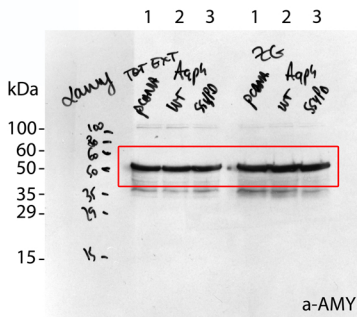

Figure 9J

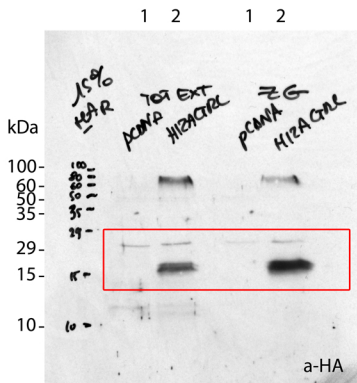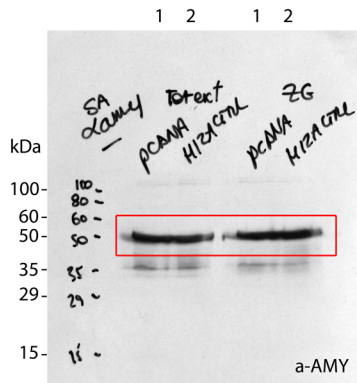

Supplement: SourceData F9 — is the source file for Fig. 9. [file jcb_202512040_sourcedataf9.pdf]

Figure S1B

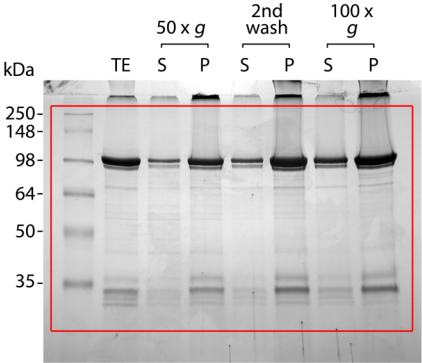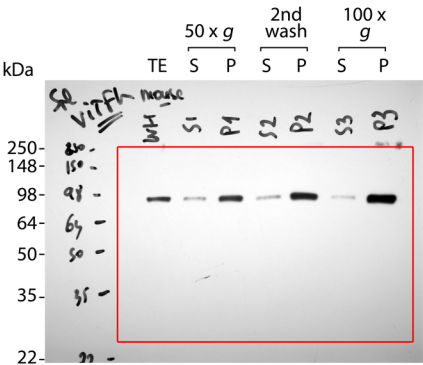

Supplement: SourceData FS1 — is the source file for Fig. S1. [file jcb_202512040_sourcedatafs1.pdf]

Figure S2L

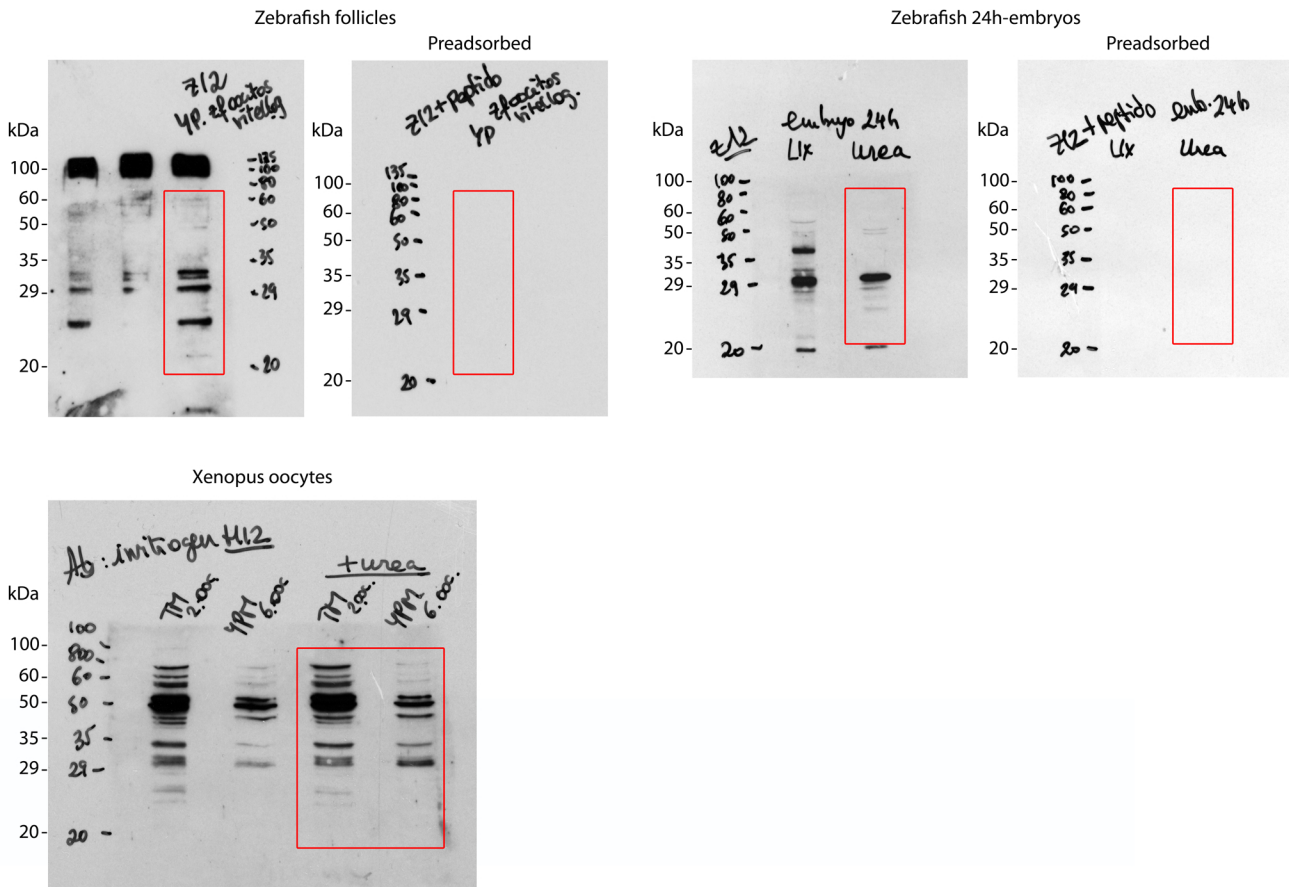

Supplement: SourceData FS2 — is the source file for Fig. S2. [file jcb_202512040_sourcedatafs2.pdf]

Figure S3B

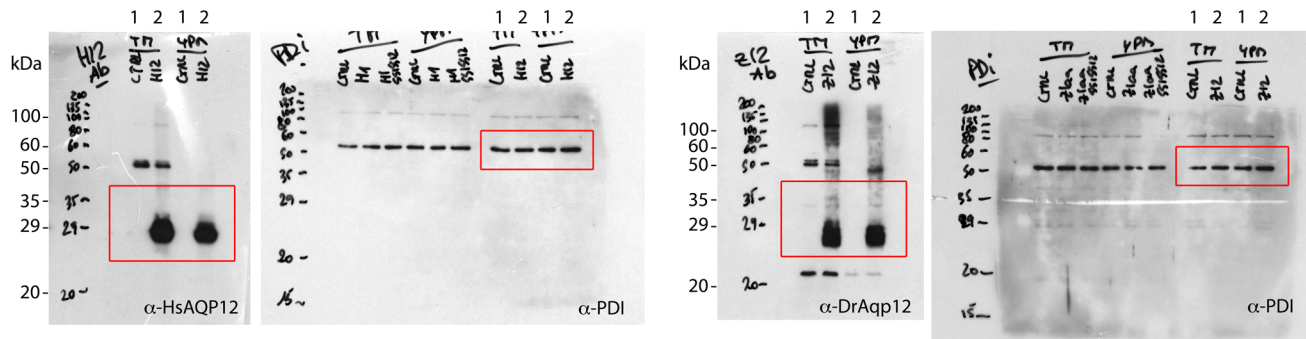

Figure S3E

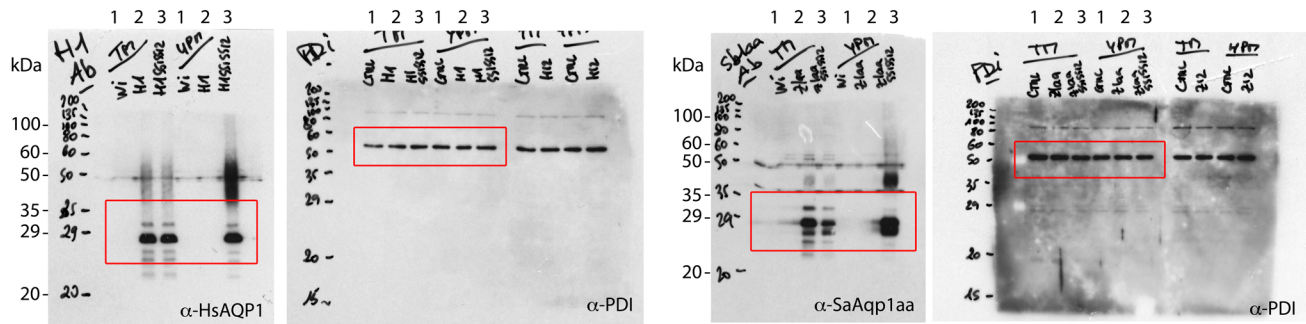

Supplement: SourceData FS3 — is the source file for Fig. S3. [file jcb_202512040_sourcedatafs3.pdf]
