# Supplementary material for: A pan-vertebrate signaling motif controls the molecular function of intracellular AQP12
Source: J Cell Biol. 2026 Jul 2;225(8):e202512040. doi: 10.1083/jcb.202512040 (PMC13344155; doi:10.1083/jcb.202512040)
Supplement: SourceData F5 — is the source file for Fig. 5. [file jcb_202512040_sourcedataf5.pdf]

Figure 5B

1: Uninjected
2: HsAQP12-WT-HA
3: HsAQP12-ΔYPD-HA
4: HsAQP1-WT-HA
5: HsAQP1-YPD-HA
6: HsAQP1-ΔSD-YPD-HA

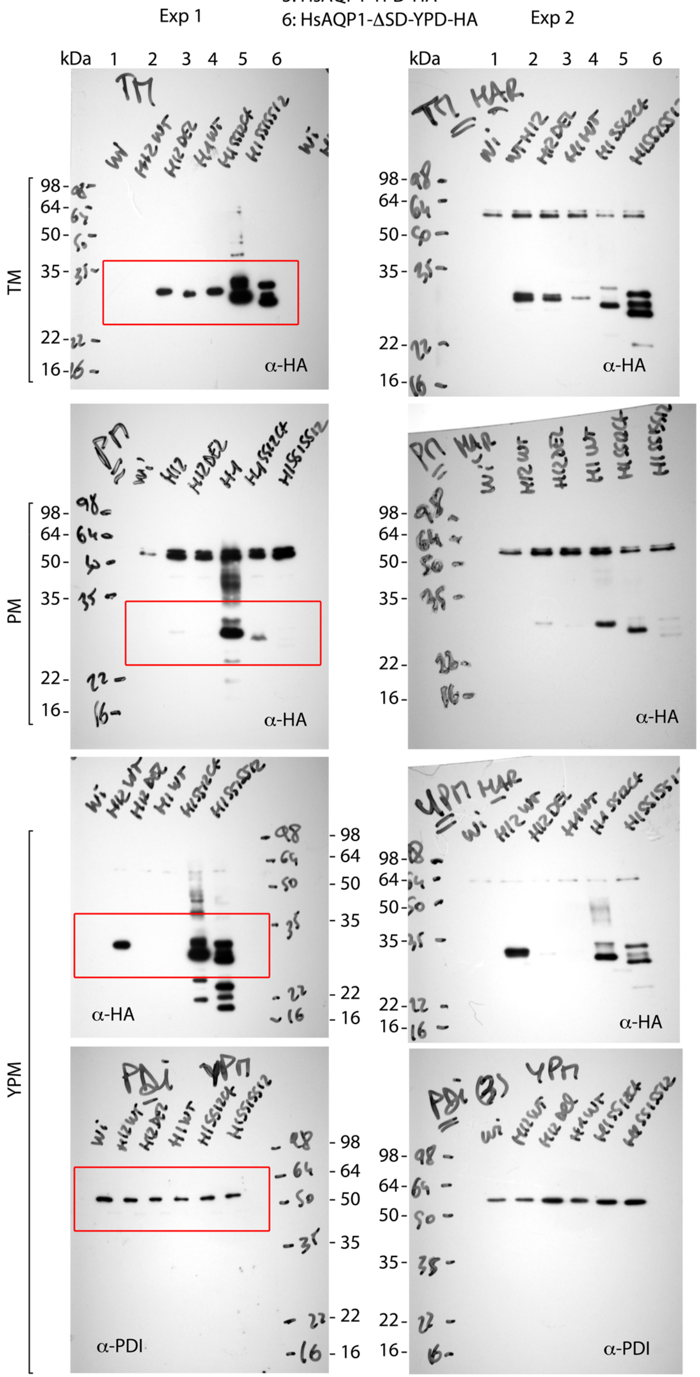

Figure 5F

1: Uninjected
2: DrAqp12-WT-HA
3: DrAqp12-ΔYPD-HA
4: DrAqp1aa-WT-HA
5: DrAqp1aa-YPD-HA
6: DrAqp1aa-ΔSD-YPD-HA

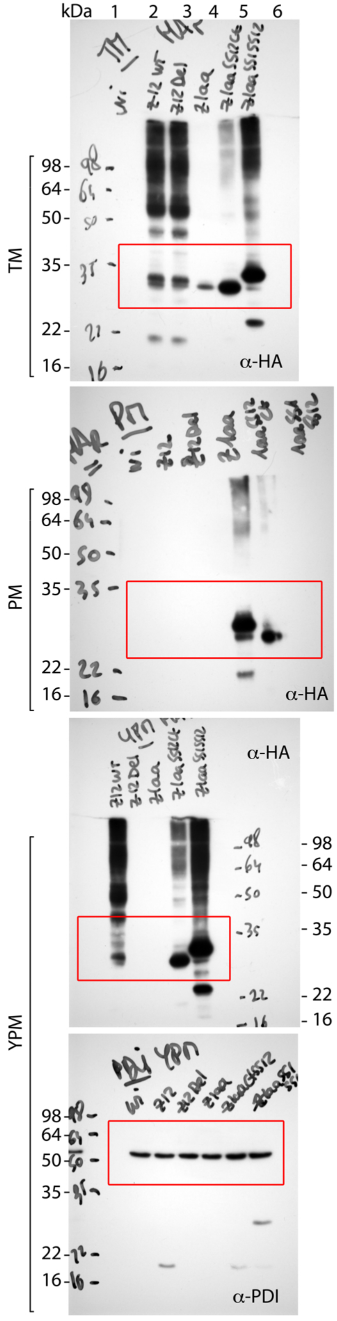

Figure 5J

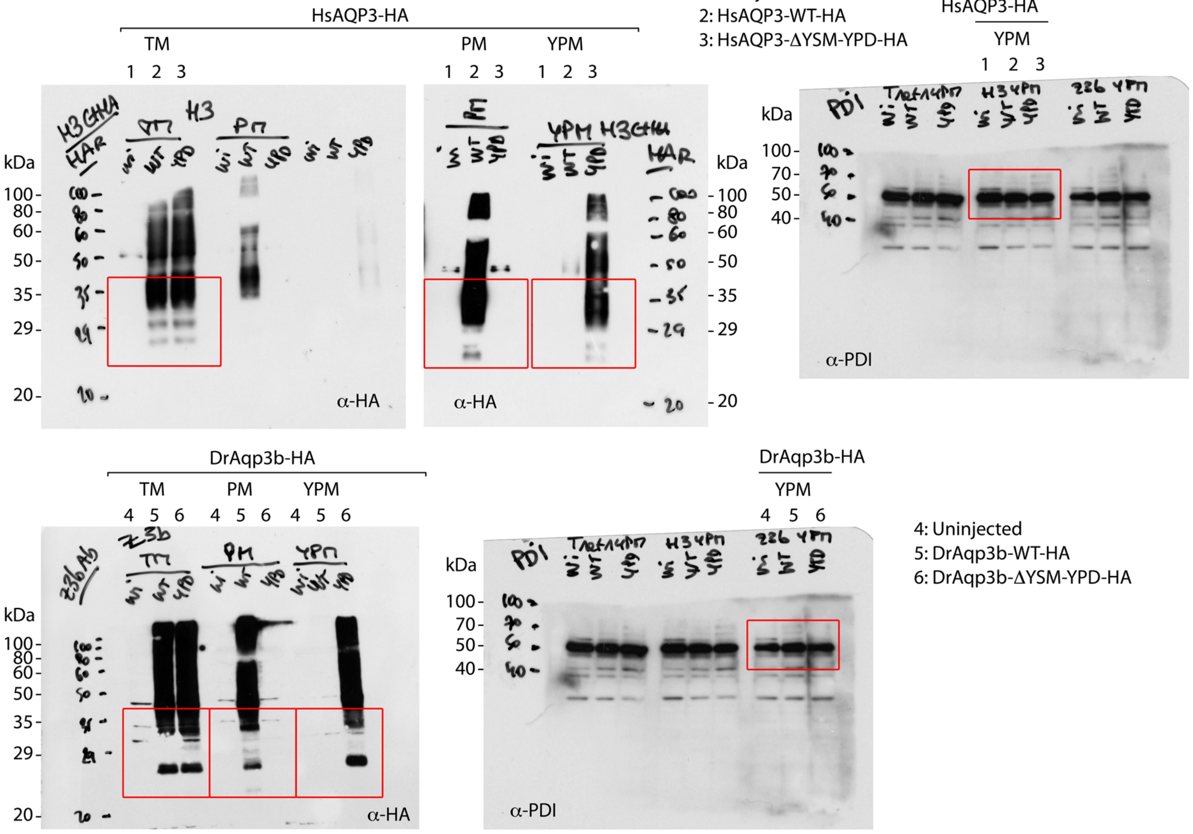

HsAQP3-HA
TM
PM
YPM
1 2 3
1 2 3
1 2 3
1: Uninjected
2: HsAQP3-WT-HA
3: HsAQP3-ΔYSM-YPD-HA
HsAQP3-HA
YPM
1 2 3
kDa
100
80
60
50
35
29
20
α-HA
α-HA
kDa
100
70
50
40
α-PDI
DrAqp3b-HA
TM
PM
YPM
4 5 6
4 5 6
4 5 6
DrAqp3b-HA
YPM
4 5 6
4: Uninjected
5: DrAqp3b-WT-HA
6: DrAqp3b-ΔYSM-YPD-HA
α-HA
α-PDI
