# Supplementary material for: A pan-vertebrate signaling motif controls the molecular function of intracellular AQP12
Source: J Cell Biol. 2026 Jul 2;225(8):e202512040. doi: 10.1083/jcb.202512040 (PMC13344155; doi:10.1083/jcb.202512040)
Supplement: Review History [file jcb_202512040_review_history.pdf]

JCB

A pan-vertebrate signaling motif controls the molecular function of intracellular AQP12

Francois Chauvigne, Marc Catalan-Garcia, Xavier Daura, Roderick Finn, and Joan Cerdà

Corresponding Author(s): Joan Cerdà, Institut de Ciències del Mar

Review Timeline:

Submission Date:	2025-12-04
Editorial Decision:	2026-02-10
Revision Received:	2026-03-20
Editorial Decision:	2026-04-14
Revision Received:	2026-04-23

Monitoring Editor: Elizabeth Miller

Scientific Editor: Dan Simon

Transaction Report:

(Note: With the exception of the correction of typographical or spelling errors that could be a source of ambiguity, letters and reports are not edited. The original formatting of letters and referee reports may not be reflected in this compilation.)

DOI: https://doi.org/10.1083/jcb.202512040

February 10, 2026

Re: JCB manuscript #202512040

Joan Cerdà
Institut de Ciències del Mar

Dear Dr. Cerdà,

Thank you for submitting your manuscript entitled "A pan-vertebrate signaling motif controls the molecular function of the intracellular AQP12." The manuscript was assessed by expert reviewers, whose comments are appended to this letter. We invite you to submit a revision if you can address the reviewers' key concerns, as outlined here.

The comments that are essential to address are the request to add a water negative control to several experiments (Rev1#3), clarification of whether the HA tag near the new trafficking motif might affect its function (Rev3#1), and to explain the apparently slow activity of APQ12 (Rev3#4,5). Please also answer Rev1#4 which asks about equivalent gel loading, although new data may not be needed since PDI is a suitable loading control. Please also see below about the journal's requirements to submit unprocessed and uncropped images for all blots and gels in a paper. Understanding the role of the N-term phosphosites in more detail (Rev3#3) is certainly important but is not necessary for this paper. Please also address all other comments asking for text changes to clarify and better explain the findings. However, in the membrane field, the term 'translocation' refers to transfer of proteins across a membrane so 'trafficking' is more appropriate to describe the transport of a protein to specific cellular locations.

While you are revising your manuscript, please also attend to the following editorial points to help expedite the publication of your manuscript. Please direct any editorial questions to the journal office.

GENERAL GUIDELINES:

Text limits: Character count for an Article is < 40,000, not including spaces. Count includes title page, abstract, introduction, results, discussion, and acknowledgments. Count does not include materials and methods, figure legends, references, tables, or supplemental legends.

Figures: Articles may have up to 10 main text figures. Figures must be prepared according to the policies outlined in our Instructions to Authors, under Data Presentation, https://jcb.rupress.org/site/misc/ifora.xhtml. All figures in accepted manuscripts will be screened prior to publication.

IMPORTANT: It is JCB policy that if requested, original data images must be made available. Failure to provide original images upon request will result in unavoidable delays in publication. Please ensure that you have access to all original microscopy and blot data images before submitting your revision.

Supplemental information: There are strict limits on the allowable amount of supplemental data. Articles may have up to 5 supplemental figures. Up to 10 supplemental videos or flash animations are allowed. A summary of all supplemental material should appear at the end of the Materials and methods section.

Please note that JCB now requires authors to submit Source Data used to generate figures containing gels and Western blots with all revised manuscripts. This Source Data consists of fully uncropped and unprocessed images for each gel/blot displayed in the main and supplemental figures. For assays performed using capillary electrophoresis and/or immunoassay-based detection, authors should instead provide the electropherogram graph(s) for each experiment, plotting fluorescence/chemiluminescence intensity vs. molecular weight/size. Please be sure to provide one Source Data file for each figure gels, blots, and/or capillary electrophoresis assays along with your revised manuscript files. File names for Source Data figures should be alphanumeric without any spaces or special characters (i.e., SourceDataF#, where F# refers to the associated main figure number or SourceDataFS# for those associated with Supplementary figures). For traditional gels and blots, the lanes of the gels/blots should be labeled as they are in the associated figure, the place where cropping was applied should be marked (with a box), and molecular weight/size standards should be labeled wherever possible. For capillary electrophoresis assays, each trace in the graph should be color-coded and labeled to indicate which protein, gene, or sample is being measured (please try to avoid red/green combinations to accommodate our color-blind readers).

Source Data files will be made available to reviewers during evaluation of revised manuscripts and, if your paper is eventually published in JCB, the files will be directly linked to specific figures in the published article.

Source Data Figures should be provided as individual PDF files (one file per figure). Authors should endeavor to retain a minimum resolution of 300 dpi or pixels per inch. Please review our instructions for export from Photoshop, Illustrator, and

PowerPoint here: https://rupress.org/jcb/pages/submission-guidelines#revised

The typical timeframe for revisions is three to four months. If you anticipate any difficulties in meeting this aforementioned revision time limit, please contact us and we can work with you to find an appropriate time frame for resubmission. Please note that papers are generally considered through only one revision cycle, so any revised manuscript will likely be either accepted or rejected.

When submitting the revision, please include a cover letter addressing the reviewers' comments point by point. Please also highlight all changes in the text of the manuscript.

We hope that the comments below will prove constructive as your work progresses. We would be happy to discuss them further once you've had a chance to consider the points raised in this letter.

Thank you for this interesting contribution to Journal of Cell Biology. You can contact us at the journal office with any questions at cellbio@rockefeller.edu.

Sincerely,

Elizabeth Miller, PhD
Monitoring Editor
Journal of Cell Biology

Dan Simon, PhD
Scientific Editor
Journal of Cell Biology

Reviewer #1 (Comments to the Authors (Required)):

JCB - Journal of Cell Biology
A pan-vertebrate signaling motif controls the molecular function of the intracellular AQP12.

Summary of the main findings of the study.
Chauvigné et al presents a research article where they characterize the aquaporin homologue AQP12 with respect to function and regulation. This is a relatively uncharacterized protein within the aquaporin family of membrane proteins, and the manuscript therefore contributes to the overall understanding of aquaporin molecular mechanism.

Highlighted limitations and strengths.
The study presented by Chauvigné et al is a very thorough investigation of the unorthodox AQP12 studying various isoforms in the well-established system for aquaporin characterization: oocytes from Xenopus laevis. The authors investigated their localisation as well as selectivity and inhibition of passive transport. Furthermore, by comparing close to 500 sequences, a translocation signal was identified, which could substitute the sorting signal in the orthodox aquaporin AQP1. Finally, the phosphorylation and signaling pathways have been investigated as well as pancreatic acinar cells.
The manuscript contains a substantial amount of data which is clearly described and analysed as well as supported by nice figures. I conclude that the study maintains high quality and I only have a few comments that I would like the authors to reflect upon, please see below.

Comments on the methods, results and data interpretation. Any objective errors, or if the conclusions are not supported, concerns are given in detail.
Overall, I think the results are well supported by the data and hence the conclusions valid. The interpretation of the data is generally sound, but the interpretation of the selectivity and inhibition of passive transport requires some additional reflections, please see comment 4 below.

Detailed review report to the editor and authors:
1. May I suggest using 'translocation' rather than 'trafficking'.
2. Page 15. AQP4 is picked as an example of an aquaporin isoform that is not endogenously expressed in pancreatic acinar cells, and the M23 isoform is selected. Please argue for not using the M1 isoform and possible implications of that.
3. In figure 3I a water control is included in the analysis. It would have been preferred to include such a negative control also in Fig. 1, Fig. 3D and G, as well as Fig. 4C and G. The same comment is valid for the AR42J cells in Fig. 7 and 8.
4. Pages 28-29. Protein extraction and Immunoblot analysis. How do you assure that the same amount of protein is loaded for each sample? Also, in the Figures, a very narrow area is commonly shown for the Immunoblots while the full lane should ideally be presented. Furthermore, a size estimate for the band should be provided, this is not always the case.
Related to this reflection, a very important aspect needs to be highlighted and that is the requirement of relating the function to

the amount and stability of the protein, this is especially valid for the data in Fig. 2. This is a critical aspect to include in the results described on page 7.

Reviewer #2 (Comments to the Authors (Required)):

The authors present a study of the permeability and membrane trafficking of AQP12 and analogues thereof.
The permeability properties of the unorthodox aquaporins has been a long-standing open question in the aquaporin field due to their intracellular localisation. The authors have addressed this question by developing a method to measure permeability in the oocyte yolk membrane, and by manipulating the C-terminus to give plasma membrane localisation. Importantly the permeability measurements for the native channels in the yolk membrane are in good agreement with the permeability measurements for the C-terminal substituted channels in whole oocytes.
The authors further identify a sequence motif (YPD) responsible for yolk membrane targeting and show that this motif targets aquaporins to the zymogen granule membrane in a rat pancreatic cell line.

Overall this is a well conducted study with clearly-presented data and conclusions that are robustly supported by the data. The results address some important open questions in the aquaporin field.

I have some minor suggestions and questions for improvement of the manuscript:

1. The authors have used a rat cell line to study the ZG localisation of YPD-containing aquaporins. From a translational perspective it would be very useful to determine if the same effect of the YPD motif is seen in human cells. Have the authors checked this or any plans to do so?

2. In mammalian cells that do not have zymogen granules, what is the trafficking destination of YPD-containing aquaporins? Do the authors have any data to address this or could they speculate?

3. Do the authors have any hypotheses or speculation about what is the component(s) of the membrane trafficking machinery that recognises the YPD motif?

4. Have the authors performed any bioinformatic analyses to look for YPD motifs across the proteome. It would be interesting to know whether this is an aquaporin-specific motif or if it is used by other classes of membrane proteins.

5. To assist the reader, the authors could mention that the unorthodox aquaporins are also referred to as "superaquaporins" in some literature.

Reviewer #3 (Comments to the Authors (Required)):

Chauvigné et al. present a comprehensive study on a putative novel C-terminal trafficking motif of various aquaporin-12 isoforms that initiate translocation of the channel proteins to intracellular membranes of yolk platelets (X. laevis and zebra fish oocytes) or zymogen granules (pancreatic acinar cells), respectively. Ser-phosphorylation in the N-terminal region appears to be an additionally requirement for trafficking. The authors used isolated X. laevis yolk platelets for AQP12 permeability assays by which they found water, hydrogen peroxide, methylamine and urea to pass as substrates. They further used pharmacological tools (activators, inhibitors) to investigate PKA/PKC signaling pathways underlying AQP12 trafficking. Introduction of the motif into membrane proteins that are originally located at the plasma membrane resulted in re-localization to intracellular sites. Apparently, a lot of work went into the study and it seems quite conclusive.

Some points require clarification:

1. With the identified motif located at the C-terminus of the AQP12 isoforms it is unfortunate that all used constructs contain a hemagglutinin epitope tag at the C-terminus as well. At which positions does the motif work (N-terminus, loops)? How does an epitope-free C-terminus affect the data?

2. Which other yolk/zymogen-associated or other proteins carry the motif? Or do the authors expect an exclusive mechanism for AQP12?

3. Besides action of the motif, phosphorylation in the N-terminal region appears to be required. What would a mechanism look like that addresses N- and C-terminal sites for trafficking? Are those sites close to each other and bind to the same translocating machinery? Is there a pool of native AQP12 protein somewhere in the cells before it is integrated into the organelles?

4. Water permeability assays with yolk platelets were done using tritium-labeled water and an incubation time of 10 min. AQP facilitated water flux in such small organelles should be finished below or within a few seconds, with an AQP one would still expect a seconds timescale. How does the platelet membrane in the absence of an AQP exclude water exchange by transmembrane diffusion for such a long period of time?

5. The authors speculate about roles of an AQP in the yolk platelet and zymogen granule membranes. They state osmotic swelling as a requirement for membrane fusion. What is the origin of an osmotic gradient in the organelles and why would an AQP help in the process? This goes along with point 3, above. AQPs increase the water flux velocity up to one order of magnitude in the range of sub-second to seconds. However, this eventually results in the same steady-state situation.

6. Another prominent intracellular AQP is isoform 6. Its functionality is mainly ion permeability. In view of the points above, are there structural hints in the AQP12 family that point towards ion permeability?

7. Urea is a peculiar AQP substrate due to its relatively large plus rigid and flat molecular structure, which requires special adaptations in the channel path. Is this type of permeability a by-product by chance?

Editor comments

The comments that are essential to address are the request to add a water negative control to several experiments (Rev1#3), clarification of whether the HA tag near the new trafficking motif might affect its function (Rev3#1), and to explain the apparently slow activity of APQ12 (Rev3#4,5). Please also answer Rev1#4 which asks about equivalent gel loading, although new data may not be needed since PDI is a suitable loading control.

We have added the immunostaining of the uninjected control oocytes in several figures (Rev1#3), and performed additional experiments to address whether the HA tag may affect the trafficking of the channels (Rev3#1). We have also addressed the comments of Rev3#4,5 and Rev1#4. Please, see below our responses for all the reviewer's remarks.

Please also see below about the journal's requirements to submit unprocessed and uncropped images for all blots and gels in a paper.

Uncropped images for all gels and immunoblots are now reported in the different SourceData Files (F1-F9 and FS1-FS3).

Understanding the role of the N-term phosphosites in more detail (Rev3#3) is certainly important but is not necessary for this paper.

See below our response for this comment from Rev3#3.

Reviewer #1

1) May I suggest using 'translocation' rather than 'trafficking'.

As the editor points out, in the membrane field the term 'translocation' refers to transfer of proteins across a membrane, therefore 'trafficking' is more appropriate to describe the transport of AQP12 to the YPs or ZGs.

2) Page 15. AQP4 is picked as an example of an aquaporin isoform that is not endogenously expressed in pancreatic acinar cells, and the M23 isoform is selected. Please argue for not using the M1 isoform and possible implications of that.

The AQP4-M23 isoform was selected because in contrast to the M1 isoform M23 has the ability to stabilize orthogonal arrays of particles in the plasma membrane and facilitate the formation of wider arrays. This is now indicated in the text (lines 483-486).

3) In figure 3I a water control is included in the analysis. It would have been preferred to include such a negative control also in Fig. 1, Fig. 3D and G, as well as Fig. 4C and G. The same comment is valid for the AR42J cells in Fig. 7 and 8.

The controls for the experiments using *Xenopus* oocytes are noninjected oocytes as indicated in the manuscript. These were included in most of the immunoblots showing that they were completely negative. This is because the corresponding immunostaining controls were not initially shown. Nevertheless, as requested by the reviewer these controls are now shown in Fig. 1 B, Fig. 4 D and G, Fig. 5 C, G and K, Fig. S2 A and Fig. S3 C, F and G. For the immunofluorescence microscopy on AR42J cells, the cells transfected with empty pcDNA3 (controls) are now also introduced in Fig. 8 C and Fig. 9 B.

4) Pages 28-29. Protein extraction and Immunoblot analysis. How do you assure that the same amount of protein is loaded for each sample? Also, in the Figures, a very narrow area is commonly shown for the Immunoblots while the full lane should ideally be presented. Furthermore, a size estimate for the band should be provided, this is not always the case. Related to this reflection, a very important aspect needs to be highlighted and that is the requirement of relating the function to the amount and stability of the protein, this is especially valid for the data in Fig. 2. This is a critical aspect to include in the results described on page 7.

We have revised all blots to be sure that the molecular mass markers are indicated. Also, full uncropped images from all blots and gels shown in the figures are now provided in the SourceData Files. For normalization of protein loading in all the immunoblots we used PDI, a constitutive protein of the YPM, as previously indicated (Fig. S1 D), or AMY. In Fig. 2 (now Fig. 3), protein stability was controlled by the amount of proten in the TM fraction (Fig. 3 A, bottom panel), while protein in the YPM was normalized to PDI (Fig. 3 B, bottom panel). These data show that protein levels of the different constructs in the oocyte PM and YPM were approximately equivalent. Also, the protein accumulation of the constructs in the PM or YPM is consistent with the permeabilities of the oocyte and YPs to the different substrates.

Reviewer #2

1) The authors have used a rat cell line to study the ZG localisation of YPD-containing aquaporins. From a translational perspective it would be very useful to determine if the same effect of the YPD motif is seen in human cells. Have the authors checked this or any plans to do so?

The YPD has not been tested in human cells because to our knowledge such a cell line is not commercially available. This is however an interesting aspect that we will consider to pursue in our future work.

2) In mammalian cells that do not have zymogen granules, what is the trafficking destination of YPD-containing aquaporins? Do the authors have any data to address this or could they speculate?

Again, although interesting, these experiments have not been done. We prefer not to speculate on this question in order to keep the manuscript as focused as possible.

3) Do the authors have any hypotheses or speculation about what is the component(s) of the membrane trafficking machinery that recognises the YPD motif?

This is a very relevant question that we are currently investigating using MS analysis of proteins that may coimmunoprecipitate with AQP12 as an initial approach.

4) Have the authors performed any bioinformatic analyses to look for YPD motifs across the proteome. It would be interesting to know whether this is an aquaporin-specific motif or if it is used by other classes of membrane proteins.

We did investigate this question using Blastp and found that the YPD domain is conserved in some other proteins such as insect intermembrane lipid transfer protein Vps13, mollusc N-acylglucosamine 2-epimerase, bacterial ATP-dependent DNA helicase, PD40 domain-containing proteins or type A sorting domain-containing protein. Interestingly, in humans we also found that partially conserved YPD are present in proteins residing in the membrane of intracellular vesicles (endosomes), endoplasmic reticulum or the nucleolar envelope, such as 1-

phosphatidylinositol 3-phosphate 5-kinase, importin-9, anterior gradient protein 3, patatin-like phospholipase domain-containing protein 7, AP-2 complex subunit alpha-1, or endoplasmic reticulum disulfide reductase DNAJC10. However, we considered that investigating the potential role of the domain in other proteins was beyond the scope of our study, which focuses on the discovery of the YPD and its role in AQP12 trafficking.

5) To assist the reader, the authors could mention that the unorthodox aquaporins are also referred to as "superaquaporins" in some literature.

This is now indicated (line 56).

Reviewer #3

1) With the identified motif located at the C-terminus of the AQP12 isoforms it is unfortunate that all used constructs contain a hemagglutinin epitope tag at the C-terminus as well. At which positions does the motif work (N-terminus, loops)? How does an epitope-free C-terminus affect the data?

To address this question, we have performed additional experiments using HA tag-free constructs. The data are reported in Fig. S3 which show that the tag does not affect the trafficking of wild-type human AQP12 and zebrafish Aqp12, or the AQP1 and Aqp1aa mutants carrying the YPD.

2) Which other yolk/zymogen-associated or other proteins carry the motif? Or do the authors expect an exclusive mechanism for AQP12?

Our initial analysis (see also Rev2#4) did not identify other YP/ZG-associated proteins that carry the YPD. This is an aspect that should be investigated in more detail in the future.

3) Besides action of the motif, phosphorylation in the N-terminal region appears to be required. What would a mechanism look like that addresses N- and C-terminal sites for trafficking? Are those sites close to each other and bind to the same translocating machinery? Is there a pool of native AQP12 protein somewhere in the cells before it is integrated into the organelles?

This is a very interesting question that unfortunately at this point we can not solve. For this, it would be necessary to determine the structure of AQP12, which currently remains challenging, but may now become more feasible based upon the current results. In addition, as mentioned earlier, during the revision process, a paper on the structure of AQP11 was published (Suzuki et al., 2026; cited in the manuscript). In the model proposed by these authors, the N- and C-termini of AQP11 are orientated in opposite sides of the membrane, and our *in silico* predicted models for human and zebrafish AQP12 trimers currently suggest the same orientation for the termini (Fig. S5). If such a structural arrangement is correct, then the N- and C-termini could not interact, and therefore phosphorylation of the N-terminus and the YPD would be two independent regulatory mechanisms. However, we prefer not to speculate on this subject until a clear structure is resolved for AQP12.

As for the pool of AQP12, in AR42J cells we observed that AQP12 channels lacking the YPD are not rapidly degraded, but possibly retained in the ER (Fig. 8 E). This suggests that the YPD may determine the pathway of AQP12 to the YPs and ZGs by acting as a signal for channel exit from the ER, further passage to the trans-Golgi network, and eventual incorporation into vesicles, with trafficking independently regulated by phosphorylation of the channel N-

terminus. These are however completely speculative mechanisms that have not been investigated in the present study, but we believe should be elucidated in the future.

4) Water permeability assays with yolk platelets were done using tritium-labeled water and an incubation time of 10 min. AQP facilitated water flux in such small organelles should be finished below or within a few seconds, with an AQP one would still expect a seconds timescale. How does the platelet membrane in the absence of an AQP exclude water exchange by transmembrane diffusion for such a long period of time?

We apologize because the units initially indicated in the graphs shown in Fig. 2 (now Fig. 3) were incorrect. The uptake of radioactive compound is per minute/µg protein (not per 10 minutes). Also note that when AQP12 is expressed at the oocyte PM (by substituting its C-terminus by that of AQP1) significant water transport occurs within 20 sec (Fig. 2, E and F). With the corrected units, it becomes apparent that the uptake of water by the YPs on a per µg protein basis is in fact more rapid than the whole oocyte (which contain about 100 µg total protein). This is likely due to the vastly greater membrane surface area of the yolk platelets used in the experiments, which were isolated from 30 oocytes.

5) The authors speculate about roles of an AQP in the yolk platelet and zymogen granule membranes. They state osmotic swelling as a requirement for membrane fusion. What is the origin of an osmotic gradient in the organelles and why would an AQP help in the process? This goes along with point 3, above. AQPs increase the water flux velocity up to one order of magnitude in the range of sub-second to seconds. However, this eventually results in the same steady-state situation.

Both ZGs and YPs are protein storage vesicles, and as such have low osmotic pressure due to the encapsulated large protein polymers. In YPs, this is likely more so due due to the fact that the yolk proteins are arranged in crystal-like lattices. In ZGs, is also established that the lumen concentration of ions is lower than that of the cytosol (Thévenod, 2015; cited in the manuscript). Consequently, to facilitate exocytosis of the proteins from their storage forms, a massive fluid uptake would be required to solubilize the proteins. As a result, the regulated trafficking of AQP12 to the vesicle membrane increases the permeability and ion channels are possibly activated to alter the osmotic pressure for such water uptake (Thévenod, 2015). A steady-state situation would not be achieved since the vesicles fuse with the plasma mebrane and the contents are released together with the water. The exact mechanisms remain however speculative as pointed out in the Discussion (lines 659-676).

6) Another prominent intracellular AQP is isoform 6. Its functionality is mainly ion permeability. In view of the points above, are there structural hints in the AQP12 family that point towards ion permeability?

As noted above, the structure of AQP12 has not been yet solved, and therefore this question remains unknown.

7) Urea is a peculiar AQP substrate due to its relatively large plus rigid and flat molecular structure, which requires special adaptations in the channel path. Is this type of permeability a by-product by chance?

The experiments demonstrate that both human and zebrafish AQP12 are permeable to urea. The molecular basis of such permeation again require a resolved structure not yet available.

April 14, 2026

RE: JCB Manuscript #202512040R

Joan Cerdà
Institut de Ciències del Mar

Dear Dr. Cerdà,

Thank you for submitting your revised manuscript entitled "A pan-vertebrate signaling motif controls the molecular function of the intracellular AQP12." The revised manuscript was assessed by two of the original reviewers. We would be happy to publish your paper in JCB pending final revisions necessary to address the one remaining reviewer comment and to meet our formatting guidelines (see details below).

You will see that Reviewer #1 notes that the claim that there are similar expression levels of different protein constructs the oocyte PM and YPM is not sufficiently supported due to absence of quantifications. We agree that it would be important to add such quantifications as well as explain the nature of additional bands seen on the blots as also requested by this reviewer.

To avoid unnecessary delays in the acceptance and publication of your paper, please read the following information carefully.

A. MANUSCRIPT ORGANIZATION AND FORMATTING:

Full guidelines are available on our Instructions for Authors page, https://jcb.rupress.org/submission-guidelines#revised.
Submission of a paper that does not conform to JCB guidelines will delay the acceptance of your manuscript.

1) Text limits: Character count for Articles is < 40,000, not including spaces. Count includes title page, abstract, introduction, results, discussion, and acknowledgments. Count does not include materials and methods, figure legends, references, tables, or supplemental legends.

2) Figure formatting: Articles may have up to 10 main text figures. Scale bars must be present on all microscopy images, including inset magnifications. Molecular weight or nucleic acid size markers must be included on all gel electrophoresis. Please add scale bars to inset magnifications in figure 2B/G and enlarge the scale bars in figures 8C & 9B-E/K, the current ones are too small.

Also, please avoid pairing red and green for images and graphs to ensure legibility for color-blind readers. If red and green are paired for images, please ensure that the particular red and green hues used in micrographs are distinctive with any of the colorblind types. If not, please modify colors accordingly or provide separate images of the individual channels.

3) Statistical analysis: Error bars on graphic representations of numerical data must be clearly described in the figure legend. The number of independent data points (n) represented in a graph must be indicated in the legend. Please indicate whether 'n' refers to technical or biological replicates (i.e. number of analyzed cells, samples or animals, number of independent experiments). If independent experiments with multiple biological replicates have been performed, we recommend using distribution-reproducibility SuperPlots (please see Lord et al., JCB 2020) to better display the distribution of the entire dataset, and report statistics (such as means, error bars, and P values) that address the reproducibility of the findings.

Statistical methods should be explained in full in the materials and methods. For figures presenting pooled data the statistical measure should be defined in the figure legends. Please also be sure to indicate the statistical tests used in each of your experiments (both in the figure legend itself and in a separate methods section) as well as the parameters of the test (for example, if you ran a t-test, please indicate if it was one- or two-sided, etc.). Also, if you used parametric tests, please indicate if the data distribution was tested for normality (and if so, how). If not, you must state something to the effect that "Data distribution was assumed to be normal but this was not formally tested."

4) Title: For syntax purposes we suggest slightly shortening the title to:
"A pan-vertebrate signaling motif controls the molecular function of intracellular AQP12."

5) Materials and methods: Should be comprehensive and not simply reference a previous publication for details on how an experiment was performed. Please provide full descriptions (at least in brief) in the text for readers who may not have access to referenced manuscripts. The text should not refer to methods "...as previously described." Please also indicate the type of membrane used for immunoblotting as well as describe acquisition and quantification methods.

6) For all cell lines, vectors, strains, constructs/cDNAs, etc. - all genetic material: please include database / vendor ID (e.g. Addgene, ATCC, etc.) or if unavailable, please briefly describe their basic genetic features, even if described in other published

work or gifted to you by other investigators (and provide references where appropriate). Please be sure to provide the sequences for all of your oligos: primers, si/shRNA, RNAi, gRNAs, etc. in the materials and methods. You must also indicate in the methods the source, species, and catalog numbers/vendor identifiers (where appropriate) for all of your antibodies, including secondary. If antibodies are not commercial, please add a reference citation if possible.

7) Microscope image acquisition: The following information must be provided about the acquisition and processing of images:
a. Make and model of microscope
b. Type, magnification, and numerical aperture of the objective lenses
c. Temperature
d. Imaging medium
e. Fluorochromes
f. Camera make and model
g. Acquisition software
h. Any software used for image processing subsequent to data acquisition. Please include details and types of operations involved (e.g., type of deconvolution, 3D reconstitutions, surface or volume rendering, gamma adjustments, etc.).

8) References: There is no limit to the number of references cited in a manuscript. References should be cited parenthetically in the text by author and year of publication. Abbreviate the names of journals according to PubMed. JCB formatting does not allow for supplementary references, please remove the ones at table S1 and add any non-duplicate references to the main reference list.

9) Supplemental materials: Articles may have up to 5 supplemental figures and 10 videos. Please also note that tables, like figures, should be provided as individual, editable files. A summary of all supplemental material should appear at the end of the Materials and methods section. Please include one brief sentence per item.

10) eTOC summary: A ~40-50 word summary that describes the context and significance of the findings for a general readership should be included on the title page. The statement should be written in the present tense and refer to the work in the third person. It should begin with "First author name(s) et al..." to match our preferred style.

11) Conflict of interest statement: JCB requires inclusion of a statement in the acknowledgements regarding competing financial interests. If no competing financial interests exist, please include the following statement: "The authors declare no competing financial interests." If competing interests are declared, please follow your statement of these competing interests with the following statement: "The authors declare no further competing financial interests."

12) A separate author contribution section is required following the Acknowledgments in all research manuscripts. All authors should be mentioned and designated by their first and middle initials and full surnames. We encourage use of the CRediT nomenclature (https://casrai.org/credit/).

13) ORCID IDs: ORCID IDs are unique identifiers allowing researchers to create a record of their various scholarly contributions in a single place. Please note that ORCID IDs are required for all authors. At resubmission of your final files, please be sure to provide your ORCID ID and those of all co-authors.

14) JCB requires authors to submit Source Data used to generate figures containing gels and Western blots with all revised manuscripts. This Source Data consists of fully uncropped and unprocessed images for each gel/blot displayed in the main and supplemental figures. For assays performed using capillary electrophoresis and/or immunoassay-based detection, authors should instead provide the electropherogram graph(s) for each experiment, plotting fluorescence/chemiluminescence intensity vs. molecular weight/size. Since your paper includes cropped gel and/or blot images, please be sure to provide one Source Data file for each figure gels, blots, and/or capillary electrophoresis assays along with your revised manuscript files. File names for Source Data figures should be alphanumeric without any spaces or special characters (i.e., SourceDataF#, where F# refers to the associated main figure number or SourceDataFS# for those associated with Supplementary figures). For traditional gels and blots, the lanes of the gels/blots should be labeled as they are in the associated figure, the place where cropping was applied should be marked (with a box), and molecular weight/size standards should be labeled wherever possible. For capillary electrophoresis assays, each trace in the graph should be color-coded and labeled to indicate which protein, gene, or sample is being measured (please try to avoid red/green combinations to accommodate our color-blind readers).

Source Data files will be directly linked to specific figures in the published article. Source Data Figures should be provided as individual PDF files (one file per figure). Authors should endeavor to retain a minimum resolution of 300 dpi or pixels per inch. Please review our instructions for export from Photoshop, Illustrator, and PowerPoint here: https://rupress.org/jcb/pages/submission-guidelines#revised.

15) Journal of Cell Biology now requires a data availability statement for all research article submissions. These statements will be published in the article directly above the Acknowledgments. The statement should address all data underlying the research presented in the manuscript. Please visit the JCB instructions for authors for guidelines and examples of statements at (https://rupress.org/jcb/pages/editorial-policies#data-availability-statement).

B. FINAL FILES:

Please upload the following materials to our online submission system. These items are required prior to acceptance. If you have any questions, contact JCB's Managing Editor, Lindsey Hollander (lhollander@rockefeller.edu).

-- An editable version of the final text (.DOC or .DOCX) is needed for copyediting (no PDFs).

-- High-resolution figure and MP4 video files: See our detailed guidelines for preparing your production-ready images, https://jcb.rupress.org/fig-vid-guidelines.

-- Cover images: If you have any striking images related to this story, we would be happy to consider them for inclusion on the journal cover. Submitted images may also be chosen for highlighting on the journal table of contents or JCB homepage carousel. Images should be uploaded as TIFF or EPS files and must be at least 300 dpi resolution.

It is JCB policy that if requested, original data images must be made available to the editors. Failure to provide original images upon request will result in unavoidable delays in publication. Please ensure that you have access to all original data images prior to final submission.

The license to publish form must be signed before your manuscript can be sent to production. A link to the license to publish form will be sent to the corresponding author only. Please take a moment to check your funder requirements before choosing the appropriate license.

Additionally, JCB encourages authors to submit a short video summary of their work. These videos are intended to convey the main messages of the study to a non-specialist, scientific audience. Think of them as an extended version of your abstract, or a short poster presentation. We encourage first authors to present the results to increase their visibility. The videos will be shared on social media to promote your work. For more detailed guidelines and tips on preparing your video, please visit https://rupress.org/jcb/pages/submission-guidelines#videoSummaries.

Thank you for your attention to these final processing requirements. Please revise and format the manuscript and upload materials within 14 days. If you need an extension for whatever reason, please let us know and we can work with you to determine a suitable revision period.

Please contact the journal office with any questions at cellbio@rockefeller.edu.

Thank you for this interesting contribution, we look forward to publishing your paper in Journal of Cell Biology.

Sincerely,

Elizabeth Miller, PhD
Monitoring Editor
Journal of Cell Biology

Dan Simon, PhD
Scientific Editor
Journal of Cell Biology

Reviewer #1 (Comments to the Authors (Required)):

The revised version of the manuscript "A pan-vertebrate signaling motif controls the molecular function of the intracellular AQP12" (Ms. No. 202512040R) is now evaluated again focusing on the raised points in the initial evaluation.
I had one critical point related to the functional evaluation of specific proteins analysed in whole cells or membrane extracts, where the function is related to the amount of produced protein as evaluated by immunoblot (R1#4): "Related to this reflection, a very important aspect needs to be highlighted and that is the requirement of relating the function to the amount and stability of the protein, this is especially valid for the data in Fig. 2".
The response from the authors on this point was "These data show that protein levels of the different constructs in the oocyte PM and YPM were approximately equivalent. Also, the protein accumulation of the constructs in the PM or YPM is consistent with the permeabilities of the oocyte and YPs to the different substrates".
I think one has to be a bit careful in stating that the levels of proteins are approximately equivalent without having any support from this analysing the intensity of the bands, especially since it appears to be rather different as evaluated by eye; for example comparing Hs-AQP12-HA and Hs-AQP1-12CT-HA produced in YPM, where the latter appears as multiple bands possibly related to less stable protein (New Fig. 3B).
I would have preferred a reflection on this aspect in the manuscript, but since I do not see that the conclusions would change, I

support publication of the manuscript in Journal of Cell Biology.

Reviewer #3 (Comments to the Authors (Required)):

All points have been addressed (even though the statement that a specifically resolved protein structure would be required to address several of the questions is not valid due to the sophisticated knowledge on AQP structure function relationships).

Editor comments

You will see that Reviewer #1 notes that the claim that there are similar expression levels of different protein constructs the oocyte PM and YPM is not sufficiently supported due to absence of quantifications. We agree that it would be important to add such quantifications as well as explain the nature of additional bands seen on the blots as also requested by this reviewer.

Please, see below our comments.

Reviewer #1

I had one critical point related to the functional evaluation of specific proteins analysed in whole cells or membrane extracts, where the function is related to the amount of produced protein as evaluated by immunoblot (R1#4): "Related to this reflection, a very important aspect needs to be highlighted and that is the requirement of relating the function to the amount and stability of the protein, this is especially valid for the data in Fig. 2".
The response from the authors on this point was "These data show that protein levels of the different constructs in the oocyte PM and YPM were approximately equivalent. Also, the protein accumulation of the constructs in the PM or YPM is consistent with the permeabilities of the oocyte and YPs to the different substrates".
I think one has to be a bit careful in stating that the levels of proteins are approximately equivalent without having any support from this analysing the intensity of the bands, especially since it appears to be rather different as evaluated by eye; for example comparing Hs-AQP12-HA and Hs-AQP1-12CT-HA produced in YPM, where the latter appears as multiple bands possibly related to less stable protein (New Fig. 3B).
I would have preferred a reflection on this aspect in the manuscript, but since I do not see that the conclusions would change, I support publication of the manuscript in Journal of Cell Biology.

As the reviewer requested, we have added a sentence in the manuscript regarding the stability and multiple bands of the HsAQP1-12CT construct (lines 180-181). We also agree that a statement concerning the quantity of protein would require supporting evidence, however we make no such statement in the manuscript. We think this confusion arose due to an error in our response in the rebuttal letter.

In the experiments shown in Fig. 3, quantitation of the protein in the blots was not needed, since the degree of permeability among the different constructs was not to be compared. These experiments were designed to test whether the constructs were localized in the correct subcellular membranes and were permeable or not to the different substrates. Therefore, quantitation would not change the conclusions, as the reviewer recognizes. Moreover, in this case band densitometry analysis would result in quantitation artifacts due to the detection of multiple bands for some of the constructs. Accordingly, the blots in Fig. 2 C and D and Fig. 3 A and B were only used to assure that the constructs were translated and localized in the expected fractions as is the case.
